# Aurora A exacerbates antibiotic-resistant *Staphylococcus aureus* infection through ASC phosphorylation

**DOI:** 10.1038/s44319-026-00918-x

**Published:** 2026-09-07

**Authors:** Hajime Yamauchi, Kei Sakamoto, Yasuyuki Matsuda, Yuko Tanishita, Akito Ariyoshi, Daisuke Sasaki, Yu Shimoyama, Akihiro Hasegawa, Takayoshi Yamauchi, Yoichiro Iwakura, Haruki Okamura, Shun’ichiro Taniguchi, Masafumi Takahashi, Akihiko Yoshimura, Katsunori Yanagihara, Gabriel Nuñez, Hideki Hara

**Affiliations:** 1 Department of Infectious Diseases, Division of Microbiology and Immunochemistry, Asahikawa Medical University, Hokkaido, Japan; 2 Department of Laboratory Medicine, Nagasaki University Graduate School of Biomedical Sciences, Nagasaki, Japan; 3 Department of Microbiology and Immunology, Yamaguchi University Graduate School of Medicine, Yamaguchi, Japan; 4 Department of Microbiology and Immunology, Keio University School of Medicine, Tokyo, Japan; 5 Department of Laboratory Medicine, Nagasaki University Hospital, Nagasaki, Japan; 6 Graduate School of Agricultural and Life Sciences, The University of Tokyo, Tokyo, Japan; 7 Laboratory of Tumor Immunology and Immunotherapy, Hyogo College of Medicine, Hyogo, Japan; 8 Department of Comprehensive Cancer Therapy, Shinshu University School of Medicine, Matsumoto, Nagano Japan; 9 Advanced Cancer Medicine for Gynecologic Cancer, Kagoshima University Graduate School of Medical and Dental Sciences, Kagoshima, Japan; 10 Division of Inflammation Research, Center for Molecular Medicine, Jichi Medical University, Tochigi, Japan; 11 Research Institute for Biomedical Science, Tokyo University of Science, Chiba, Japan; 12 Department of Pathology and Rogel Cancer Center, University of Michigan Medical School, Ann Arbor, MI USA; 13 Center for Infectious Disease Education and Research (CiDER), Osaka University, Osaka, Japan

**Keywords:** Immunology, Microbiology, Virology & Host Pathogen Interaction, Signal Transduction

## Abstract

Excessive inflammasome activation is increasingly recognized as a critical driver of pathology during bacterial infections, yet the pathogen-derived mechanisms that trigger dysregulated inflammasome responses remain poorly defined. Here, we identify a previously unrecognized role for the serine/threonine kinase Aurora A in promoting inflammasome activation during *Staphylococcus aureus* infection. We demonstrate that *S. aureus* activates JNK-dependent signaling in macrophages, leading to the phosphorylation of Aurora A. Activated Aurora A directly phosphorylates ASC, the inflammasome adaptor, on serine residues, enhancing ASC oligomerization and facilitating robust caspase-1 activation through both NLRP3 and AIM2 inflammasomes. This phosphorylation event stabilizes ASC speck formation and amplifies downstream IL-1β production, thereby intensifying inflammatory responses. Importantly, pharmacological inhibition or genetic disruption of Aurora A markedly suppresses inflammasome activation in vitro and in vivo, significantly reduces bacterial burdens and improves survival in mice infected with antibiotic-resistant *S. aureus*. These findings reveal Aurora A as a key host regulator exploited by *S. aureus* to potentiate inflammasome-driven inflammation and highlight inflammasome inhibition as a potential therapeutic strategy that augments host defense independently of antibiotic resistance.

## Synopsis

Uncontrolled inflammasome activation is a key driver of pathology during bacterial infection. This study identifies a previously unrecognized role for Aurora A in promoting inflammasome activation during antibiotic-resistant Staphylococcus aureus infection.

- Antibiotic-resistant Staphylococcus aureus activates Aurora A, which directly phosphorylates ASC.
- Aurora A promotes both NLRP3 and AIM2 inflammasome activation.
- NLRP3 and AIM2 exacerbate Staphylococcus aureus infection through IL-1β and IL-18 production.
- Inhibition of Aurora A suppresses IL-1β and IL-18 production and ameliorates antibiotic-resistant Staphylococcus infection.

## Abstract (web summary)

Uncontrolled inflammasome activation is a key driver of pathology during bacterial infection. This study identifies a previously unrecognized role for Aurora A in promoting inflammasome activation during antibiotic-resistant Staphylococcus aureus infection.

## Graphical abstract

**Figure:**
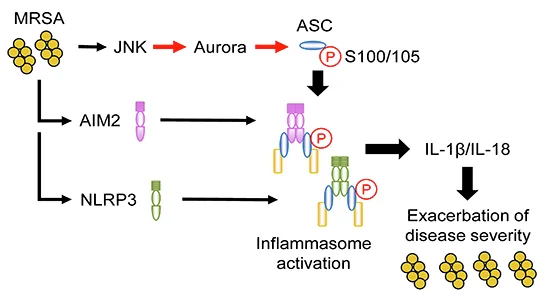

## Introduction

*Staphylococcus aureus* is a major human pathogen responsible for a broad spectrum of diseases ranging from skin and soft tissue infections to life-threatening conditions such as bacteremia, pneumonia, and sepsis (Bennett and Thomsen, 2020). A major challenge in controlling *S. aureus* infection is the rapid and continuous emergence of antimicrobial resistance, including methicillin-resistant *S. aureus* (MRSA) and vancomycin-resistant *S. aureus* strains. These resistant pathogens cause severe and often refractory infections in both community and healthcare settings, underscoring the urgent need for alternative therapeutic strategies that do not rely solely on conventional antibiotics.

A growing body of evidence demonstrates that *S. aureus* can trigger pathological inflammation through the activation of host inflammasomes. The NLR family pyrin domain-containing 3 (NLRP3) inflammasome is activated by hemolysins, potassium efflux, and *S. aureus*-derived extracellular vesicles (An et al, 2019; Craven et al, 2009; Munoz-Planillo et al, 2009; Wang et al, 2020). In addition to NLRP3, recent studies revealed that NLRP6 is activated during *S. aureus* infection through the recognition of lipoteichoic acid, a cell wall component of Gram-positive bacteria (Ghimire et al, 2018; Hara et al, 2018). These inflammasomes converge on the adapter, the apoptosis-associated speck-like protein containing caspase recruitment domain (ASC), which nucleates caspase-1 activation, promoting maturation of proinflammatory cytokines, interleukin-1β (IL-1β) and IL-18, and inducing pyroptotic cell death through the cleavage of the pore-forming protein gasdermin D (Kayagaki et al, 2015; Shi et al, 2015). Although inflammasomes are important for antimicrobial defense, excessive or dysregulated activation can exacerbate disease pathology in infections caused by *S. aureus*, *Listeria monocytogenes*, *Bacillus cereus*, *Mycobacterium tuberculosis*, and SARS-CoV-2 (Ghimire et al, 2018; Hara et al, 2018; Mathur et al, 2019; Paudel et al, 2019; Rodrigues et al, 2021; Subbarao et al, 2020). This dual role highlights the therapeutic potential of selectively modulating inflammasome activity rather than globally suppressing innate immunity. Indeed, clinical trials testing NLRP3 inhibitors for inflammatory diseases and COVID-19 highlight the translational momentum in this field. Despite these advances, relatively little is known about the signaling pathways that regulate ASC-dependent inflammasome activation during bacterial infection. ASC phosphorylation has emerged as a key regulatory mechanism. Previous studies have revealed that its tyrosine phosphorylation at Y144 is indispensable for inflammasome activation (Chung et al, 2016; Hara et al, 2013), yet the involvement of serine/threonine kinases and the broader regulatory network controlling ASC activation remains poorly understood.

Here, we identify Aurora kinase A, traditionally studied for its role in mitosis, as a previously unrecognized regulator of inflammasome activation during *S. aureus* infection. Tang et al reported that Aurora A promotes psoriasis-associated inflammation by regulating AIM2 degradation (Tang et al, 2021). In addition, a recent study showed that ASC forms specks at the centrosome, a known site of Aurora A localization (Wang et al, 2026). We show that Aurora A directly phosphorylates ASC on serine residues, promoting ASC oligomerization and activation of both the NLRP3 and absent in melanoma 2 (AIM2) inflammasomes. We further demonstrate that Aurora A activation occurs downstream of JNK signaling in macrophages, and that pharmacological inhibition of Aurora kinases markedly improves outcomes in murine models of MRSA infection. These findings reveal an unexpected role for Aurora A in innate immunity and support the potential of targeting the JNK-Aurora-ASC axis as an alternative therapeutic strategy, particularly in the context of rising antimicrobial resistance.

## Results

### Aurora A directly phosphorylates ASC

**Figure 1:**
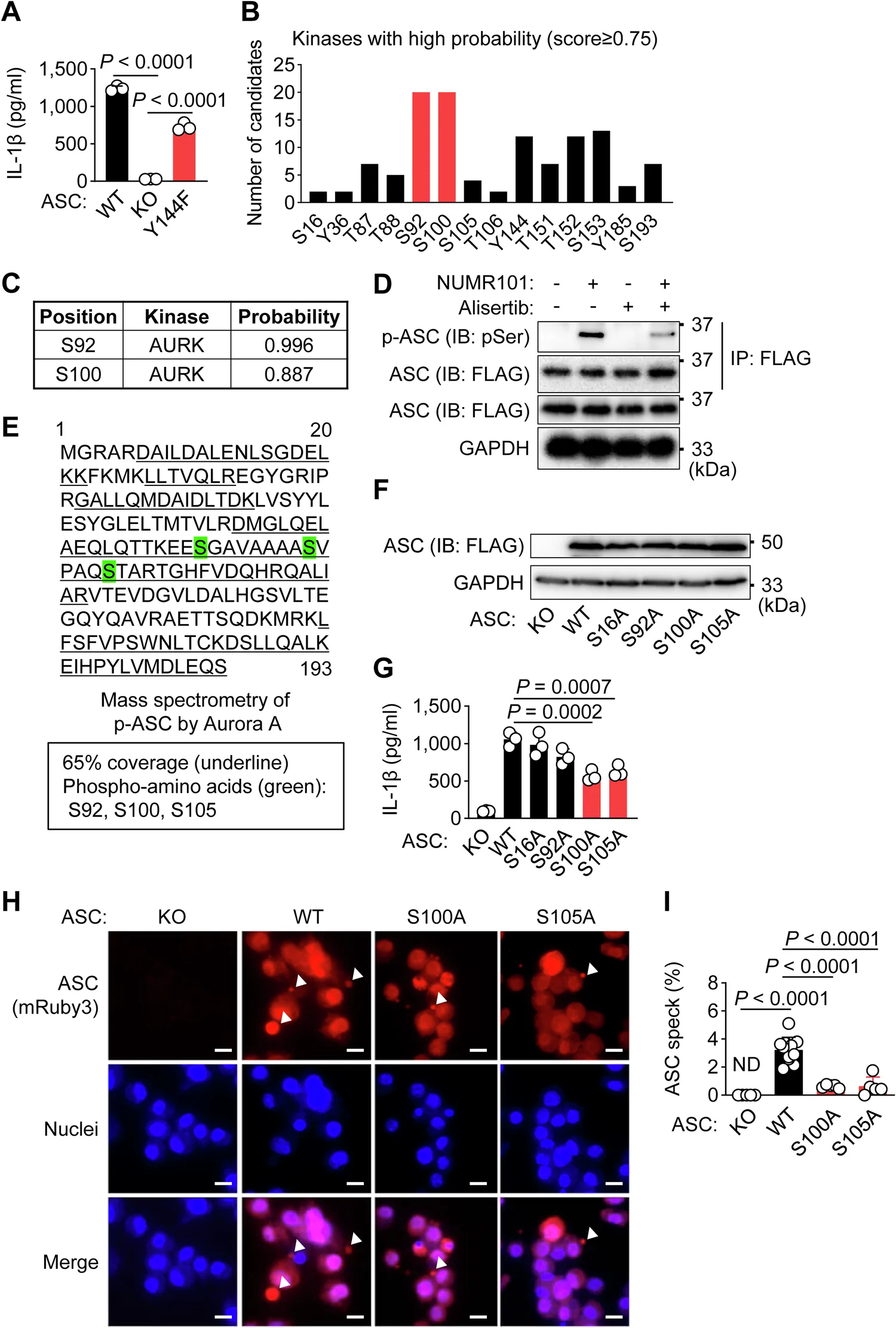
Aurora A directly phosphorylates ASC. (**A**) Cytokine levels in culture supernatants of ASC-reconstituted macrophages after infection with *S. aureus* 8325-4 strain. Bars represent the mean ± SD of three biological replicates. Statistical analysis was performed using one-way ANOVA followed by Tukey’s multiple comparison test. *P* < 0.0001, *P* < 0.0001. (**B**,** C**) Predicted phosphorylation sites in the amino acid sequence of mouse ASC by the program KinasePhos 3.0. (**B**) Kinase candidates with high probability (score ≥0.75) were selected for each phosphorylation site and (**C**) predicted ASC phosphorylation sites and their probabilities for Aurora kinase (AURK). (**D**) Immunoblotting of immunoprecipitated cell lysates of immortalized *Pycard*^−/−^ BMDMs reconstituted with ASC-SFP after infection with *S. aureus* NUMR101 at MOI = 25 for 12 h. Cells were treated with or without 10 μM Alisertib 30 min before infection. (**E**) Mass spectrometric analysis of purified ASC treated with Aurora A. (**F**) Immunoblotting of cell lysates without infection and (**G**) cytokine levels in cell supernatants of immortalized ASC-3xFLAG-mRuby3 mutant-reconstituted *Pycard*^−/−^ BMDMs infected with *S. aureus* NUMR101 at MOI = 10 for 12 h. Bars represent the mean ± SD of three biological replicates. Statistical analysis was performed using one-way ANOVA followed by Dunnett’s multiple comparison test. *P* = 0.0002, *P* = 0.0007. (**H**,** I**) Fluorescence imaging of the mutant macrophages infected with *S. aureus* NUMR101 at MOI = 10 for 15 h. Arrowheads indicate ASC specks. Scale bars, 10 μm. Bars represent the mean ± SD of (WT) eleven vs (KO, S100A, S105A) five biological replicates. Statistical analysis was performed using one-way ANOVA followed by Dunnett’s multiple comparison test. *P* < 0.0001, *P* < 0.0001, *P* < 0.0001. ND not detected. Source data are available online for this figure.

**Figure EV1:**
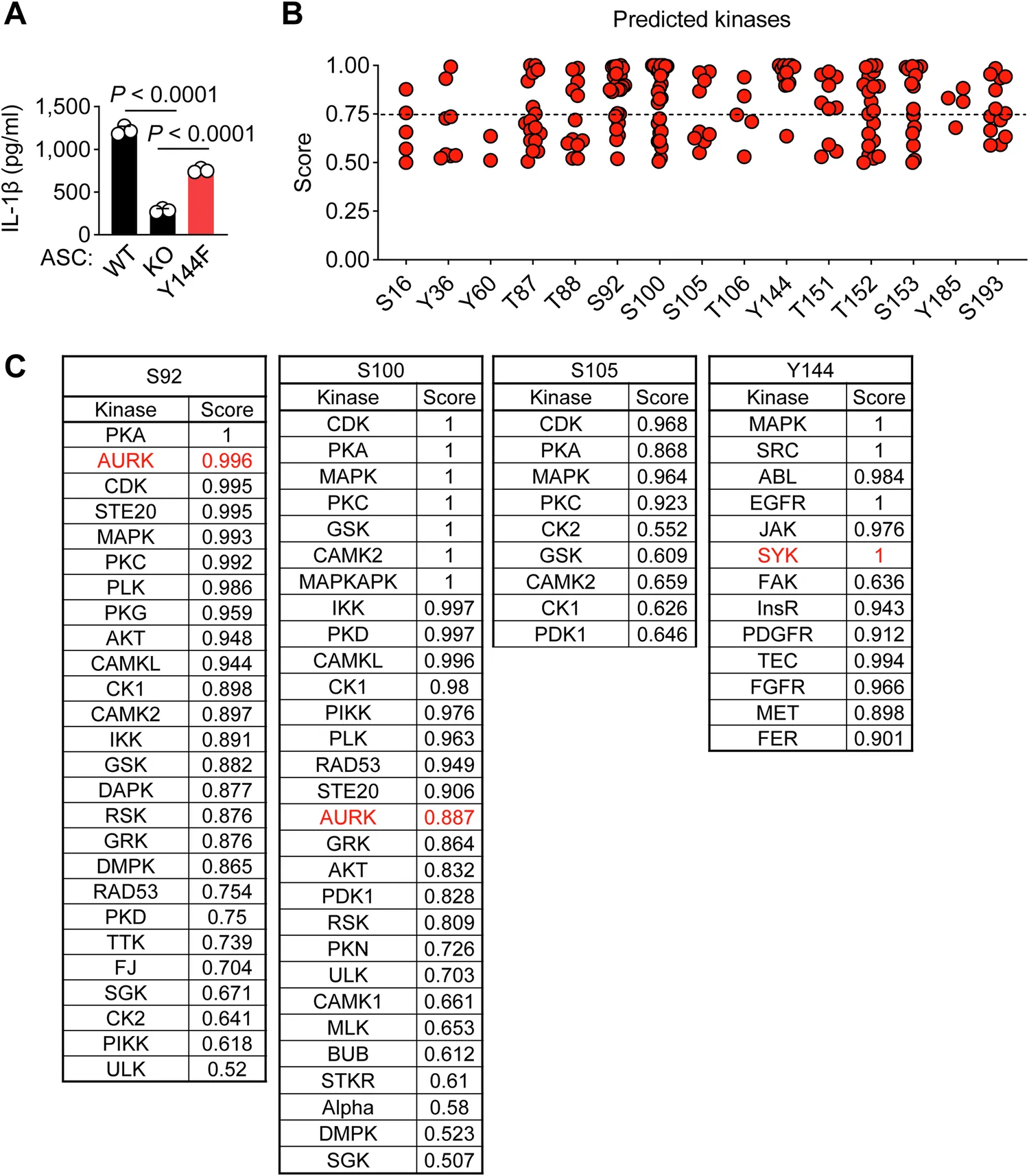
Prediction of phosphorylation sites in ASC. (**A**) Cytokine levels in culture supernatants of ASC-reconstituted macrophages after infection with *S. aureus* NUMR101 strain. Bars represent the mean ± SD of three biological replicates. Statistical analysis was performed using one-way ANOVA followed by Tukey’s multiple comparison test. *P* < 0.0001, *P* < 0.0001. (**B**,** C**) Kinases for murine ASC and phosphorylation sites were predicted using KinasePhos 3.0. Phosphorylation sites are shown on the x-axis, and the probability score is shown on the y-axis. Each dot indicates a kinase for the corresponding amino acid.

Previous reports showed that ASC is indispensable for inflammasome activation, and suggested the importance of phosphorylation of ASC at tyrosine 144 (Y144) for inflammasome activation in response to nigericin or NLRP3 R258W, a disease-associated mutation of NLRP3 (Chung et al, 2016; Hara et al, 2013). To evaluate whether phosphorylation of ASC at Y144 is important for inflammasome activation after *S. aureus* infection, we prepared macrophages expressing an ASC mutant in which Y144 was replaced by phenylalanine (Y144F), and infected wild-type and the mutant macrophages, as well as *Pycard*^-/-^ macrophages, with *S aureus* 8325-4 or NUMR101, an MRSA strain isolated from the blood of infected patients (Yanagihara et al, 2006). Notably, ASC Y144F-expressing macrophages were impaired in the release of IL-1β in response to *S. aureus* infection, but they produced more IL-1β than *Pycard*^-/-^ macrophages (Figs. 1A and EV1A). These data suggested that inflammasome activation may be regulated in an unknown manner, which may involve phosphorylation of ASC at a site other than Y144. To investigate possible sites of ASC phosphorylation, we searched phosphorylation sites on ASC and corresponding kinases using the KinasePhos 3.0 platform, which predicted two serine amino acid residues, S92 and S100, to be phosphorylated on ASC (Ma et al, 2023) (Figs. 1B and EV1B,C). Notably, Aurora kinase was listed as one of the potential kinases capable of phosphorylating ASC on S92 and S100 (Fig. 1C). To determine whether Aurora kinase A phosphorylates serine on ASC, we generated macrophages expressing FLAG-tagged ASC and infected the macrophages with *S. aureus*. We found that ASC was phosphorylated on serine residues after *S. aureus* infection, which was diminished by treatment with the Aurora kinase inhibitor Alisertib (Fig. 1D). To clarify whether Aurora A directly phosphorylates ASC, we conducted mass spectrometric analysis using purified ASC and Aurora A. Phosphorylation was detected at S105 of ASC in addition to the predicted S92 and S100 of ASC (Fig. 1E). To examine whether inflammasome activation induced by *S. aureus* infection is regulated via phosphorylation of ASC on S100 and S105, we constructed *Pycard*^-/-^ immortalized bone marrow-derived macrophages (BMDMs) expressing a series of ASC point mutants in which amino acid residues predicted to be phosphorylated were replaced with alanine (Fig. 1F). Macrophages expressing ASC S100A or S105A secreted less IL-1β in response to *S. aureus* infection than that of ASC wild-type, S16A, and S92A mutants (Fig. 1G). ASC speck formation was also reduced in *Pycard*^−/−^ BMDMs reconstituted with ASC S100A or S105A compared with that of macrophages expressing wild-type ASC (Fig. 1H,I). Additionally, IL-1β production from *Pycard*^-/-^ immortalized macrophages reconstituted with ASC S100A or S105A was not further suppressed by treatment with an Aurora kinase inhibitor (Appendix Fig. S1A). To examine potential redundancy between these serine phosphorylation sites, we generated macrophages expressing an ASC S100/105 A double mutant. Compared with the S100A single mutant, the ASC S100/105 A double mutant showed further reductions in IL-1β production (Appendix Fig. S1B). These results suggest that Aurora A phosphorylates ASC at S100 and S105 to regulate inflammasome activation.

### Aurora A regulates ASC speck formation and inflammasome activation

**Figure 2:**
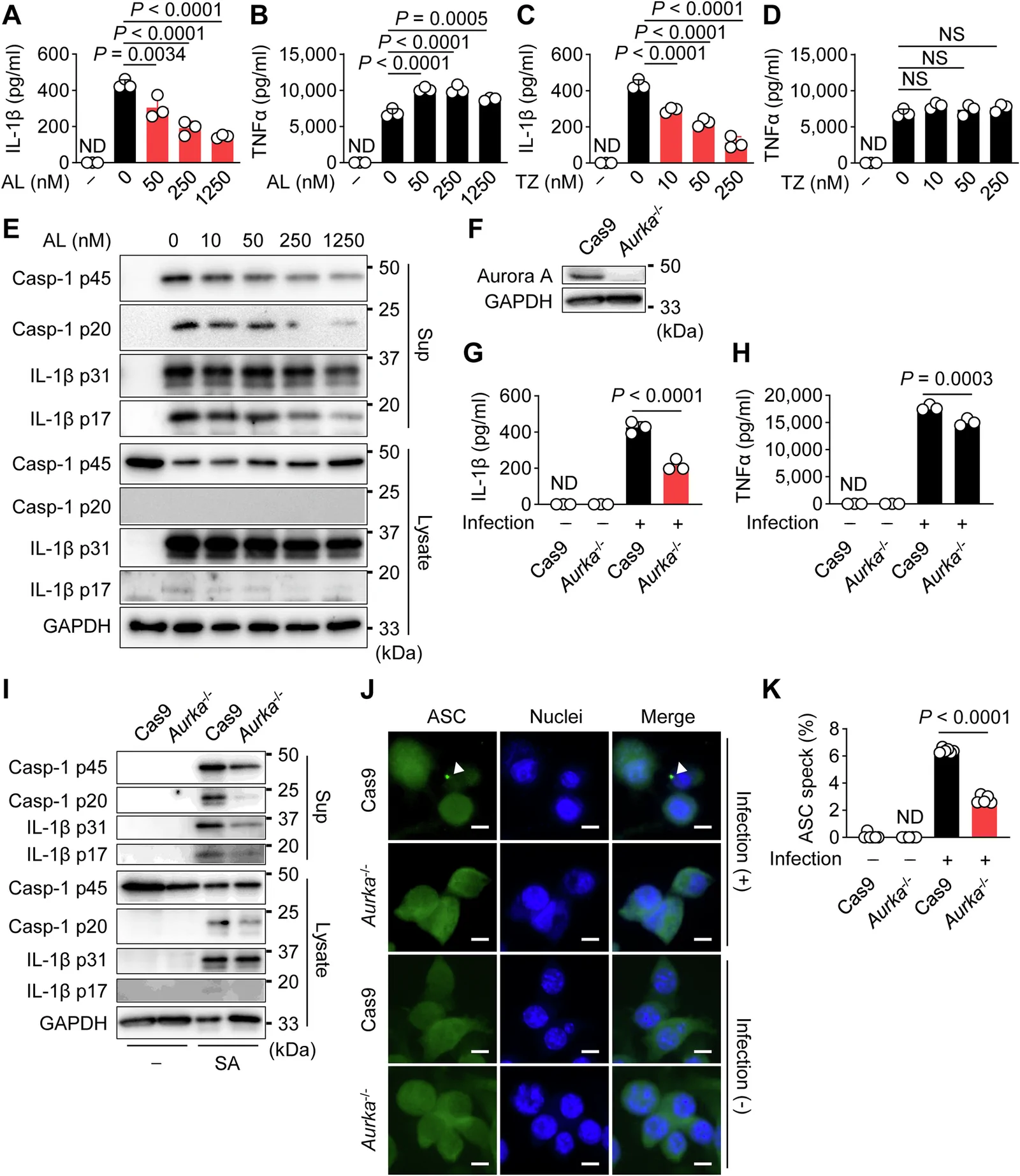
Aurora A regulates ASC speck formation and inflammasome activation. (**A**‒**D**) Cytokine levels in culture supernatants and (**E**) immunoblotting of cell lysates of peritoneal macrophages infected with *S. aureus* NUMR101 at MOI = 25 for 18 h. Cells were treated with Aurora kinase inhibitors, Alisertib (AL) or Tozasertib (TZ) 30 min before infection. Bars represent the mean ± SD of three biological replicates. Statistical analysis was performed using one-way ANOVA followed by Dunnett’s multiple comparison test. (**A**) *P* = 0.0034, *P* < 0.0001, *P* < 0.0001, (**B**) *P* < 0.0001, *P* < 0.0001, *P* = 0.0005, (**C**) *P* < 0.0001, *P* < 0.0001, *P* < 0.0001. NS no significant difference. ND not detected. (**F**) Immunoblotting of cell lysates of Cas9-expressing immortalized wild-type or Aurora A-deficient BMDMs without infection. (**G**,** H**) Cytokine levels in cell supernatants and (**I**) immunoblotting of cell lysates (Lysate) and supernatants (Sup) of Cas9-expressing immortalized wild-type or Aurora A-deficient BMDMs infected with *S. aureus* NUMR101 at MOI = 50 for 18 h. Bars represent the mean ± SD of three biological replicates. Statistical analysis was performed using one-way ANOVA followed by Tukey’s multiple comparison test. (**G**) *P* < 0.0001, (**H**) *P* = 0.0003. (**J**,** K**) Immunostaining analysis of immortalized Aurora A-deficient BMDMs infected with *S. aureus* NUMR101 at MOI = 50 for 12 h. Arrowheads indicate ASC specks. Scale bars, 10 μm. Bars represent the mean ± SD of (Cas9) six vs (*Aurka*^−/−^) five biological replicates. Statistical analysis was performed using one-way ANOVA followed by Tukey’s multiple comparison test. *P* < 0.0001. Source data are available online for this figure.

**Figure EV2:**
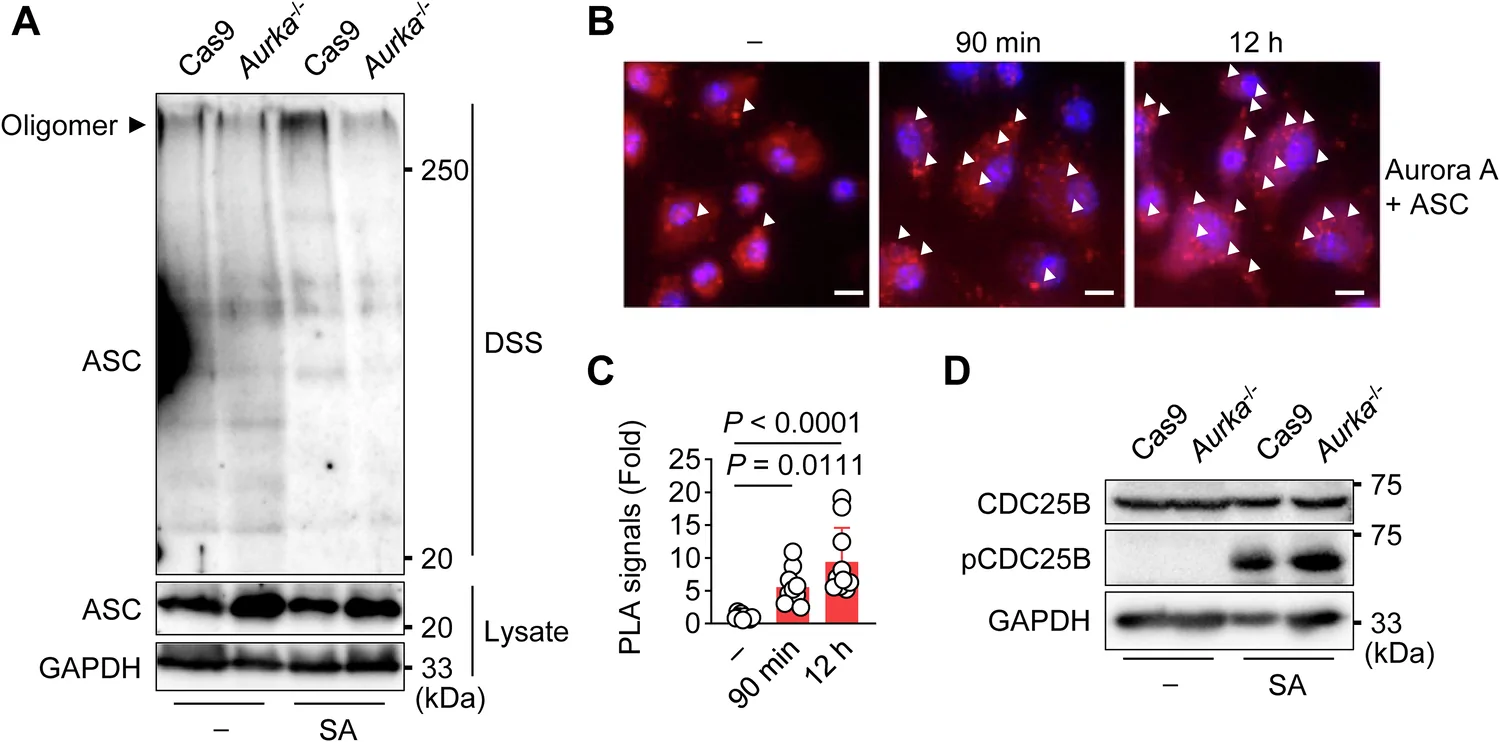
Aurora A interacts with ASC during *S. aureus* infection. (**A**) Immunoblotting of Triton X-100-soluble lysate (Lysate) and -insoluble fractions treated with DSS (DSS) of immortalized Aurora A-deficient BMDMs infected with *S. aureus* NUMR101 at MOI = 50 for 12 h. (**B**,** C**) In situ PLA of primary BMDMs infected with *S. aureus* NUMR101 at MOI = 50 for the indicated times. Arrowheads indicate interactions of Aurora A and ASC. Bars represent the mean ± SD of ten biological replicates. Statistical analysis was performed using one-way ANOVA followed by Dunnett’s multiple comparison test. *P* = 0.0111, *P* < 0.0001. (**D**) Immunoblotting of cell lysates of Cas9-expressing immortalized wild-type or Aurora A-deficient BMDMs infected with *S. aureus* NUMR101 at MOI = 50 for 18 h.

Next, we examined whether Aurora A regulates inflammasome activation after *S. aureus* infection. IL-1β production was suppressed by incubation of macrophages with Alisertib, a selective Aurora A kinase inhibitor, but not other kinase inhibitors we tested, in a dose-dependent manner (Fig. 2A; Appendix Fig. S2). In contrast, TNFα production was not affected by the addition of Alisertib (Fig. 2B). Comparable results were observed by treating macrophages with Tozasertib, another Aurora kinase inhibitor (Fig. 2C,D). Alisertib also inhibited caspase-1 cleavage in a dose-dependent manner (Fig. 2E). These results indicate that Aurora A regulates inflammasome activation induced by *S. aureus* infection. To confirm that Aurora A is important for the regulation of inflammasome activation, we generated Aurora A knockout macrophages by CRISPR/Cas9 genome editing (Fig. 2F; Appendix Fig. S3). Aurora A-deficient macrophages showed reduced IL-1β secretion compared to control macrophages, while TNFα secretion did not decrease as markedly as IL-1β (Fig. 2G,H). The Aurora A-deficient macrophages also showed impaired caspase-1 processing after *S. aureus* infection (Fig. 2I). Moreover, ASC speck formation was markedly reduced in the *Aurka*^−/−^ macrophages (Fig. 2J,K). The Aurora A-dependent ASC oligomerization was also confirmed by DSS crosslinking of Triton X-100-insoluble fractions (Fig. EV2A). Additionally, an interaction of Aurora A with ASC was visualized in primary macrophages during *S. aureus* infection by in situ Proximity Ligation Assay (PLA) (Fig. EV2B,C). Because Aurora A has been reported to phosphorylate CDC25B at the centrosome during mitosis (Cazales et al, 2005; Dutertre et al, 2004), we examined CDC25B phosphorylation in our Aurora A-deficient macrophages. We found that CDC25B is expressed in macrophages before infection, and its expression level was comparable between control and Aurora A knockout macrophages (Fig. EV2D). In addition, although *S. aureus* infection induced CDC25B phosphorylation in Cas9 cells, a similar level of phosphorylation was observed in *Aurka*^−/−^ macrophages (Fig. EV2D). These findings suggest that CDC25B phosphorylation induced by *S. aureus* infection occurs independently of Aurora A in macrophages. Thus, Aurora A appears to regulate inflammasome activation through a mechanism distinct from CDC25B phosphorylation in this context.

### Aurora A is activated downstream of JNK during *S. aureus* infection

**Figure 3:**
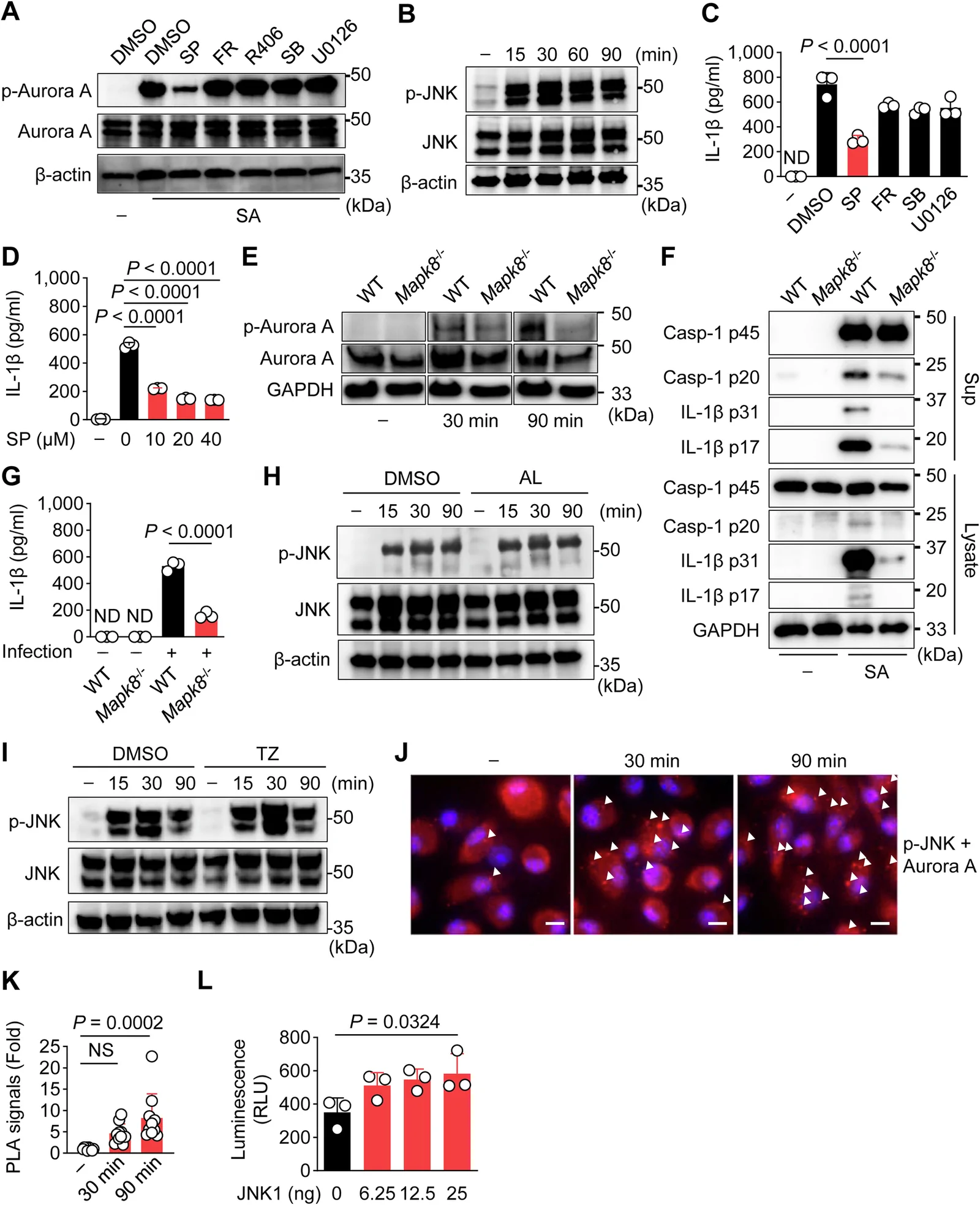
Aurora A is activated downstream of JNK during *S. aureus* infection. (**A**,** B**) Immunoblotting of cell lysates and (**C**,** D**) cytokine levels in culture supernatants of peritoneal macrophages infected with *S. aureus* NUMR101 at MOI = 25 for (**A**) 1 h, (**C**,** D**) 18 h or indicated times. Cells were treated with SP600125 (SP, JNK inhibitor, 40 μM), FR180204 (FR, ERK inhibitor, 10 μM), R406 (Syk inhibitor, 1 μM), SB203580 (SB, p38 inhibitor, 10 μM), and U0126 (MEK inhibitor, 20 μM) 30 min before infection. Bars represent the mean ± SD of three biological replicates. Statistical analysis was performed using one-way ANOVA followed by Dunnett’s multiple comparison test. (**C**) *P* < 0.0001, (**D**) *P* < 0.0001, *P* < 0.0001, *P* < 0.0001. (**E**,** F**) Immunoblotting of cell lysates and (**G**) cytokine levels in cell supernatants of immortalized wild-type (WT) or *Mapk8*^−/−^ BMDMs infected with *S. aureus* NUMR101 at MOI = 50 for 15 h or indicated times. Bars represent the mean ± SD of three biological replicates. Statistical analysis was performed using one-way ANOVA followed by Tukey’s multiple comparison test. *P* < 0.0001. ND not detected. (**H**,** I**) Immunoblotting of cell lysates of peritoneal macrophages infected with *S. aureus* NUMR101 at MOI = 25 for the indicated times. Cells were treated with Alisertib (AL Aurora A inhibitor, 1250 nM) and Tozasertib (TZ, pan-Aurora inhibitor, 250 nM) 30 min before infection. (**J**,** K**) In situ PLA of primary BMDMs infected with *S. aureus* NUMR101 at MOI = 25 for the indicated times. Arrowheads indicate interactions of p-JNK and Aurora A. Bars represent the mean ± SD of ten biological replicates. Statistical analysis was performed using one-way ANOVA followed by Dunnett’s multiple comparison test. *P* = 0.0002. NS no significant difference. (**L**) In vitro kinase assay using JNK1 (0, 6.25, 12.5, or 25 ng/well) and Aurora A (15 ng/well). ADP production was measured with ADP-Glo reagent. Bars represent the mean ± SD of three biological replicates. Statistical analysis was performed using one-way ANOVA followed by Dunnett’s multiple comparison test. *P* = 0.0324. Source data are available online for this figure.

We performed experiments to assess how Aurora A is activated in response to *S. aureus* infection. We found that treatment of macrophages with SP600125, a JNK inhibitor, but not several kinase inhibitors tested, reduced the levels of Aurora A phosphorylation (Fig. 3A). Since JNK was activated in macrophages after *S. aureus* infection (Fig. 3B), we further assessed JNK as a regulator of Aurora A in inflammasome activation. To this end, kinase inhibitors were added to the macrophages infected with *S. aureus* and IL-1β production was measured. SP600125, but not several kinase inhibitors tested, reduced IL-1β production induced by *S. aureus* infection (Fig. 3C). In addition, treatment with SP600125 showed dose-dependent suppression of IL-1β and TNFα production (Fig. 3D; Appendix Fig. S4), as reported that *S. aureus* infection induces JNK phosphorylation downstream of Toll-like receptor 2 (TLR2), leading to NF-κB activation (Fang et al, 2014; Watanabe et al, 2007). To confirm that JNK is involved in Aurora A-mediated regulation of inflammasome activation, we assessed the activation of Aurora A in macrophages derived from mice deficient in JNK1 encoded by *Mapk8*. We found that Aurora A phosphorylation, caspase-1 cleavage, and IL-1β production induced by *S. aureus* infection were suppressed by JNK1 deficiency (Fig. 3E‒G). Unlike *Aurka*^−/−^ macrophages (Fig. 2I), *Mapk8*^−/−^ cells showed reduced pro-IL-1β expression as well as impaired caspase-1 activation, indicating that JNK signaling is involved in both the priming and activation steps of inflammasome activation (Fig. 3F). Importantly, Aurora kinase inhibitors did not attenuate JNK activation (Fig. 3H,I), indicating that Aurora A acts downstream of JNK to regulate inflammasome activation. In fibroblasts and ovarian granulosa cells, JNK has been reported to promote entry into mitosis through phosphorylation of Aurora B (Oktay et al, 2008). However, the immunological functions of Aurora kinases in macrophages have remained unclear. To examine whether p-JNK1 interacts with Aurora A under endogenous conditions, we performed in situ PLA. The interaction between p-JNK1 and Aurora A was detected in response to *S. aureus* infection (Fig. 3J,K). Moreover, the results of the in vitro kinase assay indicate that JNK1 is capable of directly phosphorylating Aurora A (Fig. 3L). These results indicate that JNK1 interacts with Aurora A and phosphorylates it.

### Aurora A and JNK1 regulate the NLRP3 and AIM2 inflammasomes in *S. aureus*-infected macrophages

**Figure 4:**
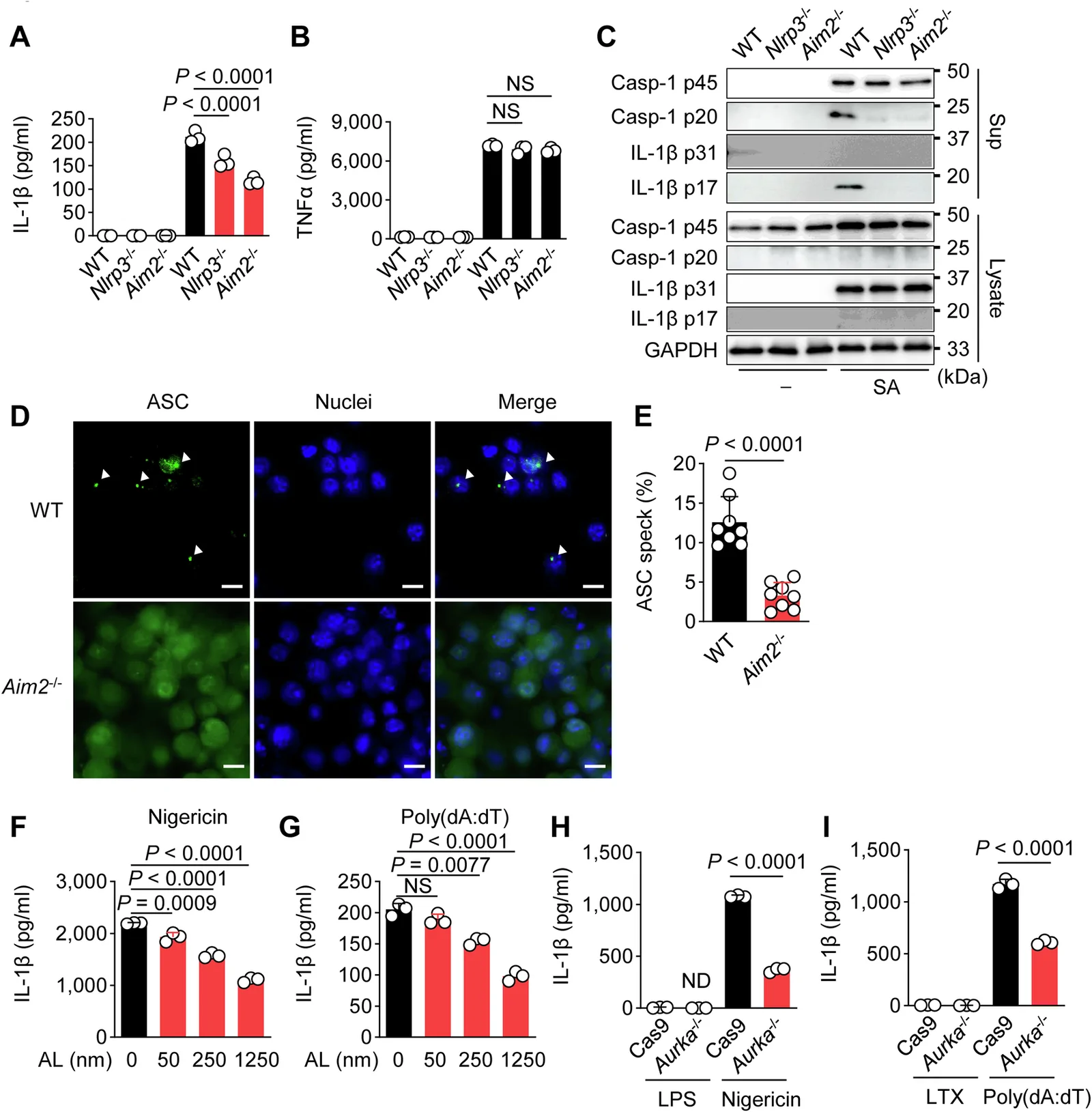
*S. aureus* activates NLRP3 and AIM2 inflammasomes in macrophages. (**A**,** B**) Cytokine levels in cell supernatants and (**C**) immunoblotting of wild-type, *Nlrp3*^−/−^, and *Aim2*^−/−^ primary BMDMs infected with *S. aureus* 8325-4 at MOI = 25 for 9 h. Bars represent the mean ± SD of three biological replicates. Statistical analysis was performed using one-way ANOVA followed by Tukey’s multiple comparison test. (**A**) *P* < 0.0001, *P* < 0.0001. NS no significant difference. (**D**,** E**) Immunostaining analysis of immortalized wild-type and *Aim2*^−/−^ BMDMs infected with *S. aureus* 8325-4 at MOI = 50 for 15 h. Arrowheads indicate ASC specks. Scale bars, 10 μm. Bars represent the mean ± SD of eight biological replicates. Statistical analysis was performed using a two-tailed *t*-test. *P* < 0.0001. (**F**–**I**) Cytokine levels in cell supernatants of LPS-primed (**F**,** G**) peritoneal macrophages or (**H**,** I**) Cas9-expressing immortalized wild-type or Aurora A-deficient BMDMs stimulated with nigericin for 60 min or poly(dA:dT) plus Lipofectamine LTX for 120 min. (**F**,** G**) Cells were treated with inhibitors 30 min before stimulation. Bars represent the mean ± SD of three biological replicates. Statistical analysis was performed using one-way ANOVA followed by (**F**,** G**) Dunnett’s multiple comparison test or (**H**,** I**) Tukey’s multiple comparison test, two-tailed *t*-test. (**F**) *P* = 0.0009, *P* < 0.0001, *P* < 0.0001. (**G**) *P* = 0.0077, *P* < 0.0001, (**H**) *P* < 0.0001, (**I**) *P* < 0.0001. NS no significant difference. ND not detected. Source data are available online for this figure.

Next, we determined the inflammasomes that are regulated by Aurora A in response to *S. aureus* infection. We infected primary BMDMs from wild-type, *Nlrp3*^−/−^ and *Aim2*^−/−^, mice with *S. aureus* and assessed IL-1β release and caspase-1 processing. Consistent with previous studies (Craven et al, 2009; Melehani et al, 2015; Munoz-Planillo et al, 2009), levels of IL-1β, but not TNFα, were impaired in *Nlrp3*^-/-^ macrophages infected with *S. aureus* 8325-4 or MRSA strain NUMR101 (Fig. 4A,B; Appendix Fig. S5A,B). As observed in a previous study (Feng et al, 2022), AIM2-deficient BMDMs also produced less IL-1β, which were lower than those produced by *Nlrp3*^−/−^ macrophages (Fig. 4A; Appendix Fig. S5A). In addition, formation of the p20 active form of caspase-1, which is the critical enzyme for IL-1β production, was reduced in macrophages deficient in NLRP3 or AIM2 (Fig. 4C; Appendix Fig. S5C). Consistent with these results, ASC speck formation was diminished in *Aim2*^−/−^ BMDMs (Fig. 4D,E). To clarify whether the JNK-Aurora signaling axis was involved in the regulation of both the NLRP3 and AIM2 inflammasomes, macrophages were stimulated with nigericin and poly(dA:dT), the specific activators of the NLRP3 and AIM2 inflammasomes, respectively. Alisertib, a selective Aurora A inhibitor, reduced IL-1β production in macrophages stimulated with nigericin or poly(dA:dT) (Fig. 4F,G). Moreover, Aurora A-deficient macrophages secreted less IL-1β compared with Cas9-expressing parent cells in response to nigericin or poly(dA:dT) (Fig. 4H,I). JNK1-depleted macrophages also showed impaired IL-1β production induced by NLRP3 and AIM2 stimulants (Appendix Fig. S6). These data indicate that Aurora A and JNK1 regulate both the NLRP3 and AIM2 inflammasomes.

### IL-1β is secreted in response to bacterial DNA release in macrophages infected with *S. aureus*

**Figure 5:**
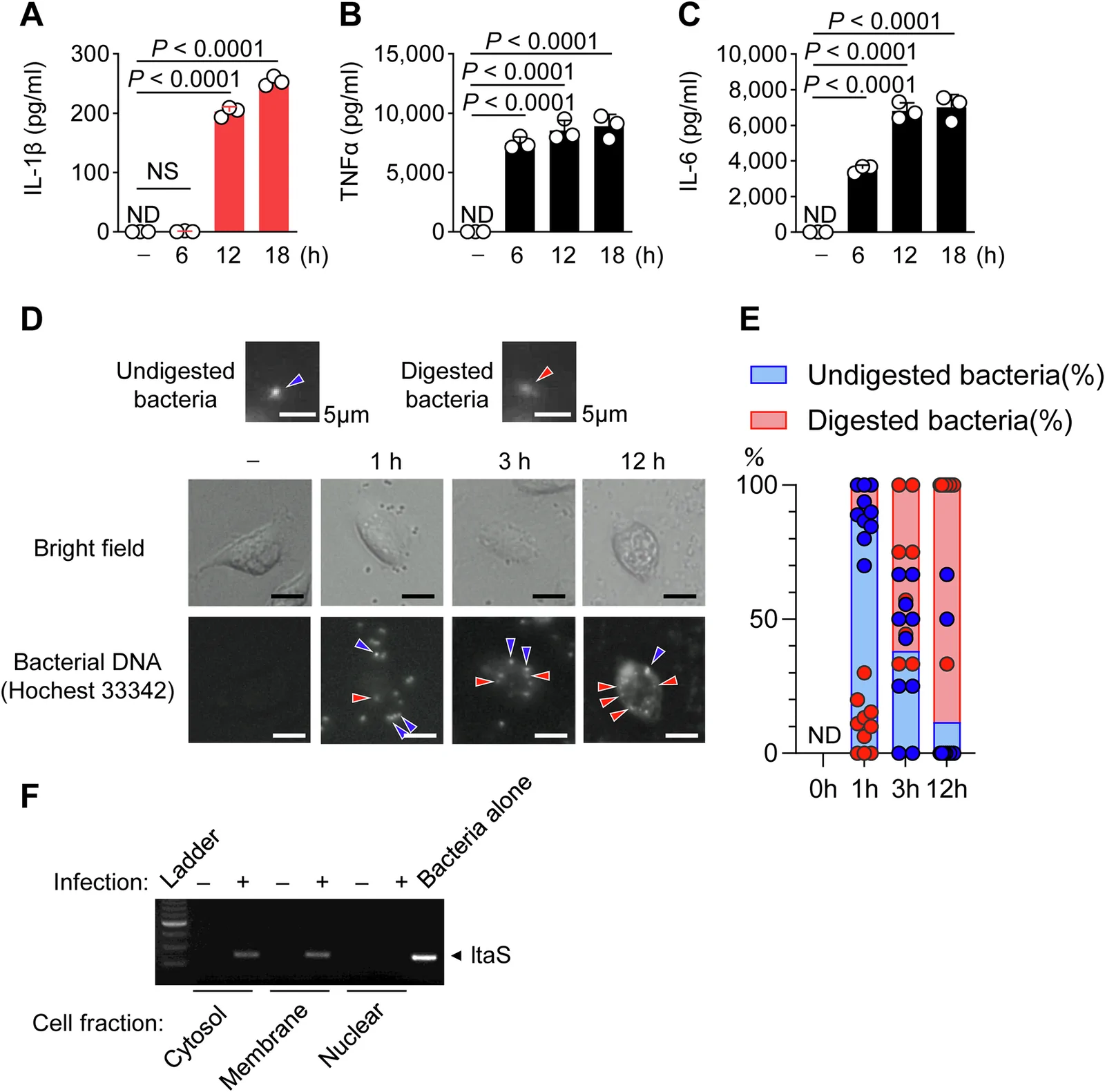
Bacterial DNA is released inside macrophages infected with *S. aureus*. (**A**‒**C**) Cytokine levels in cell supernatants of immortalized wild-type BMDMs infected with *S. aureus* 8325-4 at MOI = 50 for the indicated times. Bars represent the mean ± SD of three biological replicates. Statistical analysis was performed using one-way ANOVA followed by Dunnett’s multiple comparison test. (**A**) *P* < 0.0001, *P* < 0.0001, (**B**) *P* < 0.0001, *P* < 0.0001, *P* < 0.0001, (**C**) *P* < 0.0001, *P* < 0.0001, *P* < 0.0001. NS no significant difference. ND not detected. (**D**,** E**) Visualizing bacterial DNA using Hoechst 33342 in peritoneal macrophages infected with *S. aureus* 8325-4 at MOI = 25 for the indicated times. Blue and red arrowheads indicate undigested and digested bacterial DNA, respectively. Scale bars, 10 μm or an indicated size. Representative data is shown from ten biological replicates. ND not detected. (**F**) Amplification of *ltaS* in fractionated-peritoneal macrophages infected with *S. aureus* 8325-4 at MOI = 50 for 12 h. Source data are available online for this figure.

Several molecular mechanisms by which *S. aureus* activates the NLRP3 inflammasome have been reported (An et al, 2019; Craven et al, 2009; Munoz-Planillo et al, 2009; Wang et al, 2020). In contrast, knowledge of the molecular mechanisms underlying AIM2 activation by *S. aureus* remains limited. Because AIM2 is a sensor for intracellular DNA, we hypothesize that bacterial DNA released into the cytosol is detected by AIM2 to activate the inflammasome. To assess whether bacterial DNA is released in the cytosol of macrophages, we infected macrophages with *S. aureus* at a multiplicity of infection (MOI) of 50 for 1 h, and the infected macrophages were monitored for cytokine release over time. IL-1β release was detected 6 h post-infection, which was increased by 12 and 18 h of culture (Fig. 5A; Appendix Fig. S7A). In contrast, TNFα and IL-6, which are induced independently of the inflammasome, were produced at high levels by 6 h post-infection with *S. aureus* (Fig. 5B,C; Appendix Fig. S7B,C). Therefore, we assessed the presence of bacterial DNA using the DNA-binding dye Hoechst 33342 at different times after infection, as previously reported (Amer et al, 2005; Chiaraviglio et al, 2008; Jones et al, 2010). Because a large amount of IL-1β was detected at 12 h post-infection, we hypothesized that bacterial DNA becomes exposed before 12 h post-infection and therefore examined this at 1, 3, and 12 h post-infection. Bacterial DNA was clearly detected at 1 and 3 h post-infection, but the pattern of DNA staining was less clear at 12 h, suggesting that bacterial DNA was further degraded or diffused inside host cells (Fig. 5D,E). To further assess the presence of bacterial DNA inside host cells, we prepared cytosol, membrane, and nuclear fractions and amplified a *ltaS* DNA fragment that is a specific gene of Gram-positive bacteria by polymerase chain reaction. The amplicon of *ltaS* fragment was detected in the cytoplasm and membrane fractions, but not in the nuclear fraction of macrophages infected with *S. aureus* for 12 h (Fig. 5F). These results indicate that bacterial DNA from *S. aureus* is released into the macrophage cytosol after *S. aureus* infection, which is sensed by AIM2 to induce inflammasome activation.

### Deletion of AIM2 improves *S. aureus* infection outcome through IL-1β and IL-18 reduction

**Figure 6:**
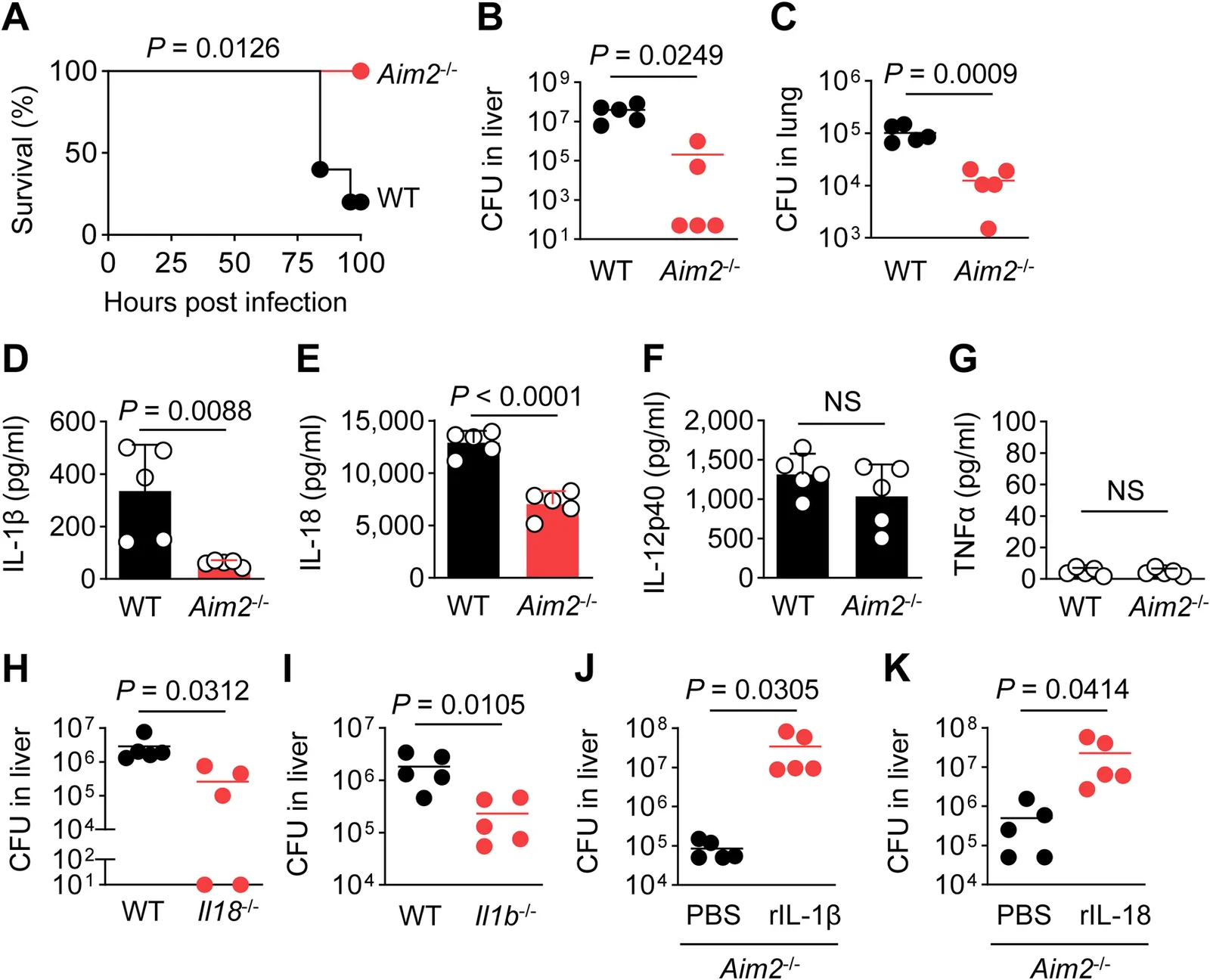
AIM2 exacerbates *S. aureus* infection through IL-1β and IL-18 production. (**A**) Mouse survival after intravenous infection with 1 × 10^8^ CFU of *S. aureus* NUMR101. Representative data are shown from five biological replicates. Survival analysis was performed using the Log-rank test. *P* = 0.0126. (**B**,** C**) CFU in the indicated organs and (**D**‒**G**) cytokine levels in the sera on day 2 after infection intravenously with 2 × 10^8^ CFU of *S. aureus* NUMR101. Representative data (**B**,** C**, means; **D**‒**G**, mean ± SD) are shown from five biological replicates. Statistical analysis was performed using a two-tailed *t*-test. (**B**) *P* = 0.0249, (**C**) *P* = 0.0009, (**D**) *P* = 0.0088, (**E**) *P* < 0.0001. NS no significant difference. (**H**‒**K**) CFU in the indicated organs on day 2 after infection intravenously with 2 × 10^8^ CFU of *S. aureus* NUMR101. (**J**,** K**) Recombinant mouse IL-18 and IL-1β were administrated by intraperitoneal injection (1 μg/mouse) on 3 and 24 h after infection. Representative data (means) are shown from five biological replicates. Statistical analysis was performed using a one-tailed *t*-test. (**H**) *P* = 0.0312, (**I**) *P* = 0.0105, (**J**) *P* = 0.0305, (**K**) *P* = 0.0414. Source data are available online for this figure.

**Figure EV3:**
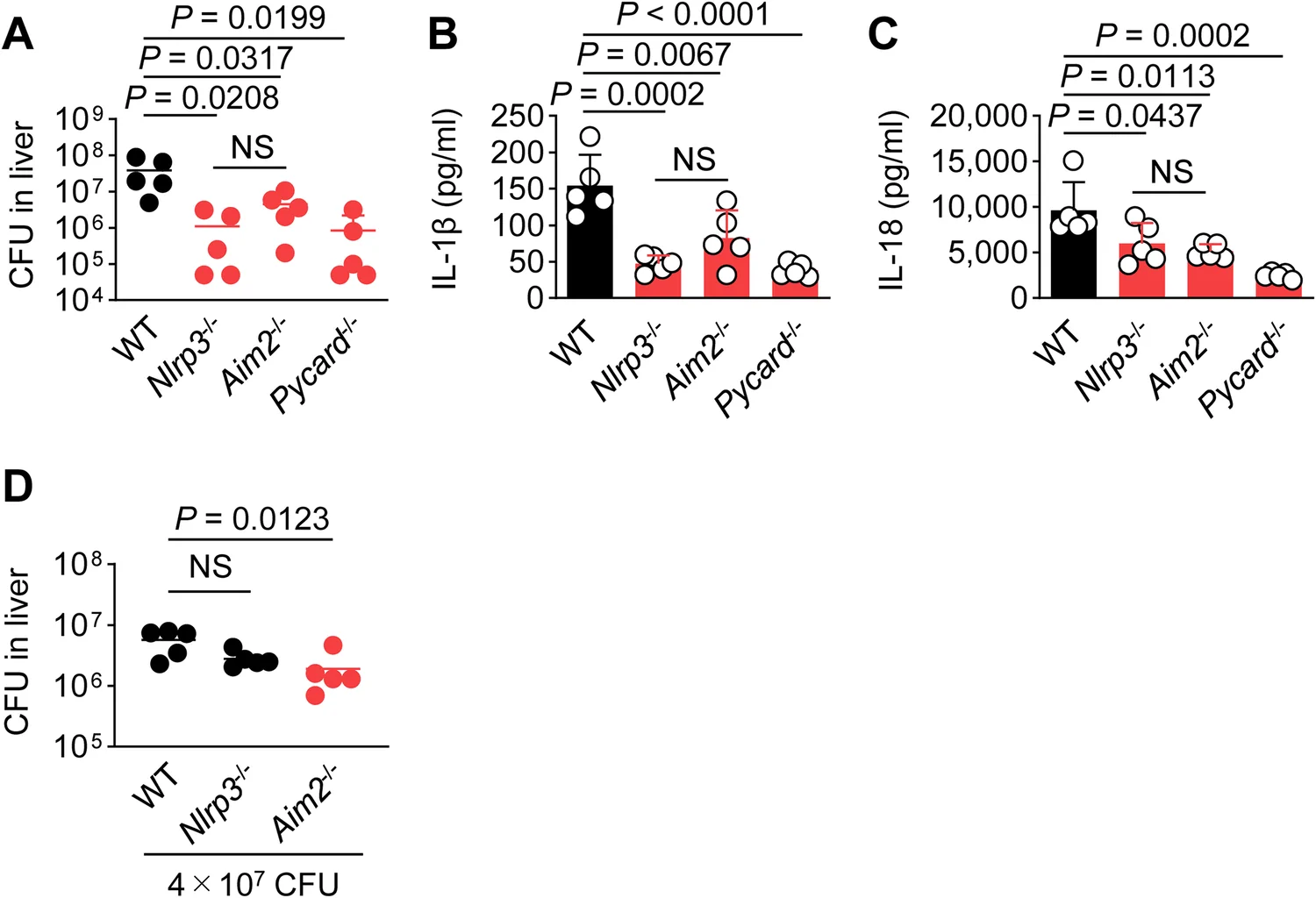
Both NLRP3 and AIM2 inflammasome activations exacerbate *S. aureus* infection. (**A**) CFU in the indicated organs on day 2 after infection intravenously with 1 × 10^8^ CFU of *S. aureus* NUMR101. Representative data (mean) are shown from five biological replicates. Statistical analysis was performed using one-way ANOVA followed by Dunnett’s multiple comparison test. (**A**) *P* = 0.0208, *P* = 0.0319, *P* = 0.0199. (**B**,** C**) Cytokine levels in the sera on day 2 after intravenous infection with 1 ×  10^8^ CFU of *S. aureus* NUMR101. Representative data (mean ± SD) are shown from five biological replicates. Statistical analysis was performed using one-way ANOVA followed by Dunnett’s multiple comparison test. (**B**) *P* = 0.0002, *P* = 0.0067, *P* < 0.0001, (**C**) *P* = 0.0437, *P* = 0.0113, *P* = 0.0002. NS, no significant difference. (**D**) CFU in the indicated organs on day 2 after infection intravenously with 4 × 10^7^ CFU of *S. aureus* NUMR101. Representative data (mean) are shown from five biological replicates. Statistical analysis was performed using one-way ANOVA followed by Dunnett’s multiple comparison test. (**D**) *P* = 0.0123. NS no significant difference.

Next, we examined the contribution of AIM2 inflammasome to the severity of bacterial infection in vivo. Eighty percent of wild-type mice succumbed to infection with 1 × 10^8^ colony forming unit (CFU) of *S. aureus* intravenously, whereas all *Aim2*^−/−^ mice survived the infection within 100 h post-infection (Fig. 6A). Bacterial burdens of *S. aureus* in the liver and the lung of *Aim2*^−^^/−^ mice were reduced on day 2 compared with that of wild-type mice (Fig. 6B,C). *S. aureus* induced the production of various cytokines, including IL-1β, IL-18, IL-12, and TNFα, on day 2 post-infection (Fig. 6D‒G). Notably, IL-1β and IL-18, but not IL-12 or TNFα production was greatly reduced in the serum of infected *Aim2*^−/−^ mice when compared with wild-type mice (Fig. 6D‒G). To assess whether endogenous IL-1β or IL-18 regulates the clearance of *S. aureus*, we infected wild-type, *Il1b*^−/−^, and *Il18*^−/−^ mice with *S. aureus* and measured the pathogen burdens. Bacterial clearance was improved both in *Il1b*^*−/−*^ and *Il18*^*−/−*^ mice (Fig. 6H,I), suggesting that these cytokines are responsible for exacerbated infection. To confirm that an increase in IL-1β and IL-18 exacerbates *S. aureus* infection, recombinant IL-1β or IL-18 was administered to *Aim2*^−/−^ mice. Consistent with the findings in mutant mice, administration of either cytokine significantly increased the bacterial loads in infected mice (Fig. 6J,K). As reported in previous studies (Cohen et al, 2018; Kebaier et al, 2012), mice deficient for NLRP3 showed less bacterial burden in the livers and production of IL-1β and IL-18 after infection with *S. aureus*, with no significant differences between *Nlrp3*^-/-^ and *Aim2*^-/-^ mice (Fig. EV3A–C). In *Pycard*^-/-^ mice, inflammasome responses were more clearly reduced than in NLRP3 or AIM2-deficient mice (Fig. EV3B,C). In contrast, when mice were infected with a lower bacterial dose, the predominant contribution of AIM2 was observed (Fig. EV3D). These results indicate that NLRP3- and AIM2-mediated inflammasome responses cooperatively exacerbate the pathogenesis of *S. aureus* infection.

### Inflammasome suppression by JNK-Aurora-ASC improves antimicrobial-resistant *S. aureus* infection

**Figure 7:**
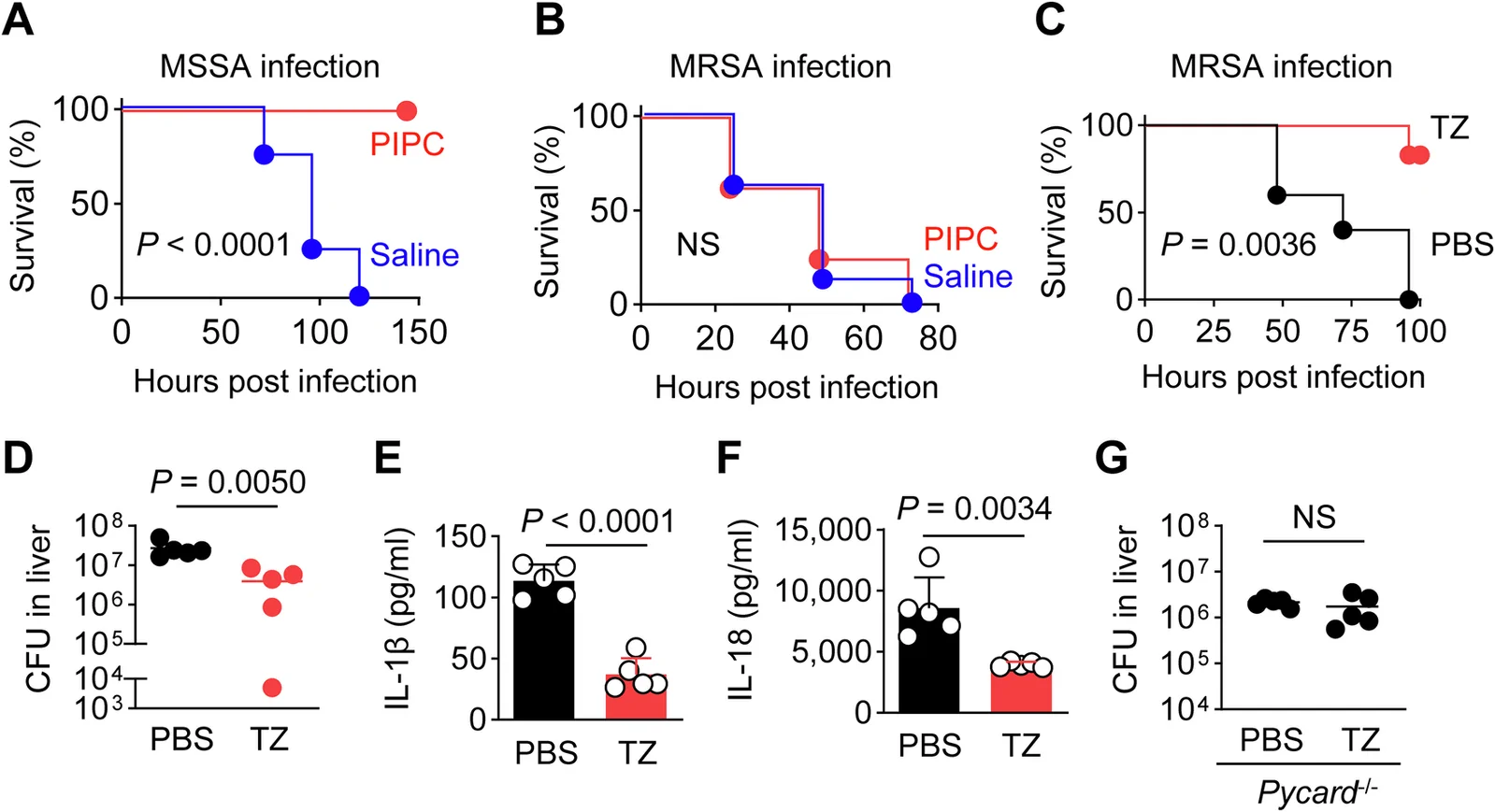
Inhibition of Aurora A reduces IL-1β and IL-18, and ameliorates *S. aureus* infection. (**A**,** B**) Mouse survival after intravenous infection with 2 × 10^8^ CFU of (**A**) MSSA NUMS001 strain and (**B**) MRSA NUMR101 strain. Mice were intraperitoneally treated with piperacillin at 100 mg/kg/day or saline. Representative data are shown from eight biological replicates. Survival analysis was performed using the Log-rank test. *P* < 0.0001. NS no significant difference. (**C**) Mouse survival after intravenous infection with 2 × 10^8^ CFU of *S. aureus* NUMR101. Representative data are shown from five biological replicates. Survival analysis was performed using the Log-rank test. *P* = 0.0036. (**D**,** G**) CFU in the indicated organs and (**E**,** F**) cytokine levels in the sera on day 2 after infection intravenously with 2 × 10^8^ CFU of *S. aureus* NUMR101. Tozasertib (TZ) was administered by intraperitoneal injection (1 mg/mouse) on 3 and 24 h after infection. Representative data (**D**,** G**, means; **E**,** F**, mean ± SD) are shown from five biological replicates. Statistical analysis was performed using a two-tailed *t*-test. (**D**) *P* = 0.0050, (**E**) *P* < 0.0001, (**F**) *P* = 0.0034. NS no significant difference. Source data are available online for this figure.

**Figure EV4:**
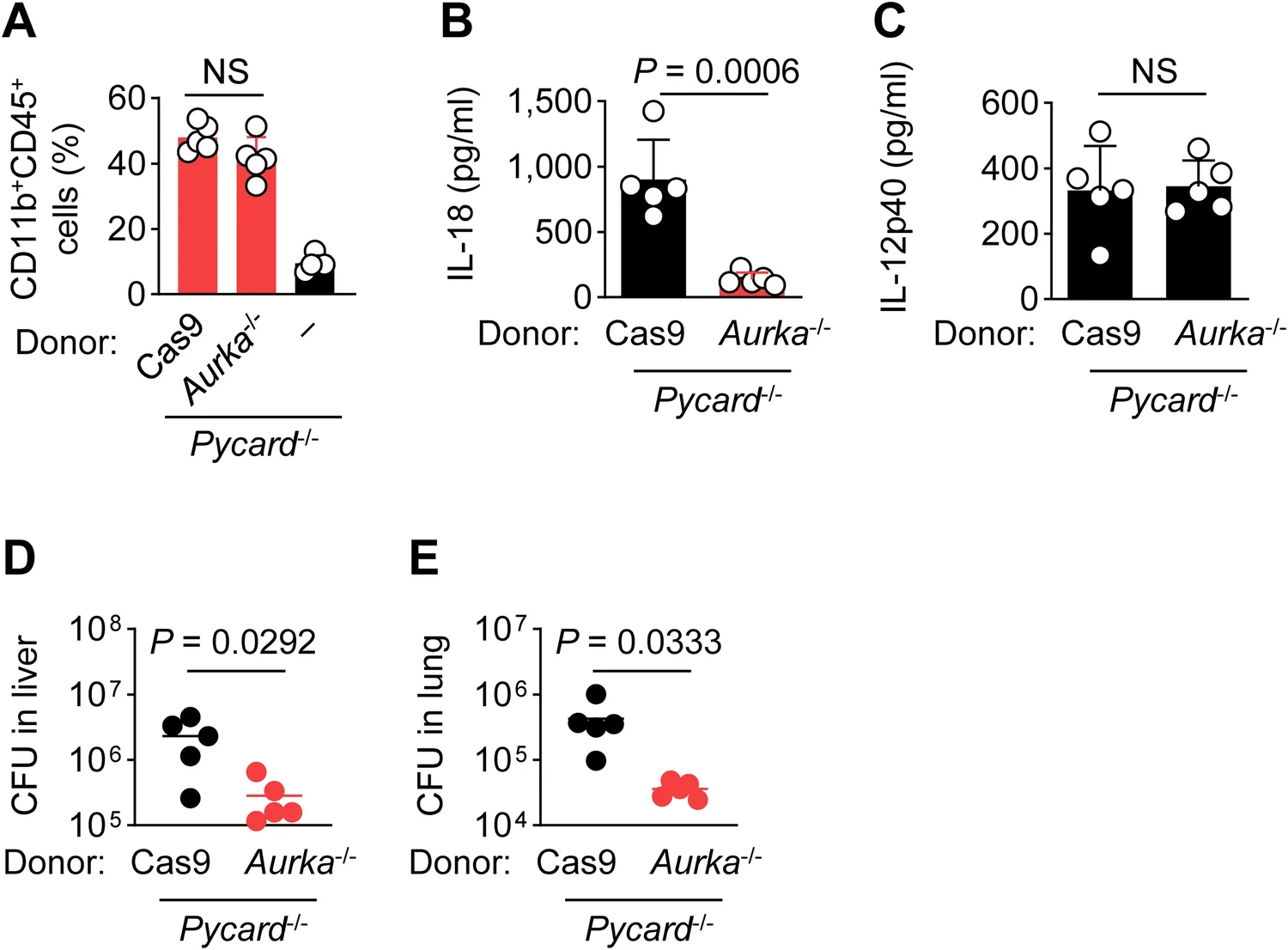
Adoptive transfer of *Aurka*^−/−^ BMDMs reduces the bacterial burden in mice infected with *S. aureus.* (**A**) Percentage of CD11b^+^CD45^+^ cells in the lung with or without transplantation of immortalized Cas9 or *Aurka*^−/−^ BMDMs into *Pycard*^−/−^ mice. Representative data (mean ± SD) are shown from five biological replicates. Statistical analysis was performed using one-way ANOVA followed by Tukey’s multiple comparison test. NS, no significant difference. (**B**,** C**) Cytokine levels in the sera and (**D**,** E**) CFU in the indicated organs on day 2 after intravenous infection with 4 × 10^7^ CFU of *S. aureus* NUMR101, followed by macrophage transplantation (Donor). Representative data (**B**,** C**, mean ± SD; **D**,** E**, mean) are shown from five biological replicates. Statistical analysis was performed using a two-tailed t-test. (**B**) *P* = 0.0006, (**D**) *P* = 0.0292, (**E**) *P* = 0.0333. NS no significant difference.

**Figure EV5:**
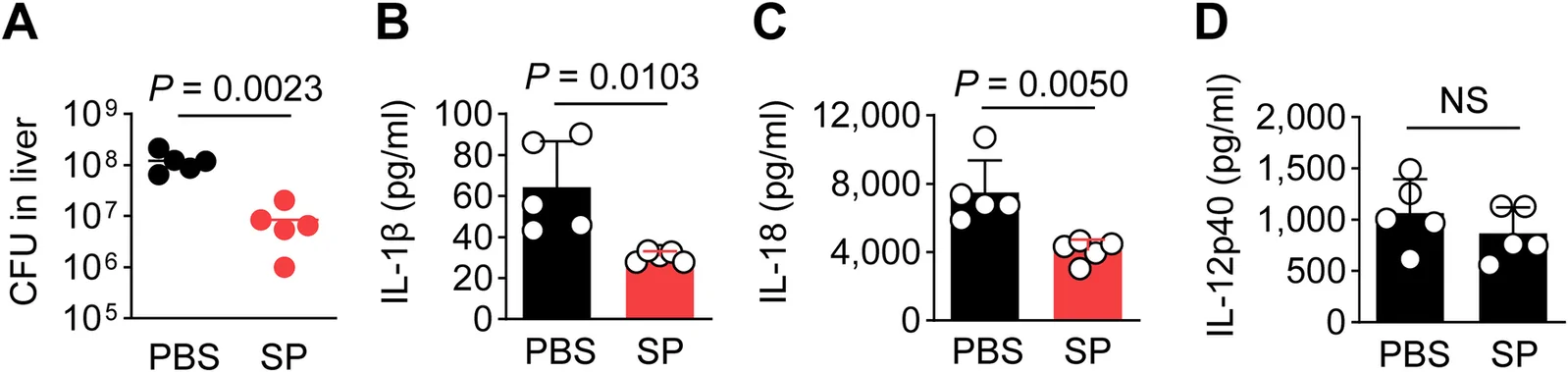
JNK inhibitor ameliorates *S. aureus* infection in vivo. (**A**) CFU in the indicated organs and (**B**‒**D**) cytokine levels in the sera on day 2 after infection intravenously with 2 × 10^8^ CFU of *S. aureus* NUMR101. SP600125, a JNK inhibitor, was administered by intraperitoneal injection (1 mg/mouse) on 3 and 24 h after infection. Representative data (**A**, mean; **B**‒**D**, mean ± SD or mean) are shown from five biological replicates. Statistical analysis was performed using a two-tailed *t*-test. (**A**) *P* = 0.0023, (**B**) *P* = 0.0103, (**C**) *P* = 0.0050. NS no significant difference.

Because the JNK-Aurora-ASC signal was indispensable for inflammasome activation after *S. aureus* infection (Figs. 1–3), we next evaluated the effect of inhibiting the signaling pathway in vivo. About 2 ×  10^8^ CFU of methicillin-sensitive *S. aureus* NUMS001 (MSSA) or NUMR101 (MRSA) were intravenously infected into the wild-type mice. The mice infected with MSSA were rescued by administration of piperacillin, a semisynthetic penicillin with a wide spectrum of antimicrobial activity (Fig. 7A; Appendix Fig. S8). However, all mice succumbed to an infection with 10^8^ CFU of antimicrobial-resistant *S. aureus* NUMR101 regardless of piperacillin administration (Fig. 7B). Notably, 80% of Tozasertib-treated mice survived after MRSA infection, which was untreatable with piperacillin, whereas all control mice succumbed (Fig. 7C). Bacterial burden in the liver was reduced about tenfold on day 2 by Tozasertib administration (Fig. 7D). Consistent with these results, the amounts of IL-1β and IL-18 were reduced in the serum of mice treated with Tozasertib compared to control mice (Fig. 7E,F). In contrast, Tozasertib administration did not reduce bacterial burdens in the livers of *Pycard*^−/−^ mice after MRSA infection (Fig. 7G), indicating that Aurora signaling increases bacterial burdens in MRSA-infected mice in an ASC-dependent manner. Furthermore, since *Aurka*^−/−^ mice are embryonically lethal (Lu et al, 2008), we transplanted immortalized *Aurka*^−/−^ macrophages into *Pycard*^−/−^ mice to assess the contribution of Aurora-mediated inflammasome responses, as previously reported (Fig. EV4A) (Wang et al, 2023; Zhang et al, 2024; Zhang et al, 2025). Compared with mice transplanted with Cas9 control cells, mice transplanted with *Aurka*^−^^/−^ macrophages showed reduced IL-1β production and MRSA bacterial burdens (Fig. EV4B‒E). A similar effect was observed in mice administered SP600125, an inhibitor of JNK. Bacterial burden in the liver was reduced in SP600125-treated mice (Fig. EV5A). Also, IL-1β and IL-18, but not IL-12p40, were reduced in SP600125-treated mice (Fig. EV5B‒D). These results suggest that MRSA infection can be controlled independently of antibiotics by blocking the JNK-Aurora-ASC axis through the inhibition of inflammasome responses.

## Discussion

Inflammasome activation can act as a double-edged sword in host defense. While inflammasomes promote early pathogen clearance, excessive activation can worsen inflammatory pathology, especially in infections caused by Gram-positive bacteria such as *S. aureus*, *Listeria monocytogenes*, *Bacillus cereus*, *Clostridium perfringens*, and *Mycobacterium tuberculosis* (Ghimire et al, 2018; Hara et al, 2018; Krause et al, 2019; Mathur et al, 2019; Paudel et al, 2019; Subbarao et al, 2020; Yamamura et al, 2019). Moreover, hyperactivation of inflammasomes contributes to autoinflammatory diseases, autoimmune disorders, and cytokine storm syndromes (Chou et al, 2021; Martinon and Tschopp, 2004). Understanding the molecular mechanisms that fine-tune inflammasome activation is therefore crucial for developing therapeutic interventions.

In this study, we reveal that Aurora kinases play a key role in regulating inflammasome activation during *S. aureus* infection. Although Aurora kinase A, B, and C have been extensively studied in cell cycle regulation and targeted in cancer therapy (Naso et al, 2021; Vats et al, 2025), their involvement in host innate immunity has remained poorly understood. Our findings significantly expand the functional repertoire of Aurora A, showing that it phosphorylates ASC on S100 and S105 to promote inflammasome assembly. In contrast to ASC S100, Aurora kinase was not predicted as a candidate for ASC S105. Based on previous reports (Ferrari et al, 2005), it is possible that KinasePhos 3.0 was programmed to preferentially recognize Aurora A substrates in which a hydrophobic amino acid is located immediately C-terminal to the serine residue, which may explain why S100 was preferentially identified. Using these predictions as a reference, we performed functional analyses in macrophages and were thereby able to identify ASC S105 as an additional functionally relevant site. As shown in Appendix Fig. S9, we examined the conservation of the Aurora A target residues ASC S100 and S105 across nine animal species and found that each serine residue was conserved in ~60% of the species. Moreover, this alignment shows that serine and threonine residues that can serve as phosphorylation targets are clustered within this region of ASC, raising the possibility that Aurora A may also phosphorylate other amino acid residues in a species-dependent manner. This serine phosphorylation is functionally distinct from the previously reported Y144 tyrosine phosphorylation by spleen tyrosine kinase (Chung et al, 2016; Hara et al, 2013), suggesting that ASC acts as a signaling hub integrating multiple phosphorylation events from diverse kinases. In addition to Aurora A, Aurora B and C may also have similar immunological functions. In fibroblasts and ovarian granulosa cells, JNK has been reported to promote entry into mitosis through phosphorylation of Aurora B (Oktay et al, 2008), however, the immunological functions of Aurora kinases in macrophages have remained unclear. Our results indicate that JNK1 directly phosphorylates Aurora A to regulate immune responses in macrophages, and *S. aureus* has been reported to activate JNK via TLR2 (Fang et al, 2014; Watanabe et al, 2007). ASC phosphorylation through JNK is required for the translocation of ASC to form ASC speck (Hara et al, 2013). Because JNK has been reported to promote inflammasome activation via ASC in the context of other pattern-recognition receptors (Feng et al, 2018; Hara et al, 2013; Jiang et al, 2020; Li et al, 2015; Okada et al, 2014), it is possible that Aurora A is also activated in response to other triggers in addition to *S. aureus* infection. Even in *Mapk8*^−/−^ macrophages, Aurora A was slightly activated during *S. aureus* infection, suggesting that activation of Aurora A is regulated by kinase(s) other than JNK during pathogen infection.

An implication of our findings is that *S. aureus* activates multiple inflammasomes, including AIM2 and NLRP3, through distinct pathogen-associated molecular patterns (PAMPs), such as cytosolic bacterial DNA and hemolysins. In addition to NLRP3 and AIM2, NLRP6 and NLRC4 inflammasomes have also been reported to exacerbate the pathogenesis of *S. aureus* infection. (Ghimire et al, 2018; Hara et al, 2018; Paudel et al, 2019). This combinatorial inflammasome activation likely explains why inhibition of a single inflammasome sensor (e.g., NLRP3) has limited effectiveness during complex bacterial infections (Ghimire et al, 2018; Hara et al, 2018; Sokolovska et al, 2013). By contrast, targeting ASC or the upstream signaling components that control its phosphorylation offers a mechanistically grounded strategy for broad inflammasome suppression. Our results also highlight a previously underappreciated role of AIM2 in mediating pathological inflammation during *S. aureus* infection. Although *S. aureus* has long been considered an extracellular pathogen, accumulating evidence indicates that it can survive and replicate within macrophages (Bravo-Santano et al, 2018; Neumann et al, 2016). The detection of cytosolic bacterial DNA and the requirement of AIM2 for IL-1β and IL-18 secretion support this intracellular phase of infection. Furthermore, genetic deletion of AIM2 or neutralization of IL-1β/IL-18 markedly reduced bacterial burden and improved survival, reinforcing the detrimental impact of excessive inflammasome-driven cytokines.

From a translational perspective, our most striking finding is that pharmacological inhibition of Aurora A (or upstream JNK) protects mice from lethal MRSA infection, even when standard antibiotics such as piperacillin are ineffective. Given that multiple Aurora kinase inhibitors, including Alisertib, EP0042, and JAB-2485, are already in clinical trials or have FDA orphan drug designation, repurposing these inhibitors for infectious disease treatment could be feasible and rapidly implementable. Aurora kinase inhibitors are unlikely to replace antibiotics but could serve as adjunctive immunomodulatory therapies to reduce inflammation-induced tissue damage and enhance host-pathogen clearance. Such strategies may be particularly valuable for highly resistant pathogens or for infections where inflammasome hyperactivation drives pathology, as seen in severe bacterial sepsis, MRSA pneumonia, and viral infections such as COVID-19.

In conclusion, our findings identify the JNK-Aurora-ASC axis as a central regulatory pathway controlling inflammasome activation in *S. aureus* infection. Targeting this pathway represents a potential alternative therapeutic avenue for combating antimicrobial-resistant infections and may have broader applications in diverse inflammatory diseases driven by excessive inflammasome activation. On the other hand, because Aurora kinases have broad cellular effects, including roles in cell cycle regulation, the improved survival observed in vivo may also include indirect effects unrelated to inflammasome regulation. To avoid effects on other cell types, it may be necessary to develop strategies to deliver Aurora inhibitors to phagocytic cells such as macrophages.

## Methods

**Table:** Reagents and tools table

Reagent/resource	Reference or source	Identifier or catalog number
**Experimental models**		
ASC wild-type 3xFLAG-mRuby3 reconstituted immortalized BMDMs	This study	N/A
ASC S16A 3xFLAG-mRuby3 reconstituted immortalized BMDMs	This study	N/A
ASC S92A 3xFLAG-mRuby3 reconstituted immortalized BMDMs	This study	N/A
ASC S100A 3xFLAG-mRuby3 reconstituted immortalized BMDMs	This study	N/A
ASC S105A 3xFLAG-mRuby3 reconstituted immortalized BMDMs	This study	N/A
ASC S100/105A-3xFLAG-mRuby3 reconstituted immortalized BMDMs	This study	N/A
Wild-type immortalized BMDMs	Tanishita et al, 2022	N/A
Mapk8 KO immortalized BMDMs	This study	N/A
ASC wild-type SFP reconstituted immortalized BMDMs	Hara et al, 2018	N/A
ASC Y144F-SFP reconstituted immortalized BMDMs	Tanishita et al, 2022	N/A
Cas9 knockin immortalized BMDMs	Tanishita et al, 2022	N/A
ASC KO immortalized BMDMs	Hara et al, 2018	N/A
L929 cells	Tanishita et al, 2022	N/A
HEK293T cells	Tanishita et al, 2022	N/A
C57BL/6 wild-type mice	Sankyo Labo Service	https://www.sankyolabo.co.jp/
C57BL/6 wild-type mice	Tokyo Laboratory Animals Science	https://kwl-a.co.jp/
*Aim2*^−/−^ mice	Hara et al, 2018	N/A
*Nlrp3*^−/−^ mice	This study	N/A
*Pycard*^−/−^ mice	Yamamoto et al, 2004	N/A
*Il1b*^−^^/–^ mice	Horai et al, 1998	N/A
*Il18*^−/−^ mice	Takeda et al, 1998	N/A
*Staphylococcus aureus* 8325-4	Hara et al, 2018	N/A
*Staphylococcus aureus* NUMR101	Yanagihara et al, 2006	N/A
*Staphylococcus aureus* NUMS001	Yanagihara et al, 2006	N/A
**Recombinant DNA**		
pHIV-EGFP	Addgene	Cat#: 21373
lentiGuide-Puro	Addgene	Cat#: 52963
**Antibodies**		
Mouse anti-caspase-1 p20 mAb	Adipogen	Cat#: AG-20B-0042
Rabbit anti-ASC pAb	Adipogen	Cat#: AG-25B-0006
Goat anti-IL-1β pAb	R&D systems	Cat#: AB-401
Mouse anti-Aurora A mAb	Novus biologicals	Cat#: NB100-1641
Rabbit anti-Aurora A mAb	Abcam	Cat#: ab247643
Rabbit anti-Aurora A pAb	Proteintech	Cat#: 10297-1-AP
Rabbit anti-p- Aurora A (Thr288)/Aurora B (Thr232)/Aurora C (Thr198) mAb	Cell Signaling Technology	Cat#: 2914
Rabbit anti-JNK pAb	Cell Signaling Technology	Cat#: 9252
Mouse anti-p-JNK (Thr183/Tyr185) mAb	Cell Signaling Technology	Cat#: 9255
Rabbit anti-p-JNK (Thr183/Tyr185) mAb	Cell Signaling Technology	Cat#: 4668
HRP-Mouse anti-GAPDH mAb	Proteintech	Cat#: HRP-60004
Mouse anti-β-actin mAb	GeneTex	Cat#: GTX629630
Mouse anti-FLAG mAb	Sigma-Aldrich	Cat#: F1804
Mouse anti-pSer mAb	Santa Cruz Biotechnology	Cat#: sc-81514
Rat anti-ASC pAb	He et al, 2016	N/A
Mouse anti-CDC25B mAb	Proteintech	Cat#: 67145-1-Ig
Rabbit anti-p-CDC25B (Ser353) pAb	Thermo Fisher Scientific	Cat#: PA5-104865
Alexa Fluor 488-goat anti-rat IgG Ab	Thermo Fisher Scientific	Cat#: A11006
TruStain FcX	BioLegend	Cat#: 101320
PerCP/Cyanine5.5 anti-mouse CD45 Ab	BioLegend	Cat#: 103132
FITC-anti-CD11b mAb	Thermo Fisher Scientific	Cat#: 11-0112-82
ADP-Glo Kinase Assay kit	Promega	Cat#: V6930
**Oligonucleotides and other sequence-based reagents**		
Aurora A gRNA	FASMAC	CAGTCACCAAGACGCGTTT
ltaS Fw primer	Eurofins	TATTAACCGTAATAACGATTACCTTGAAGAC
ltaS Rv primer	Eurofins	ATCAGAGAAGAATCTAAAGTACACAACATTG
**Chemicals, Enzymes and other reagents**		
Mouse IL-1β ELISA kit	Thermo Fisher Scientific	Cat#: 88-7013-88
Mouse IL-18 ELISA kit	Thermo Fisher Scientific	Cat#: 88-50618-77
Mouse IL-12p40 ELISA kit	Thermo Fisher Scientific	Cat#: 88-7120-88
Mouse IL-6 ELISA kit	Thermo Fisher Scientific	Cat#: 88-7064-88
Mouse TNFα ELISA kit	Thermo Fisher Scientific	Cat#: 88-7324-88
Lipofectamine LTX with PLUS	Thermo Fisher Scientific	Cat#: 15338100
Poly(dA:dT)	Invivogen	Cat#: tlrl-3pelps
RPMI 1640	Nacalai Tesque	Cat#: 30264-85
Non-essential amino acids	Nacalai Tesque	Cat#: 06344-56
Sodium pyruvate	Nacalai Tesque	Cat#: 06977-34
Protease inhibitor cocktail	Nacalai Tesque	Cat#: 25955-24
Phosphatase inhibitor cocktail	Nacalai Tesque	Cat#: 07575-51
Dimethyl sulfoxide	Nacalai Tesque	Cat#: 08904-14
Agar	Nacalai Tesque	Cat#: 01028-85
Antibiotic-Antimycotic Mixed Stock Solution	Nacalai Tesque	Cat#: 09366-44
Blocking One	Nacalai Tesque	Cat#: 03953-95
Blocking One-P	Nacalai Tesque	Cat#: 05999-84
Chemi-Lumi One	Nacalai Tesque	Cat#: 07880
Disuccinimidyl suberate	Tokyo chemical industry	Cat#: D3895
ProLong Glass Antifade	Thermo Fisher Scientific	Cat#: P36980
Opti-MEM	Thermo Fisher Scientific	Cat#: 32985070
LPS	Invivogen	Cat#: tlrl-3pelps
Nigericin	Invivogen	Cat#: tlrl-nig
Recombinant mouse IL-18	MBL	Cat#: B002-5
Recombinant mouse IL-1b	BioLegend	Cat#: 575102
Protein G resin	Genscript	Cat#: L00209
Gentamicin solution	FUJIFILM Wako Pure Chemical	Cat#: 078-06061
LB broth	Kanto Chemical	Cat#: 24308-08
DAPI	Sigma-Aldrich	Cat#: D9542
Recombinant JNK1 protein	Sigma-Aldrich	Cat#: J2455
Recombinant Aurora A protein	Sino Biological	Cat#: A28-10G
SP600125	Sigma-Aldrich	Cat#: 420119
FR180204	Sigma-Aldrich	Cat#: 328010
SB203580	Sigma-Aldrich	Cat#: 559389
R406	Sigma-Aldrich	Cat#: SML4053
Alisertib	Selleck Biotech	Cat#: S1133
Tozasertib	Selleck Biotech	Cat#: S1048
H 89 2HCl	Selleck Biotech	Cat#: S1582
Volasertib	Selleck Biotech	Cat#: S2235
Silmitasertib	Selleck Biotech	Cat#: S2248
TAK-659	Selleck Biotech	Cat#: S8442
PRT062607	Selleck Biotech	Cat#: S8032
PP2	Selleck Biotech	Cat#: S7008
OSU-03012	Selleck Biotech	Cat#: S1106
Dinaciclib	Selleck Biotech	Cat#: S2768
Sotrastaurin	Selleck Biotech	Cat#: S2791
Hoechst 33342	Thermo Fisher Scientific	Cat#: H3570
Piperacillin	FUJIFILM Toyama Chemical	Cat#: 473600455
ADP-Glo Kinase assay kit	Promega	Cat#: V6930
NE-PER Nuclear and Cytoplasmic Extraction Reagents	Thermo Fisher Scientific	Cat#: 78833
Mouse Aurora A	CycLec	Cat#: CY-SPA28-M
S‧protein agarose	Novagen	Cat#: 69704
Anti-FLAG affinity gel	Sigma-Aldrich	Cat#: A2220
Duolink in situ PLA probe anti-rabbit PLUS	Sigma-Aldrich	Cat#: DUO92002
Duolink in situ PLA probe anti-mouse MINUS	Sigma-Aldrich	Cat#: DUO92004
Duolink in situ PLA Detection Reagents Red	Sigma-Aldrich	Cat#: DUO92008
RBC Lysis Buffer	Thermo Fisher Scientific	Cat#: 00-4300-54
Fixable Viability Dye eFluor 780	Thermo Fisher Scientific	Cat#: 65-0865-14
**Software**		
KinasePhos 3.0	Ma et al, 2023	https://awi.cuhk.edu.cn/KinasePhos/download.html
BZ-X800 viewer	Keyence	https://www.keyence.co.jp/
LAS X Office 1.4.4	Leica	https://www.leica-microsystems.com/jp/
FlowJo software v10	FlowJo LLC	https://flowjo.com/flowjo/overview
Hybrid Cell Count software	Keyence	Cat#: BZ-H4C
**Other**		
PVDF membrane	Millipore	Cat#: IPVH00010
FBS	SAFC biosciences	Cat#: 12003 C
Lab-Tek Chamber Slides	Nunc	Cat#: 178599
Disuccinimidyl suberate	Tokyo Chemical Industry	Cat#: D3895

### Mice

*Aim2*^−/−^ (Hara et al, 2018), *Il1b*^−/−^ (Horai et al, 1998), *Il18*^−/−^ (Takeda et al, 1998), and *Pycard*^−/−^ (*Asc*^−/−^) (Yamamoto et al, 2004) mice on the C57BL/6 background have been reported. *Nlrp3*^−/−^ mice on a background of C57BL/6J were generated by CRISPR/Cas9-based genome editing at the Laboratory Animal Resource Center, University of Tsukuba (Tsukuba, Japan). Genome editing in fertilized mouse eggs was performed using methods reported previously (Mizuno-Iijima et al, 2021). C57BL/6 wild-type were purchased from Sankyo Labo Service or Tokyo Laboratory Animals Science. In accordance with the animal facility guidelines, mice were housed with bedding material, standard chow, fresh water, and an enrichment item. All animal studies were approved by the Institutional Animal Research Committee and Ethics Committee of Keio University (Protocol #08004), Nagasaki University (Protocol #1812171495-4), or Asahikawa Medical University (Protocol # R8-024).

### Bacteria

*Staphylococcus aureus* 8325-4, NUMR101, or NUMS001 (Hara et al, 2018; Yanagihara et al, 2006) were prepared as shown in previous studies (Hara et al, 2007; Hara et al, 2008). Briefly, *S. aureus* was grown in L-Broth medium at 37 °C with shaking, washed with phosphate-buffered saline (PBS), and then suspended in PBS supplemented with 10% glycerol. The bacterial suspension was stored at −80 °C in aliquots.

### Reagents

LPS, nigericin, and Poly(dA:dT) were from Invivogen, Alisertib, Tozasertib, H 89 2HCl, Volasertib, Silmitasertib, TAK-659, PRT062607, PP2, OSU-03012, Dinaciclib, and Sotrastaurin were from Selleck Biotech, Lipofectamine LTX and Hoechst 33342 from Thermo Fisher Scientific, DAPI, SP600125, FR180204, R406, SB203580, U0126, Duolink anti-Rabbit PLUS, anti-Mouse MINUS, and Detection Reagents Red from Sigma-Aldrich, Piperacillin from FUJIFILM Toyama Chemical. Antibodies for caspase-1 and ASC were from Adipogen, IL-1β from R&D Systems, Aurora A from Novus, p-Aurora A/Aurora B/Aurora C, JNK, and p-JNK from Cell Signaling Technology, Aurora A from Abcam, CDC25B, Aurora A and GAPDH from Proteintech, β-actin from GeneTex, p-Ser from Santa Cruz Biotechnology, and FLAG from Sigma-Aldrich. Rat antibodies against ASC were established in our laboratory.

### Cell culture

BMDMs were generated by differentiating bone marrow progenitors from the tibia and femur for 5 days in RPMI 1640 (Nacalai Tesque) supplemented with 10% FBS, 30% L929 cell supernatant, non-essential amino acids, sodium pyruvate and antibiotics (penicillin, streptomycin and amphotericin B). BMDMs were cultured with J2 virus to generate immortalized BMDMs (Blasi et al, 1985). BMDMs from *Nlrp3*^−/−^, *Aim2*^−/−^, and *Mapk8*^−/−^ mice have been reported (Hara et al, 2018; Hara et al, 2013). Immortalized BMDMs and L929 cells were cultured in RPMI 1640 supplemented with 10% FBS, non-essential amino acids, sodium pyruvate and gentamicin. Murine primary peritoneal macrophages were obtained 4 days after an intraperitoneal injection of 3 ml of thioglycolate medium. After washing with RPMI 1640, the cells were centrifuged, resuspended in culture medium consisting of RPMI 1640 supplemented with 10% FBS and gentamicin (10 μg/ml), and incubated on culture plates at 37 °C overnight in a 5% CO_2_ incubator. Non-adherent cells were then washed out, and adherent cells (more than 95% of cells were F4/80-positive macrophages) were used for experiments. Cell lines were routinely tested for mycoplasma contamination with published primers (Siegl et al, 2023) and were maintained in a 5% CO_2_ incubator at 37 °C.

### Targeting of Aurora A by CRISPR/Cas9 genome editing

Lentiviral CRISPR/Cas9 targeting guide RNA (gRNA) expressing vector (lentiGuide-Puro) was obtained from Addgene. The Aurora A targeting sequence used was 5’-CAGTCACCAAGACGCGTTT-3’. To generate an Aurora A mutant cell line, lentiGuide-Puro stocks containing the target sequence were used to transduce Cas9-expressing immortalized BMDMs. Seven days later, single clones of puromycin-resistant cells were isolated by limiting dilution. Deficiency of the target protein was identified by immunoblotting with a specific antibody. A mutation of the target gene was identified by DNA sequencing.

### Reconstitution of ASC mutants in *Pycard*^−/−^ macrophages

ASC-null immortalized BMDMs were transduced with lentivirus containing pHIV-ASC wild-type-, S16A-, S92A-, S100A-, S105A, or S100/105A-3xFLAG-mRuby3. After 3 days, transduced cells were sorted by flow cytometry using mRuby3 as a marker. Expression of reconstituted proteins was determined by immunoblotting. ASC wild-type, and Y144F-SFP (S-tag, FLAG, and streptavidin-binding tag)-reconstituted *Pycard*^−/−^ immortalized BMDMs were generated in previous studies (Hara et al, 2018; Tanishita et al, 2022).

### Mass spectrometry analysis

Mouse ASC-SFP was purified from HEK293T cells transduced with pcDNA3 ASC-SFP using S‧protein agarose (Novagen). Mouse Aurora A was purchased from CycLec. Total ASC-SFP was incubated with 1 μg of Aurora A in kinase buffer (Cell Signaling Technology) with ATP at 37 °C for 30 min, and digested with trypsin. Phosphorylated peptides were purified with Titansphere Phos-TiO Kit (GL Sciences) and subsequently subjected to LC-MS/MS analysis at Integrale Corporation. LC-MS/MS analysis was performed using an EASY-nLC 1200 System (Thermo Fisher Scientific), an EASY-Spray column, 15 cm × 75 µm ID, 3 µm particles, 100 Å pore size (Thermo Fisher Scientific), and a Q Exactive Plus (Thermo Fisher Scientific).

### Prediction using KinasePhos 3.0

The amino acid sequence of murine ASC was input as the phosphorylation substrate, and potential kinase candidates were identified at the kinase family level using KinasePhos 3.0 under default settings (Ma et al, 2023). The predicted kinases were ranked by score for each phosphorylation site. An overview of all predicted phosphorylation sites was presented in Fig. EV1B, and detailed information on sites S92, S100, S105, and Y144 was shown in Fig. EV1C. Data related to Aurora kinase was presented in Fig. 1C.

### Antibiotic sensitivity test

Piperacillin was diluted in brain heart infusion (BHI) medium at a concentration of 100 μg/mL, and the susceptibility to piperacillin was observed using the broth microdilution method. Bacterial growth was determined based on the formation of visible precipitates.

### Stimulation of macrophages

Cells were plated at a density of 5 × 10^5^ cells per well in 24-well microplates. Culture medium was replaced with Opti-MEM (Thermo Fisher Scientific) before infection. Cells were infected with *Staphylococcus aureus* 8325-4 or NUMR101 (MOI = 10, 25, or 50) (Hara et al, 2018; Yanagihara et al, 2006), and gentamicin (10 μg/ml) was added to the cultures 1 h after infection. As a general guideline, primary macrophages were infected with *S. aureus* at an MOI of 25, immortalized macrophages at an MOI of 50, and ASC-reconstituted macrophages at an MOI of 10. In some experiments, the MOI was adjusted as needed. Inhibitor or the solvent control dimethyl sulfoxide was added to cell cultures 30 min before infection. After infection for 18 h or the indicated times, culture supernatants were collected, centrifuged at 1500×*g* for 10 min to remove cell debris, and stored at −80 °C until cytokine measurement. Macrophages primed with LPS (50 ng/ml) for 4 h were stimulated with nigericin (10 μM) for 60 min or transfected with poly(dA:dT) (1 μg/ml) using Lipofectamine LTX for 120 min.

### Fractionation of cell lysates

Cytosol, membrane, and nuclear were fractionated using NE-PER Nuclear and Cytoplasmic Extraction Reagents (Thermo Fisher Scientific) according to the manufacturer’s instructions. About 224 bp of *ltaS* fragment was amplified using 5’- TATTAACCGTAATAACGATTACCTTGAAGAC-3’ and 5’- ATCAGAGAAGAATCTAAAGTACACAACATTG-3’.

### ELISA

The ELISA kits for mouse IL-1β, IL-18, TNFα, IL-6, and IL-12p40 were purchased from R&D Systems and Thermo Fisher Scientific. Levels of cytokines in culture supernatants were determined by two-site sandwich ELISA according to the manufacturer’s instructions.

### Immunoblotting

Cells were lysed with SDS sample buffer, and supernatants were concentrated with trichloroacetic acid. The lysates and supernatants were subjected to SDS-PAGE and subsequently transferred to PVDF membranes by electroblotting. The membrane was blocked with Blocking One (Nacalai Tesque) or Blocking One-P (Nacalai Tesque) for 2 h at room temperature. After washing with TBS with 0.1% Tween 20, the membrane was incubated with rabbit primary antibody (1:1000) overnight at 4 °C, washed, then treated with HRP-Donkey anti-rabbit IgG antibody (1:5000; Jackson Immuno Research) for 90 min at room temperature. Blots were developed using Chemi-Lumi One (Nacalai Tesque).

### Crosslinking of ASC dimers or oligomers

The Triton X-100-insoluble pellets were washed twice with PBS and then resuspended in 500 µl PBS. The resuspended pellets were crosslinked for 45 min at 37 °C with 2 mM disuccinimidyl suberate (Tokyo Chemical Industry) and then centrifuged for 15 min at 6000 × *g*. The pellets were dissolved in SDS sample buffer and subjected to SDS-PAGE.

### Immunostaining

Cells were fixed and permeabilized with 0.1% Triton X-100 in blocking buffer (2.5% BSA in PBS) for 45 min. Cells were washed with PBS and incubated for 90 min at room temperature with rat anti-ASC antibody (1:400). Cells were washed, and then incubated with anti-rat Alexa Fluor 488 antibody (Thermo Fisher Scientific, 1:400). Cells were incubated with DAPI (Sigma-Aldrich) for staining of nuclei. Approximately three hundred cells were examined in each group. After staining, cells were washed and mounted in ProLong Glass antifade (Thermo Fisher Scientific), and imaged with BZ-X810 fluorescence microscopy (Keyence). Images were acquired using a Plan Apo 40× objective lens (Nikon), BZ-X filter DAPI, BZ-X filter GFP, and BZ-X filter TRITC with Multicolor Mode and Standard Image Quality setting.

### In situ PLA

Fixed and permeabilized cells were blocked with the blocking solution supplied with Duolink in situ PLA and incubated overnight at 4 °C with the following pairs of primary antibodies: rabbit anti-p-JNK (Cell Signaling Technology) or rabbit anti-ASC (Adipogen) together with mouse Aurora A (Novus Biologicals). The cells were washed and allowed to react with a pair of proximity probes (Sigma-Aldrich). The rest of the in situ PLA procedures were performed according to the manufacturer’s instructions. The cells were imaged with BZ-X810 fluorescence microscopy (Keyence). Images were acquired using a Plan Apo 40× objective lens (Nikon), BZ-X filter DAPI-V, and BZ-X filter TRITC with Multicolor Mode and Standard Image Quality settings. Quantitative analysis was performed using Hybrid Cell Count software (Keyence) in brightness mode with a threshold of 30. The number of PLA-positive dots exceeding the threshold was quantified, and the values were normalized to those of uninfected control BMDMs to calculate fold changes.

### ADP-Glo kinase assay

Kinase activity was measured using the ADP-Glo Kinase Assay kit (Promega) according to the manufacturer’s instructions with minor modifications. Recombinant JNK and Aurora A were purchased from Sigma-Aldrich and Sino Biological, respectively. Kinase reactions were performed in kinase buffer containing 40 mM Tris-HCl (pH 7.5), 20 mM MgCl₂, and 0.1 mg/mL BSA. Reactions were carried out in white 96-well plates in a total volume of 5 μL containing JNK1 (final amount: 0, 6.25, 12.5, or 25 ng/well), Aurora A (final amount: 15 ng/well), and ATP (10 μM). Reactions were initiated by the addition of ATP and incubated at room temperature for 30 min. Following the kinase reaction, 5 μL of ADP-Glo reagent was added to each well to terminate the reaction and deplete the remaining ATP. The plate was incubated at room temperature for 40 min. Subsequently, 10 μL of kinase detection reagent was added to convert ADP to ATP and generate a luminescent signal via a luciferase reaction. The plate was incubated at room temperature for 45 min. Luminescence was measured using a microplate luminometer (integration time: 0.25 ms per well). The luminescent signal is proportional to the amount of ADP produced during the kinase reaction.

### Evaluation of bacterial digestion

*S. aureus* NUMR101 was stained with 0.1 μg/mL Hoechst 33342 in PBS for 30 min, followed by three washes with PBS. Macrophages were infected with the labeled bacteria at an MOI of 50. The infection process was monitored over time using the EVOS FL Auto Imaging System (Thermo Fisher Scientific). Images were acquired using the automated adjustment. Intracellular bacteria exhibiting distinct, well-defined contours were classified as undigested, whereas those with indistinct or blurred contours were categorized as digested (Amer et al, 2005; Chiaraviglio et al, 2008; Jones et al, 2010).

### Infection in vivo

Mice were infected intravenously with 1 or 2 ×  10^8^ CFU of *S. aureus* NUMR101 or MSSA isolated in Nagasaki University Hospital (NUMS001). To count the bacterial numbers, livers and lungs were collected on 2 days after infection, homogenized in 5 ml of PBS, serially diluted, and plated on LB agar. Levels of cytokines in the sera on 2 days after infection were determined by ELISA. Piperacillin was purchased from FUJIFILM Toyama Chemical. Mice were randomly assigned to experimental groups. The investigator who performed the CFU counting and ELISA was blinded to group allocation.

### Transplantation of macrophages

*Pycard*^−/−^ mice were infected with 4 × 10^7^ CFU of *S. aureus* NUMR101 intravenously. One hour following the infection, mice were injected intravenously with 5 × 10^6^ cells/mouse of Cas9 or *Aurka*^−/−^ immortalized BMDMs. Two days after the adaptive transplantation, organs were harvested, homogenized in 5 ml of PBS, serially diluted, and plated on LB agar. To analyze the transplanted cells, minced lung tissue was passed through a 70-µm cell strainer to prepare a single-cell suspension, followed by treatment with RBC Lysis Buffer (Thermo Fisher Scientific). To block non-specific binding, the cells were incubated with TruStain FcX (BioLegend). Subsequently, the cells were stained with a mixture of antibodies and a dead cell stain, including Fixable Viability Dye eFluor 780 (Thermo Fisher Scientific), anti-CD45 (BioLegend), and anti-CD11b (Thermo Fisher Scientific) for 30 min at room temperature. Flow cytometry was performed using an SH800 Cell Sorter (Sony Biotechnology) equipped with 405-, 488-, 561-, and 640-nm lasers and a 100-µm sorting chip under standard pressure conditions. Debris and doublets were excluded based on FSC/SSC parameters, and dead cells were excluded using Fixable Viability Dye eFluor 780, and the acquired data were analyzed using FlowJo software v10 (FlowJo LLC).

### Statistical analysis

Statistical analyses were conducted using GraphPad Prism 8.0 (GraphPad Software). For two-group comparisons, a two-tailed or one-tailed unpaired *t*-test was used. For multigroup comparisons, one-way ANOVA with Tukey’s multiple comparison test or Dunnett’s multiple comparison test was used. Statistical analysis for survival curves was performed using the log-rank test. *P* values of 0.05 or less were the threshold for statistical significance. Error bars represent standard deviation (SD). The sample size of in vivo and in vitro studies was chosen to be as small as possible but to allow the evaluation of distribution normality.

## Supplementary information

Appendix

Peer Review File

Source data Fig. 1

Source data Fig. 2

Source data Fig. 3

Source data Fig. 4

Source data Fig. 5

Source data Fig. 6

Source data Fig. 7

Expanded View Figures

## Data Availability

No primary datasets have been generated and deposited for this study. The source data of this paper are collected in the following database record: biostudies:S-SCDT-10_1038-S44319-026-00918-x.
