# Supplementary material for: Aurora A exacerbates antibiotic-resistant Staphylococcus aureus infection through ASC phosphorylation
Source: EMBO Rep. 2026 Sep 7;27(20):5881–903. doi: 10.1038/s44319-026-00918-x (PMC13694275; doi:10.1038/s44319-026-00918-x)
Supplement: Supplementary file 1 [file 44319_2026_918_MOESM10_ESM.pdf]

Expanded View Figures

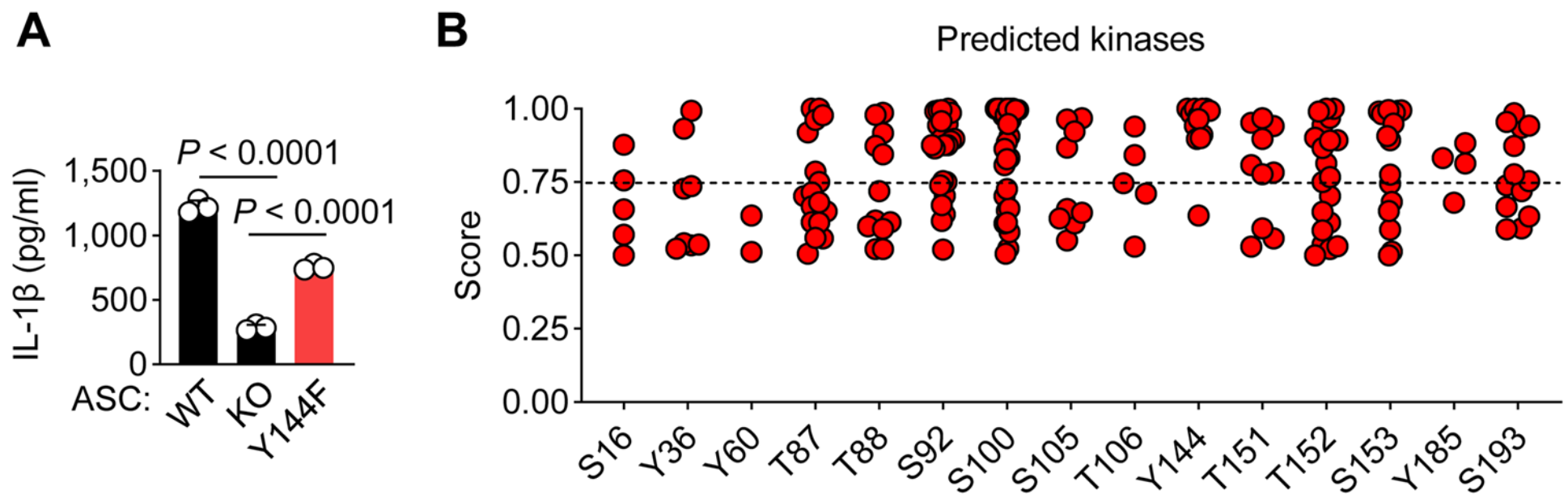

C

S92	
Kinase	Score
PKA	1
AURK	0.996
CDK	0.995
STE20	0.995
MAPK	0.993
PKC	0.992
PLK	0.986
PKG	0.959
AKT	0.948
CAMKL	0.944
CK1	0.898
CAMK2	0.897
IKK	0.891
GSK	0.882
DAPK	0.877
RSK	0.876
GRK	0.876
DMPK	0.865
RAD53	0.754
PKD	0.75
TTK	0.739
FJ	0.704
SGK	0.671
CK2	0.641
PIKK	0.618
ULK	0.52

S100	
Kinase	Score
CDK	1
PKA	1
MAPK	1
PKC	1
GSK	1
CAMK2	1
MAPKAPK	1
IKK	0.997
PKD	0.997
CAMKL	0.996
CK1	0.98
PIKK	0.976
PLK	0.963
RAD53	0.949
STE20	0.906
AURK	0.887
GRK	0.864
AKT	0.832
PDK1	0.828
RSK	0.809
PKN	0.726
ULK	0.703
CAMK1	0.661
MLK	0.653
BUB	0.612
STKR	0.61
Alpha	0.58
DMPK	0.523
SGK	0.507

S105	
Kinase	Score
CDK	0.968
PKA	0.868
MAPK	0.964
PKC	0.923
CK2	0.552
GSK	0.609
CAMK2	0.659
CK1	0.626
PDK1	0.646

Y144	
Kinase	Score
MAPK	1
SRC	1
ABL	0.984
EGFR	1
JAK	0.976
SYK	1
FAK	0.636
InsR	0.943
PDGFR	0.912
TEC	0.994
FGFR	0.966
MET	0.898
FER	0.901

Figure EV1. Prediction of phosphorylation sites in ASC.

(**A**) Cytokine levels in culture supernatants of ASC-reconstituted macrophages after infection with *S. aureus* NUMR101 strain. Bars represent the mean ± SD of three biological replicates. Statistical analysis was performed using one-way ANOVA followed by Tukey's multiple comparison test. $P < 0.0001$, $P < 0.0001$. (**B**, **C**) Kinases for murine ASC and phosphorylation sites were predicted using KinasePhos 3.0. Phosphorylation sites are shown on the x-axis, and the probability score is shown on the y-axis. Each dot indicates a kinase for the corresponding amino acid.

© The Author(s)

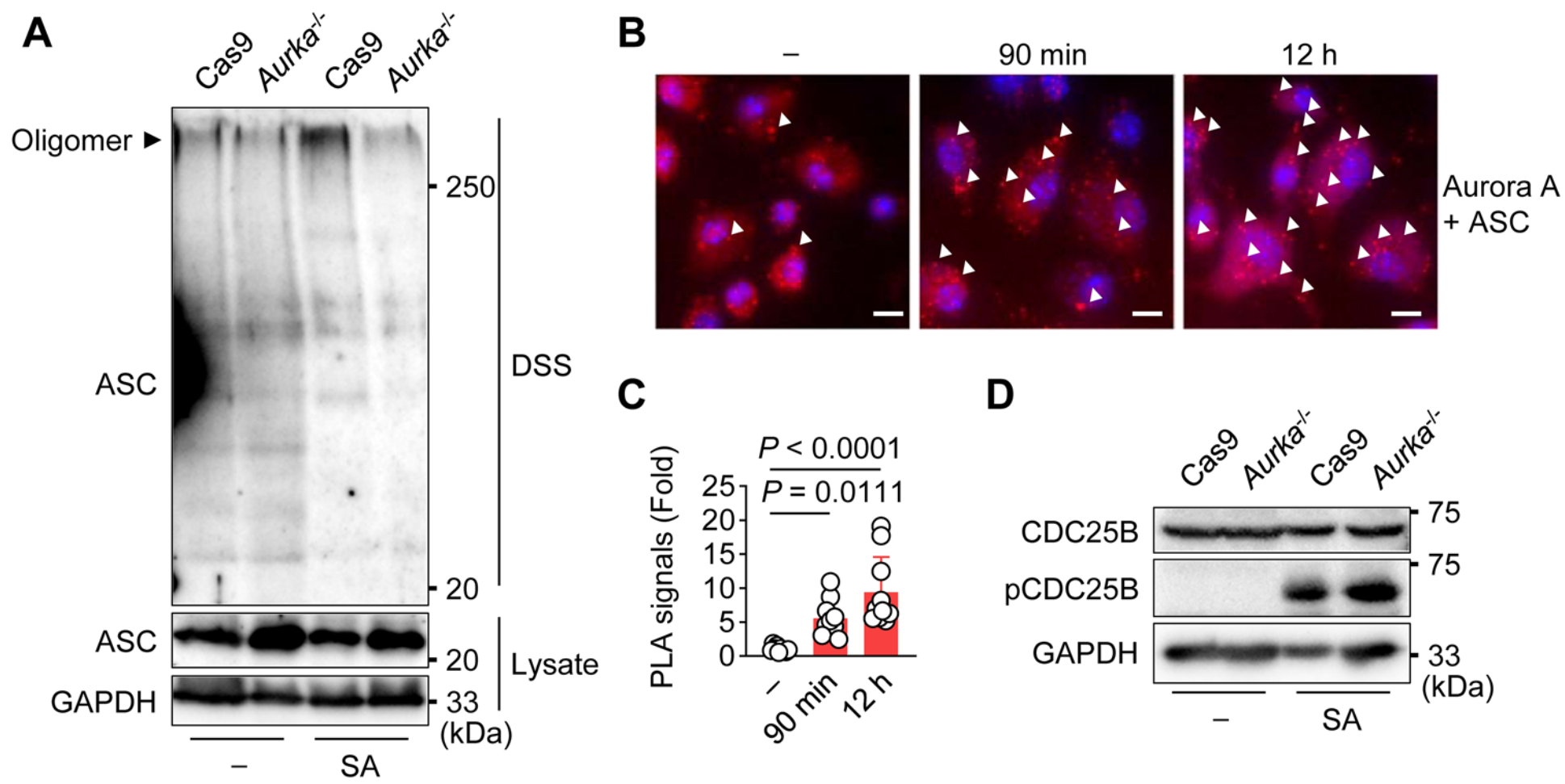

Figure EV2. Aurora A interacts with ASC during *S. aureus* infection.

(**A**) Immunoblotting of Triton X-100-soluble lysate (Lysate) and -insoluble fractions treated with DSS (DSS) of immortalized Aurora A-deficient BMDMs infected with *S. aureus* NUMR101 at MOI = 50 for 12 h. (**B**, **C**) In situ PLA of primary BMDMs infected with *S. aureus* NUMR101 at MOI = 50 for the indicated times. Arrowheads indicate interactions of Aurora A and ASC. Bars represent the mean ± SD of ten biological replicates. Statistical analysis was performed using one-way ANOVA followed by Dunnett's multiple comparison test. $P = 0.0111$, $P < 0.0001$. (**D**) Immunoblotting of cell lysates of Cas9-expressing immortalized wild-type or Aurora A-deficient BMDMs infected with *S. aureus* NUMR101 at MOI = 50 for 18 h.

 © The Author(s)

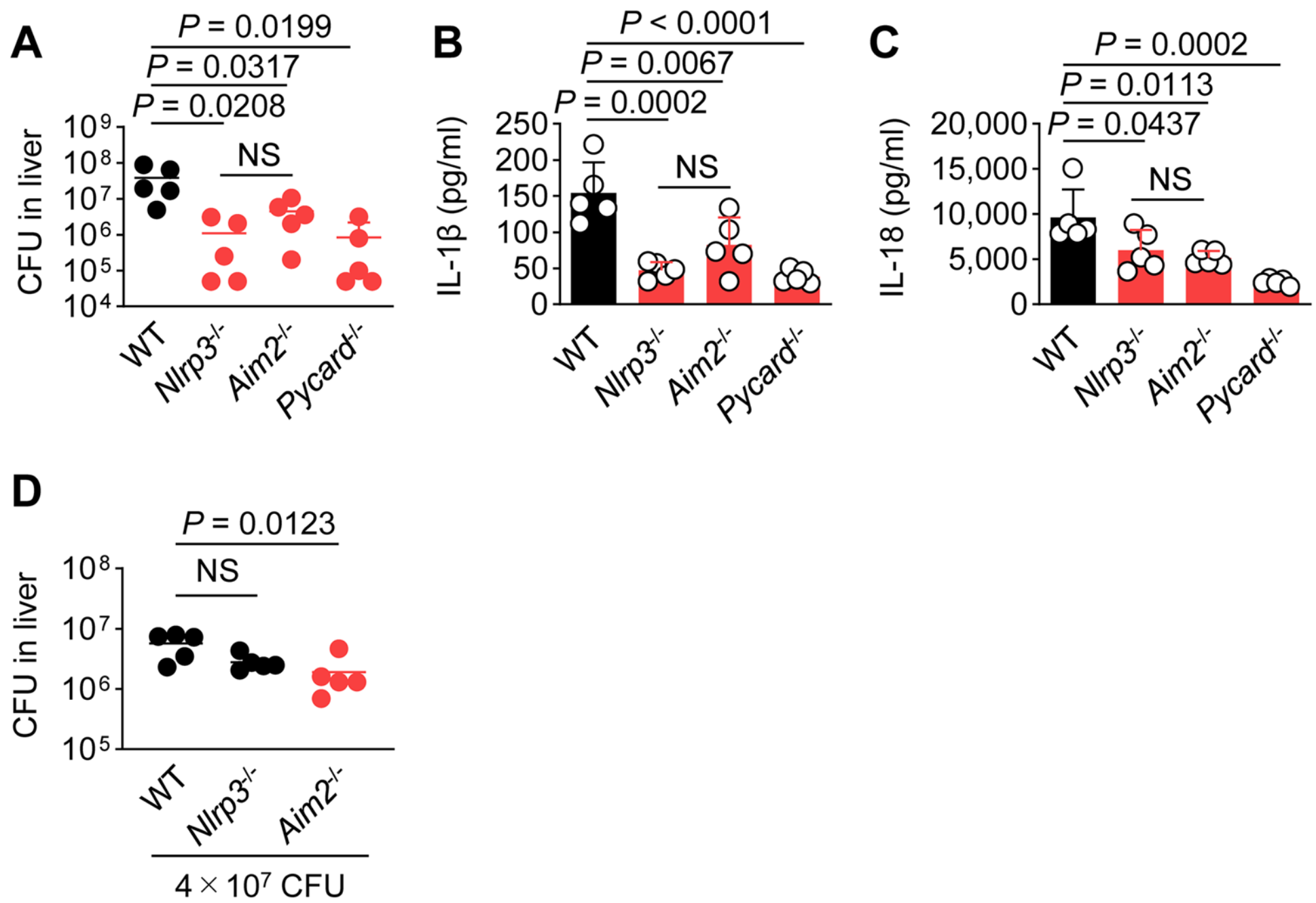

Figure EV3. Both NLRP3 and AIM2 inflammasome activations exacerbate *S. aureus* infection.

(**A**) CFU in the indicated organs on day 2 after infection intravenously with 1×10^8 CFU of *S. aureus* NUMR101. Representative data (mean) are shown from five biological replicates. Statistical analysis was performed using one-way ANOVA followed by Dunnett's multiple comparison test. (**A**) $P = 0.0208$, $P = 0.0319$, $P = 0.0199$. (**B**, **C**) Cytokine levels in the sera on day 2 after intravenous infection with 1×10^8 CFU of *S. aureus* NUMR101. Representative data (mean ± SD) are shown from five biological replicates. Statistical analysis was performed using one-way ANOVA followed by Dunnett's multiple comparison test. (**B**) $P = 0.0002$, $P = 0.0067$, $P < 0.0001$, (**C**) $P = 0.0437$, $P = 0.0113$, $P = 0.0002$. NS, no significant difference. (**D**) CFU in the indicated organs on day 2 after infection intravenously with 4×10^7 CFU of *S. aureus* NUMR101. Representative data (mean) are shown from five biological replicates. Statistical analysis was performed using one-way ANOVA followed by Dunnett's multiple comparison test. (**D**) $P = 0.0123$. NS no significant difference.

© The Author(s)

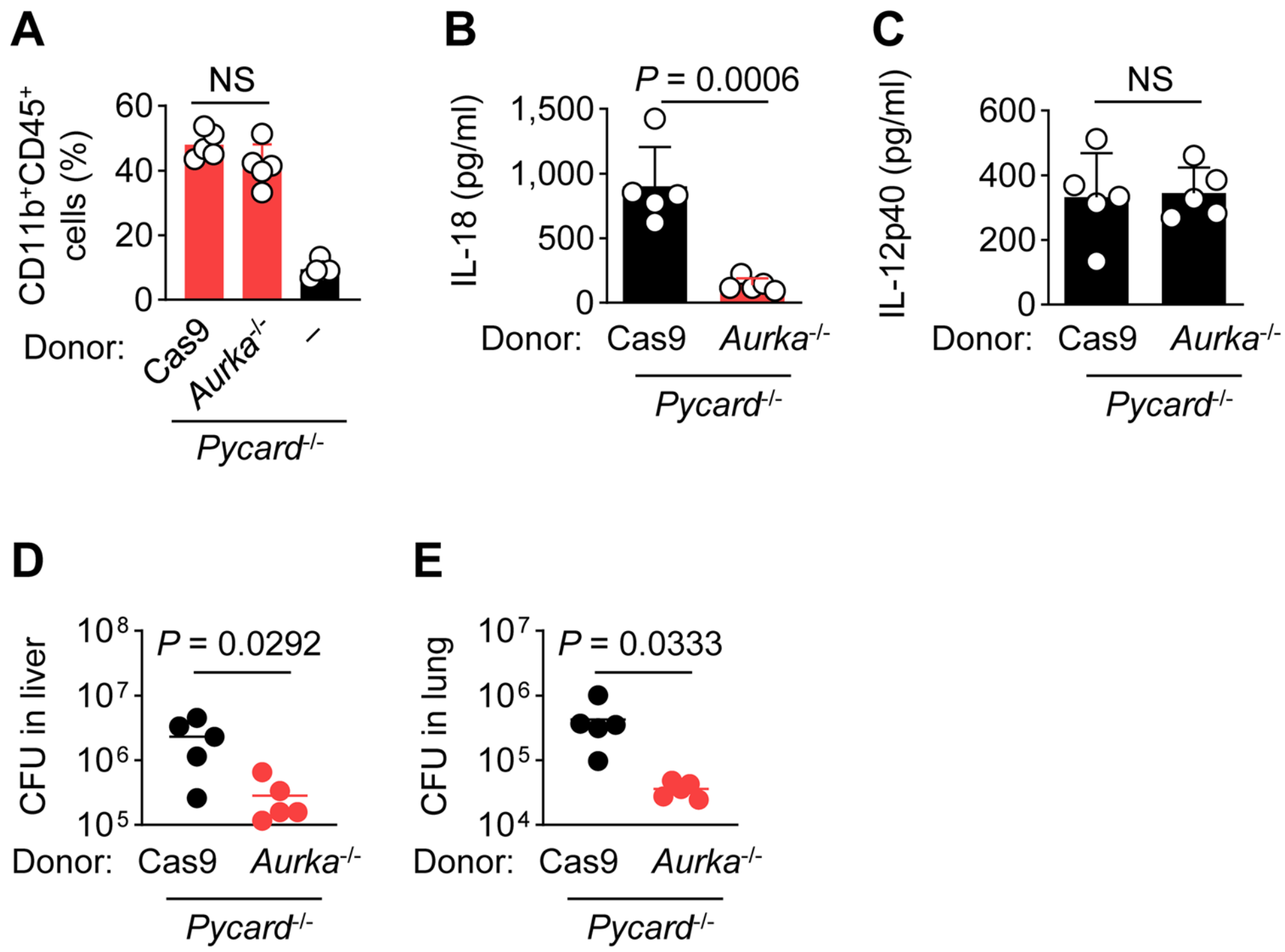

Figure EV4. Adoptive transfer of *Aurka*$^{-/-}$ BMDMs reduces the bacterial burden in mice infected with *S. aureus.*

(**A**) Percentage of CD11b$^+$CD45$^+$ cells in the lung with or without transplantation of immortalized Cas9 or *Aurka*$^{-/-}$ BMDMs into *Pycard*$^{-/-}$ mice. Representative data (mean ± SD) are shown from five biological replicates. Statistical analysis was performed using one-way ANOVA followed by Tukey's multiple comparison test. NS, no significant difference. (**B**, **C**) Cytokine levels in the sera and (**D**, **E**) CFU in the indicated organs on day 2 after intravenous infection with 4×10^7 CFU of *S. aureus* NUMR101, followed by macrophage transplantation (Donor). Representative data (**B**, **C**, mean ± SD; **D**, **E**, mean) are shown from five biological replicates. Statistical analysis was performed using a two-tailed t-test. (**B**) $P = 0.0006$, (**D**) $P = 0.0292$, (**E**) $P = 0.0333$. NS no significant difference.

© The Author(s)

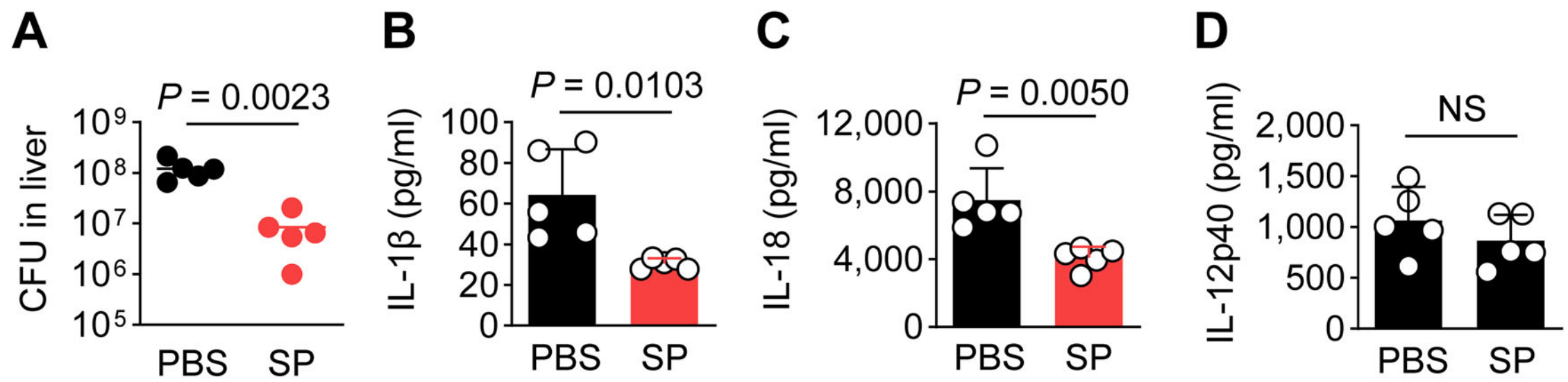

Figure EV5. JNK inhibitor ameliorates *S. aureus* infection in vivo.

(**A**) CFU in the indicated organs and (**B**–**D**) cytokine levels in the sera on day 2 after infection intravenously with 2×10^8 CFU of *S. aureus* NUMR101. SP600125, a JNK inhibitor, was administered by intraperitoneal injection (1 mg/mouse) on 3 and 24 h after infection. Representative data (**A**, mean; **B**–**D**, mean ± SD or mean) are shown from five biological replicates. Statistical analysis was performed using a two-tailed *t*-test. (**A**) $P = 0.0023$, (**B**) $P = 0.0103$, (**C**) $P = 0.0050$. NS no significant difference.
