# Supplementary material for: Aurora A exacerbates antibiotic-resistant Staphylococcus aureus infection through ASC phosphorylation
Source: EMBO Rep. 2026 Sep 7;27(20):5881–903. doi: 10.1038/s44319-026-00918-x (PMC13694275; doi:10.1038/s44319-026-00918-x)
Supplement: Supplementary file 2 [file 44319_2026_918_MOESM1_ESM.pdf]

Appendix for:

Aurora A Exacerbates Antibiotic-Resistant Staphylococcus aureus Infection through ASC Phosphorylation

Table of Contents

• Appendix Figure S1, page 2

• Appendix Figure S2, pages 3, 4

• Appendix Figure S3, page 5

• Appendix Figure S4, page 6

• Appendix Figure S5, page 7

• Appendix Figure S6, page 8

• Appendix Figure S7, page 9

• Appendix Figure S8, page 10

• Appendix Figure S9, page 11

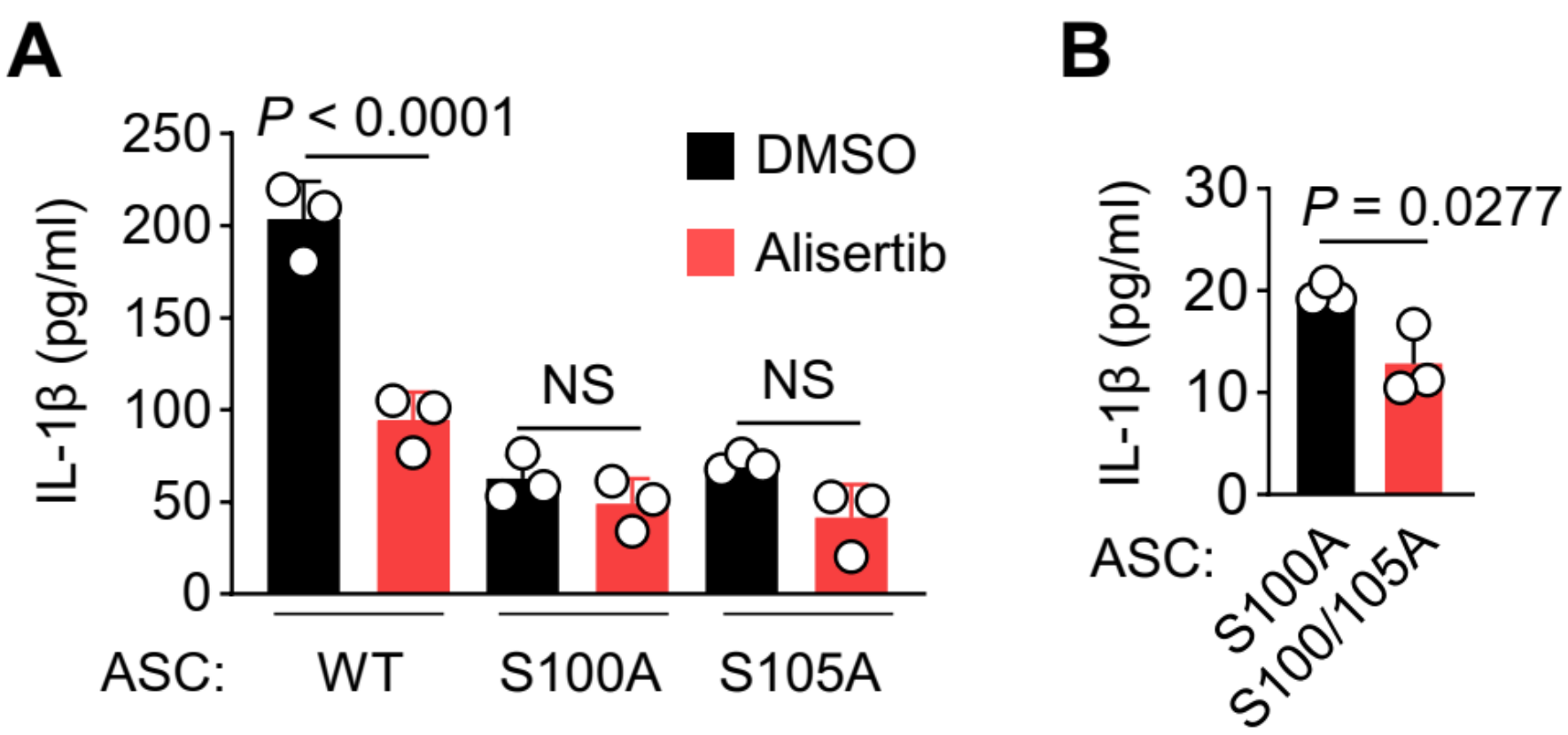

Appendix Fig. S1. ASC S100 and S105 are involved in the IL-1β production induced by *S. aureus* infection.

(**A, B**) Cytokine levels in cell supernatants of *Pycard*$^{-/-}$ immortalized macrophages reconstituted with ASC wild-type, indicated mutants after infection with *S. aureus* NUMR101 at MOI = 10 for 12 h. (**A**) Cells were treated with 10 µM Alisertib 30 min before infection. Bars represent the mean ± SD of three biological replicates. Statistical analysis was performed using (**A**) one-way ANOVA followed by Tukey's multiple comparison test or (**B**) two-tailed t-test. (**A**) *P* < 0.0001, (**B**) *P* = 0.0277. NS, no significant difference.

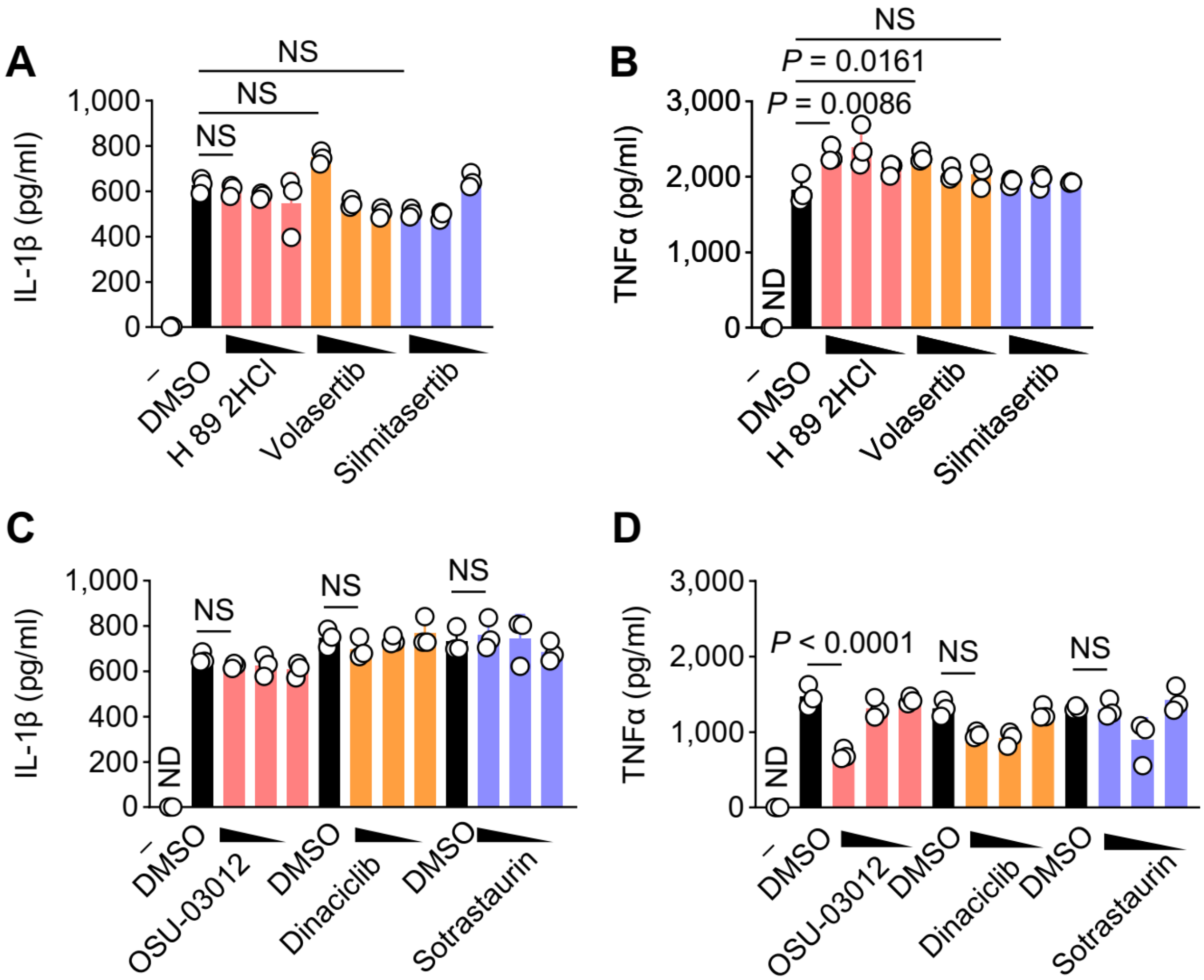

Appendix Fig. S2. Screening of kinases that affect IL-1β production in response to *S. aureus* infection.

Cytokine levels in cell supernatants of peritoneal macrophages infected with *S. aureus* NUMR101 at MOI = 25 for 18 h. Cells were treated with H 89 2HCl (PKA inhibitor; 500, 50, and 5 nM), Volasertib (PLK inhibitor; 500, 50, and 5 nM), Silmitasertib (CK2 inhibitor; 500, 50, and 5 nM), OSU-03012 (PDK1 inhibitor; 20, 2, 0.2 μM), Dinaciclib (CDK inhibitor;

500, 50, and 5 nM), and Sotrastaurin (PKC inhibitor; 500, 50, and 5 nM) 30 min before infection. Results are representative of two biological replicates, and error bars denote SD. Bars represent the mean ± SD of three biological replicates. Statistical analysis was performed using one-way ANOVA followed by Tukey's multiple comparison test. (**B**) $P = 0.0086$, $P = 0.0161$, (**D**) $P < 0.0001$. NS, no significant difference. ND, not detected.

Aurka gene sequence of Cas9 iBMDMs

```
  1 atggctgttgagggcgagcctggatgctgcaaacggatagggaaggctgtgtggcgtcgg  60
     M  A  V  E  G  E  P  G  C  C  K  R  I  G  K  A  V  W  R  R

 61 ggtgacatggacagatgtaaagaaaactgtgtctccaggcctgttaagaccactgttccc 120
     G  D  M  D  R  C  K  E  N  C  V  S  R  P  V  K  T  T  V  P

121 ttcggtccgaaacgcgtcttggtgactgagcagattccgtctcagaacctaggatctgct 180
     F  G  P  K  R  V  L  V  T  E  Q  I  P  S  Q  N  L  G  S  A
```

Aurka gene sequence of *Aurka*$^{-/-}$ iBMDMs

```
  1 atggctgttgagggcgagcctggatgctgcaaacggatagggaaggctgtgtggcgtcgg  60
     M  A  V  E  G  E  P  G  C  C  K  R  I  G  K  A  V  W  R  R

 61 ggtgacatggacagatgtaaagaaaactgtgtctccaggcctgttaagaccactgttccc 120
     G  D  M  D  R  C  K  E  N  C  V  S  R  P  V  K  T  T  V  P

121 ttcggaacagaacctaggatctgctag 147
     F  G  T  E  P  R  I  C  *
```

Appendix Fig. S3. Genome sequence of *Aurka*$^{-/-}$ immortalized BMDMs.

Mutations in *Aurka* were determined by subcloning and DNA sequencing. * indicates stop codon.

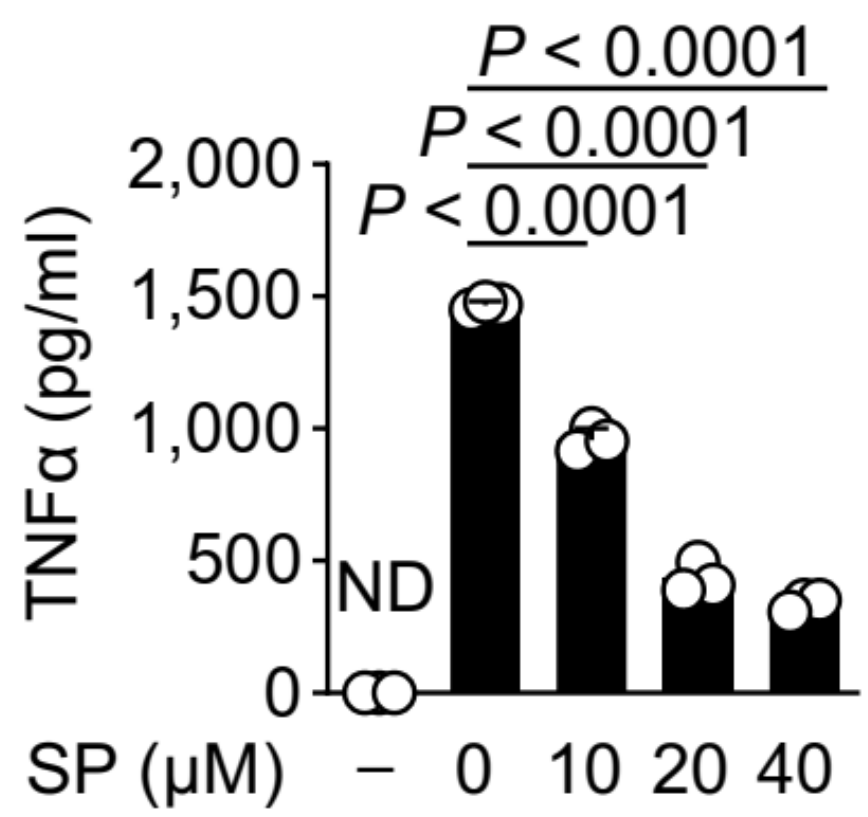

Appendix Fig. S4. Treatment with an JNK kinase inhibitor reduces TNFα production.

Cytokine levels in cell supernatants of peritoneal macrophages infected with *S. aureus* NUMR101 at MOI = 25 for 18 h. Cells were treated with SP600125 (SP, JNK inhibitor, 10, 20, and 40 μM) 30 min before infection. Bars represent the mean ± SD of three biological replicates. Statistical analysis was performed using one-way ANOVA followed by Dunnett's multiple comparison test. $P < 0.0001$, $P < 0.0001$, $P < 0.0001$. ND, not detected.

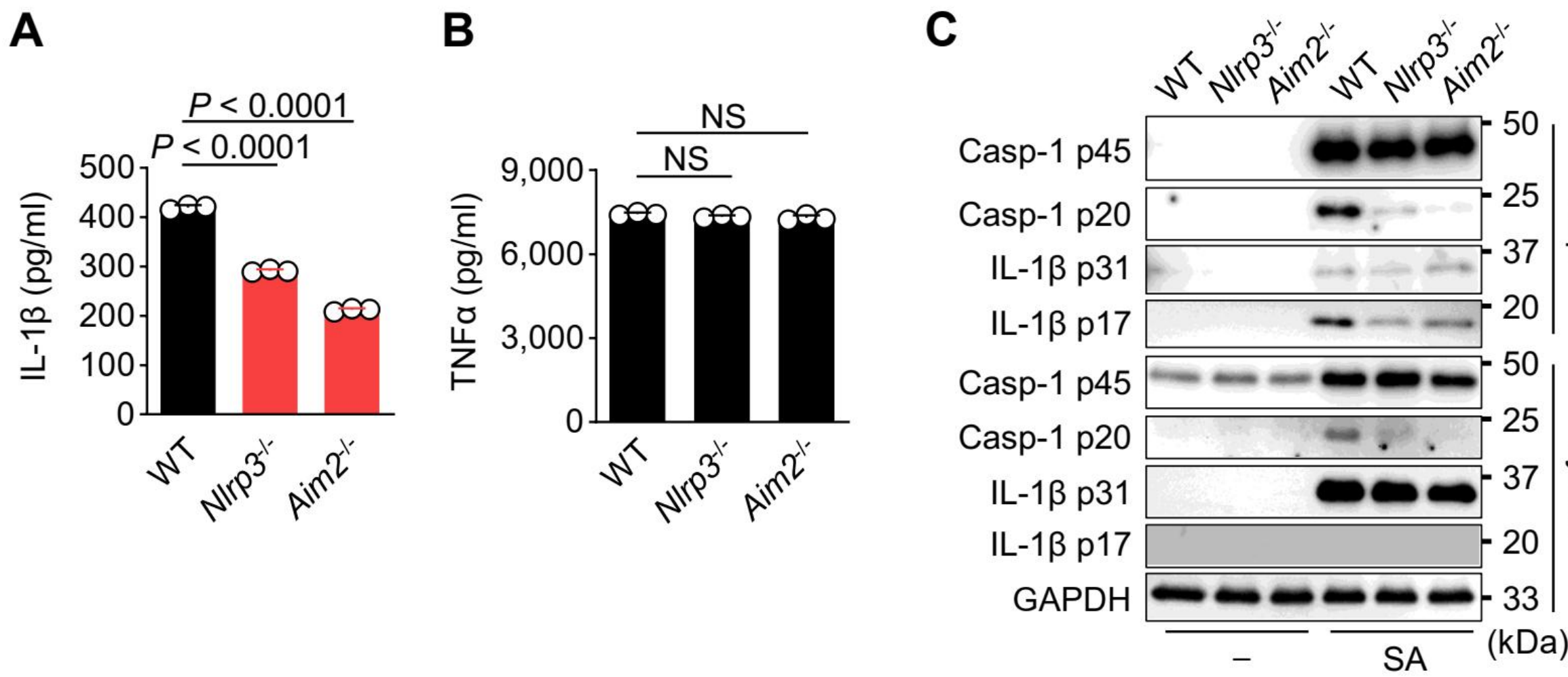

Appendix Fig. S5. *S. aureus* activates both NLRP3 and AIM2 inflammasomes.

(**A, B**) Cytokine levels in cell supernatants or (**C**) immunoblotting of wild-type, *Nlrp3*$^{-/-}$ and *Aim2*$^{-/-}$ primary BMDMs infected with *S. aureus* NUMR101 at MOI = 25 for 24 h. Bars represent the mean ± SD of three biological replicates. Statistical analysis was performed using one-way ANOVA followed by Tukey's multiple comparison test. (**A**) $P < 0.0001$, $P < 0.0001$. NS, no significant difference.

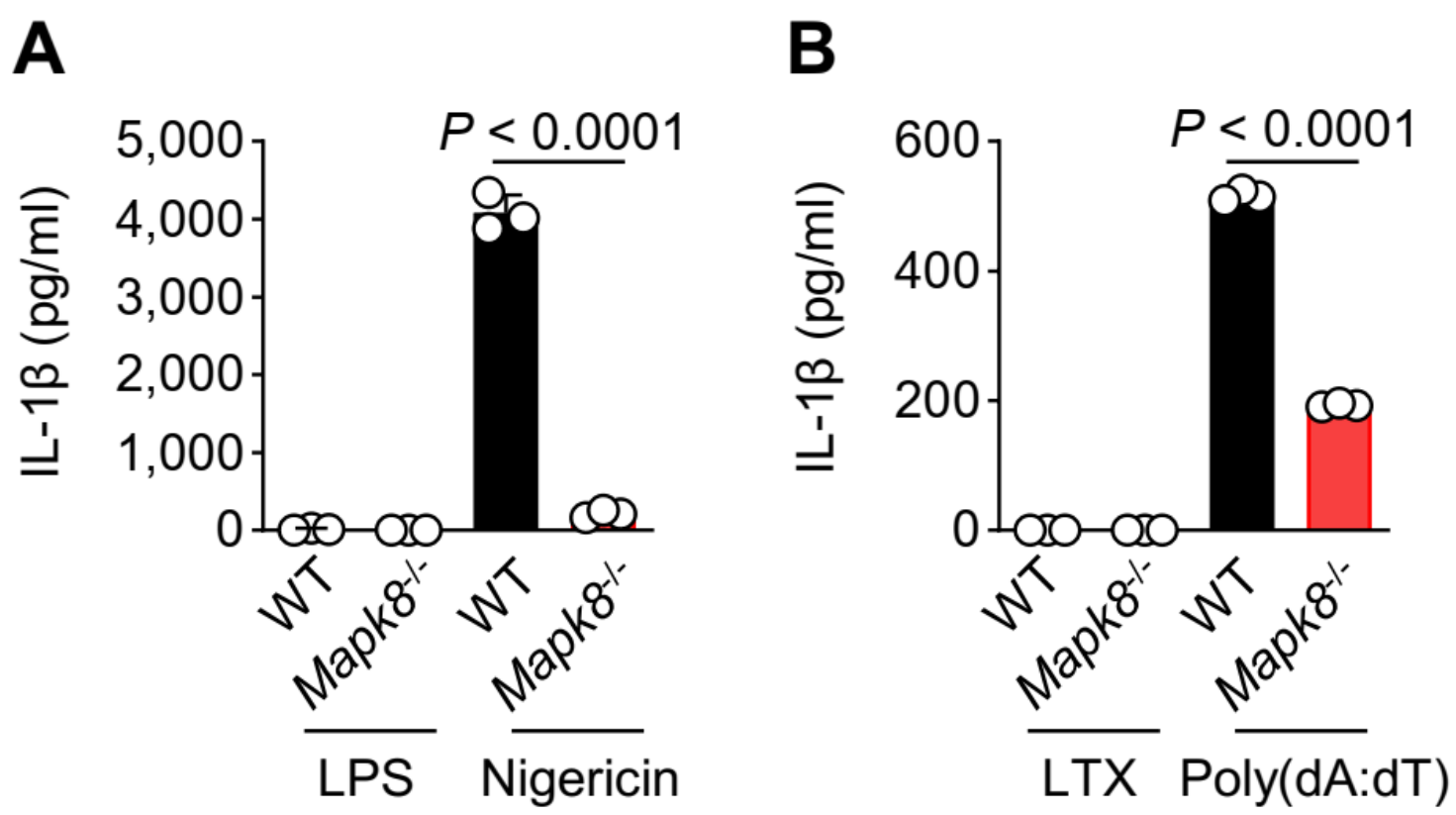

Appendix Fig. S6. JNK1 is required for both NLRP3 and AIM2 inflammasome activation.

Cytokine levels in cell supernatants of LPS-primed immortalized WT or *Mapk8*$^{-/-}$ BMDMs stimulated with nigericin for 60 min or poly(dA:dT) plus Lipofectamine LTX for 120 min. Bars represent the mean ± SD of three biological replicates. Statistical analysis was performed using one-way ANOVA followed by Tukey's multiple comparison test. (**A**) $P < 0.0001$, (**B**) $P < 0.0001$.

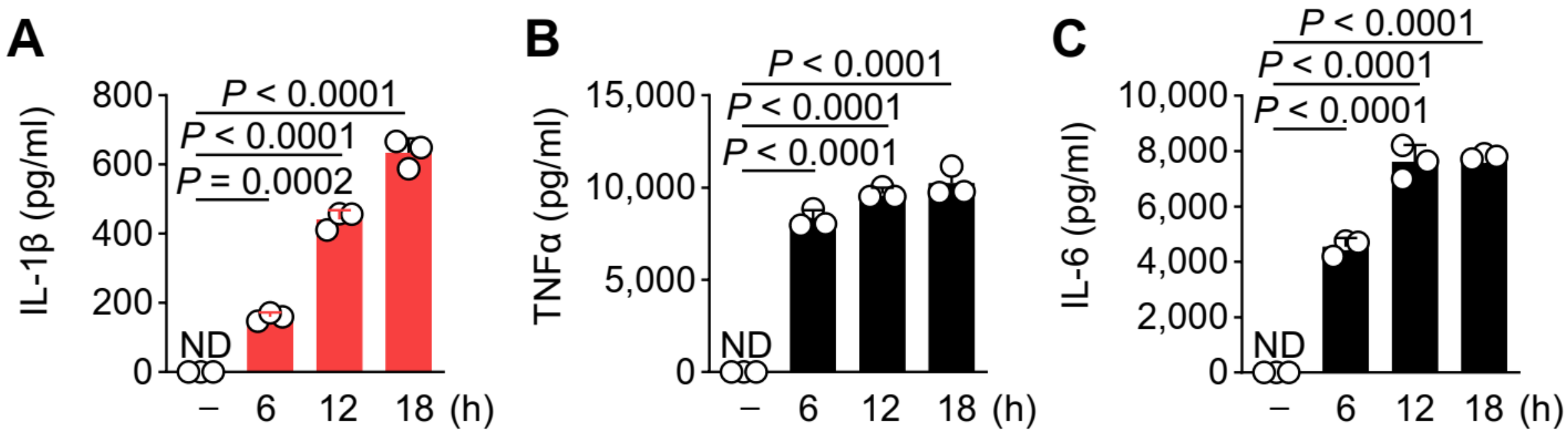

Appendix Fig. S7. Time-course analysis of cytokine production induced by *S. aureus* infection.

Cytokine levels in cell supernatants of immortalized wild-type BMDMs infected with *S. aureus* NUMR101 at MOI = 50 for indicated times. Bars represent the mean ± SD of three biological replicates. Statistical analysis was performed using one-way ANOVA followed by Dunnett's multiple comparison test. (**A**) *P* = 0.0002, *P* < 0.0001, *P* < 0.0001, (**B**) *P* < 0.0001, *P* < 0.0001, *P* < 0.0001, (**C**) *P* < 0.0001, *P* < 0.0001, *P* < 0.0001. ND, not detected.

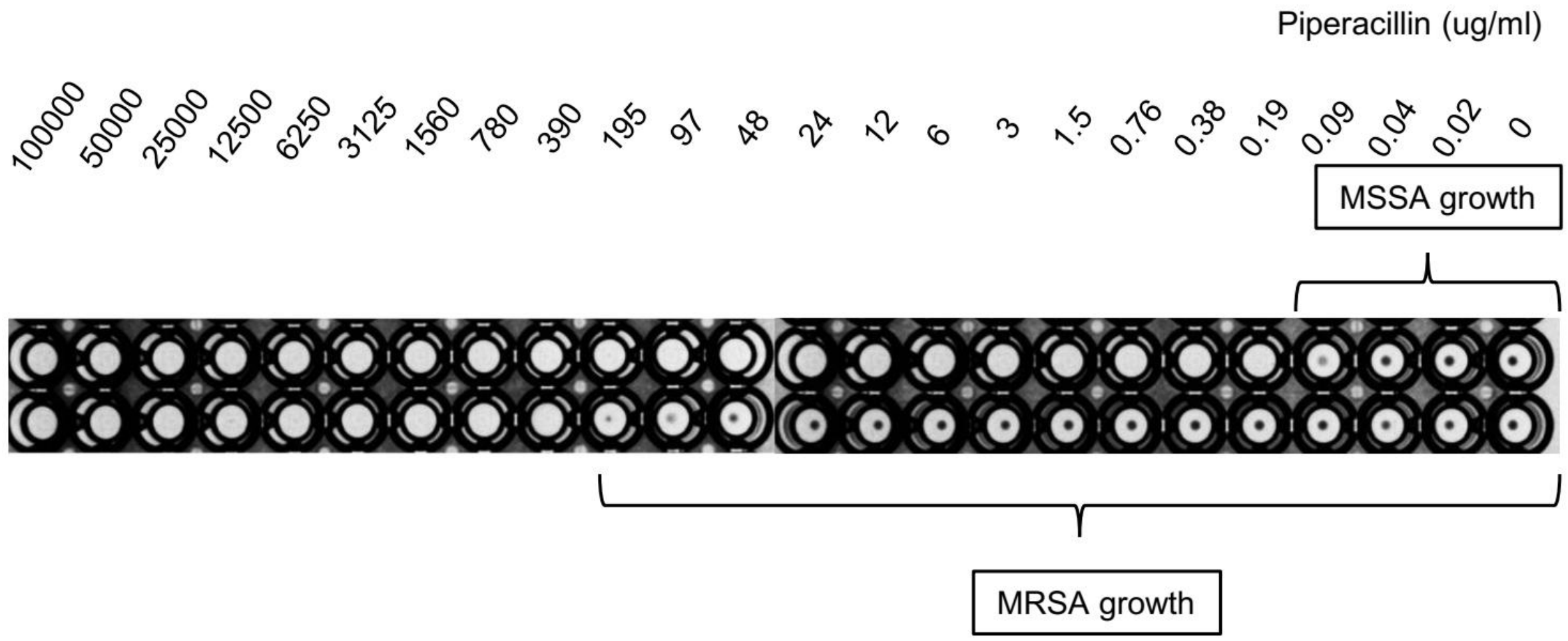

Appendix Fig. S8. Piperacillin sensitivity of MSSA and MRSA.

Piperacillin sensitivity of MSSA and MRSA (NUMR101) was determined by microdilution method. Bacterial precipitations were observed as dark spots at the center of the wells.

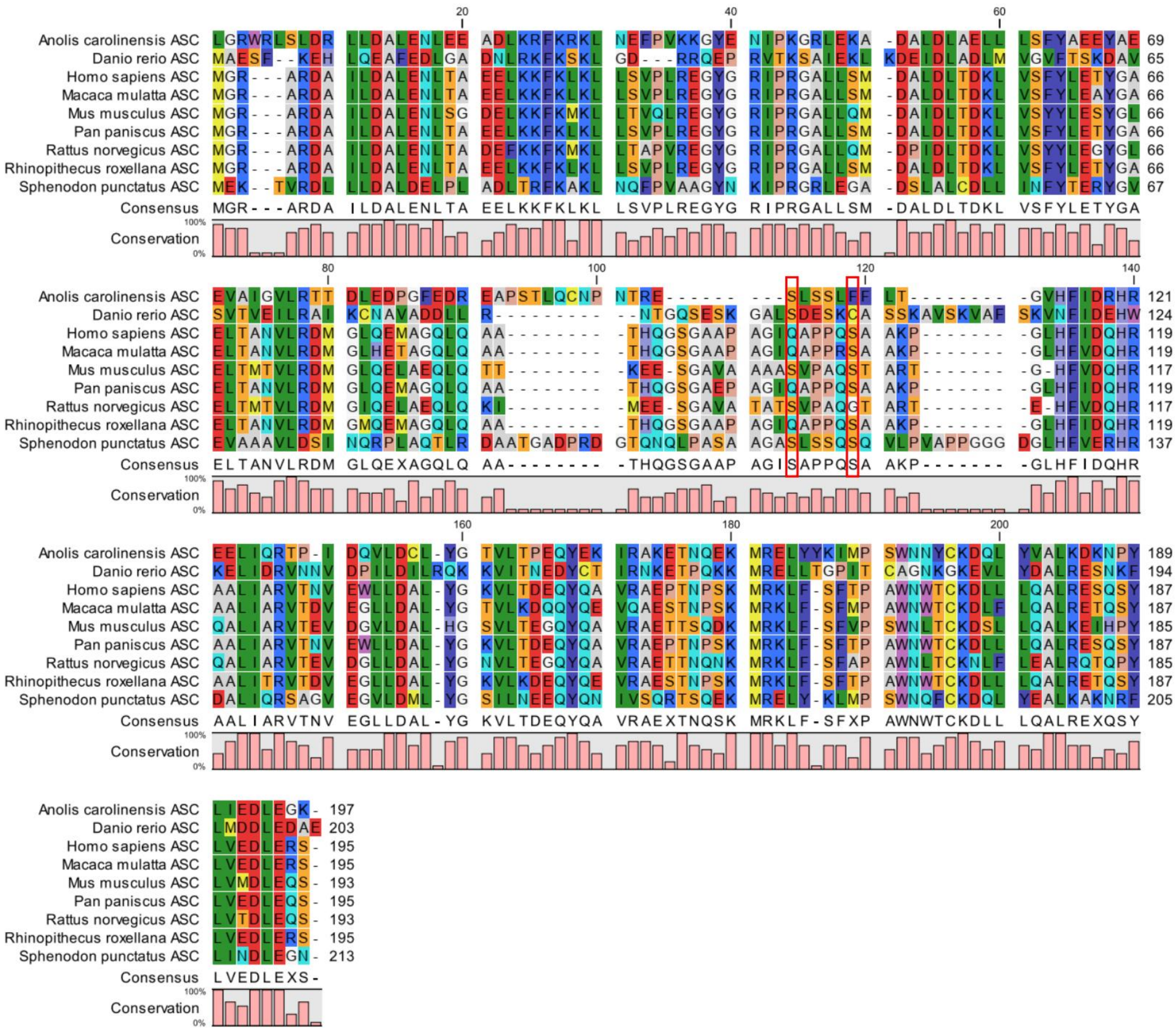

Appendix Fig. S9. Conservation of the ASC serine residues across nine animal species.

Amino acid sequences of ASC, consensus amino acid, conservation from nine animal species. The amino acids corresponding to mouse ASC S100 and S105 are highlighted with red boxes.
