# Supplementary material for: Aurora A exacerbates antibiotic-resistant Staphylococcus aureus infection through ASC phosphorylation
Source: EMBO Rep. 2026 Sep 7;27(20):5881–903. doi: 10.1038/s44319-026-00918-x (PMC13694275; doi:10.1038/s44319-026-00918-x)
Supplement: Supplementary file 3 [file 44319_2026_918_MOESM2_ESM.pdf]

Aurora A Exacerbates Antibiotic-Resistant Staphylococcus aureus Infection through ASC Phosphorylation

Hajime Yamauchi, Kei Sakamoto, Yasuyuki Matsuda, Yuko Tanishita, Akito Ariyoshi, Daisuke Sasaki, Yu Shimoyama, Akihiro Hasegawa, Takayoshi Yamauchi, Yoichiro Iwakura, Haruki Okamura, Shun'ichiro Taniguchi, Masafumi Takahashi, Akihiko Yoshimura, Katsunori Yanagihara, Gabriel Nuñez, and Hideki Hara

Corresponding author(s): Hideki Hara (hhara@asahikawa-med.ac.jp) , Kei Sakamoto (sakamotk@yamaguchi-u.ac.jp)

Review Timeline:

Submission Date:	1st Dec 25
Editorial Decision:	29th Jan 26
Revision Received:	30th Jun 26
Editorial Decision:	13th Aug 26
Revision Received:	20th Aug 26
Accepted:	22nd Aug 26

Editor: Achim Breiling

Transaction Report:

(Note: With the exception of the correction of typographical or spelling errors that could be a source of ambiguity, letters and reports are not edited. Depending on transfer agreements, referee reports obtained elsewhere may or may not be included in this compilation. Referee reports are anonymous unless the Referee chooses to sign their reports.)

1st Editorial Decision 29th Jan 2026

Dear Prof. Hara,

Thank you for the re-submission of your manuscript to EMBO reports. I have now received reports from the three referees that were asked to evaluate your study, which can be found at the end of this email. As you will see, the referees have several comments, concerns, and suggestions, indicating that a major revision of the manuscript is necessary to allow publication of the study in EMBO reports. As the reports are below, and all points need to be addressed, I will not detail them here.

Given the constructive referee comments, I would thus like to invite you to revise your manuscript with the understanding that the concerns of the referees must be addressed in the revised manuscript and in a detailed point-by-point response. Acceptance of your manuscript will depend on a positive outcome of a second round of review. It is EMBO reports policy to allow a single round of revision only and acceptance of the manuscript will therefore depend on the completeness of your responses included in the next, final version of the manuscript.

Revised manuscripts should be submitted within three months of a request for revision. Please contact me to discuss the revision (also by video chat) if you have questions or comments regarding the revision, or should you need additional time.

When submitting your revised manuscript, please also carefully review the instructions that follow below.

PLEASE NOTE THAT upon resubmission revised manuscripts are subjected to an initial quality control prior to exposition to re-review. Upon failure in the initial quality control, the manuscripts are sent back to the authors, which may lead to delays. Frequent reasons for such a failure are the lack of the data availability section (please see below) and the presence of statistics based on n=2 (the authors are then asked to present scatter plots or provide more data points).

When submitting your revised manuscript, we will require:

1) a .docx formatted version of the final manuscript text (including legends for main figures, EV figures and tables), but without the figures included. Figure legends should be compiled at the end of the manuscript text.

2) individual production quality figure files as .eps, .tif, .jpg (one file per figure), of main figures and EV figures. Please upload these as separate, individual files upon re-submission.

The Expanded View format, which will be displayed in the main HTML of the paper in a collapsible format, has replaced the Supplementary information. You can submit up to 6 images as Expanded View. Please follow the nomenclature Figure EV1, Figure EV2 etc. The figure legend for these should be included in the main manuscript document file in a section called Expanded View Figure Legends after the main Figure Legends section. Additional Supplementary material should be supplied as a single pdf file labeled Appendix. The Appendix should have page numbers and needs to include a table of content on the first page (with page numbers) and legends for all content. Please follow the nomenclature Appendix Figure Sx, Appendix Table Sx etc. throughout the text, and also label the figures and tables according to this nomenclature.

For more details, please refer to our guide to authors:
https://link.springer.com/journal/44319/submission-guidelines#cms-Revised-submissions

Please consult our guide for figure preparation:
https://media.springernature.com/original/springer-cms/rest/v1/content/27825798/data/v1

3) a .docx formatted letter INCLUDING the reviewers' reports and your detailed point-by-point responses to their comments. As part of the EMBO Press transparent editorial process, the point-by-point response is part of the Review Process File (RPF), which will be published alongside your paper.

4) a complete author checklist, which you can download from our author guidelines (https://link.springer.com/journal/44319/submission-guidelines#cms-Revised-submissions). Please insert page numbers in the checklist to indicate where the requested information can be found in the manuscript. The completed author checklist will also be part of the RPF.

Please also follow our guidelines for the use of living organisms, and the respective reporting guidelines:
https://link.springer.com/partners/embo-press/editorial-policies#Animal%20and%20human%20studies

5) that primary datasets produced in this study (e.g. RNA-seq, ChIP-seq, structural and array data) are deposited in an appropriate public database. If no primary datasets have been deposited, please also state this in a dedicated section (e.g. 'No primary datasets have been generated and deposited'), see below.

See also: https://link.springer.com/partners/embo-press/editorial-policies#Data%20deposition

Please remember to provide a reviewer password if the datasets are not yet public.

The accession numbers and database should be listed in a formal "Data Availability" section that follows the model below. This is now mandatory (like the COI statement). Please note that the Data Availability Section is restricted to new primary data that are part of this study. This section is mandatory. As indicated above, if no primary datasets have been deposited, please state this in this section

Data availability

The datasets produced in this study are available in the following databases:

- RNA-Seq data: Gene Expression Omnibus GSE46843 (https://www.ncbi.nlm.nih.gov/geo/query/acc.cgi?acc=GSE46843)
- [data type]: [name of the resource] [accession number/identifier/doi] ([URL or identifiers.org/DATABASE:ACCESSION])

*** Note - All links should resolve to a page where the data can be accessed. ***

See also: https://link.springer.com/partners/embo-press/editorial-policies#Data%20availability%20statement

Moreover, I have these editorial requests:

6) We now request the publication of original source data with the aim of making primary data more accessible and transparent to the reader. You will receive a separate email with instructions for providing source data with your revised manuscript, including information how to upload and organize the files.

7) Our journal encourages inclusion of *data citations in the reference list* to directly cite datasets that were re-used and obtained from public databases. Data citations in the article text are distinct from normal bibliographical citations and should directly link to the database records from which the data can be accessed. In the main text, data citations are formatted as follows: "Data ref: Smith et al, 2001" or "Data ref: NCBI Sequence Read Archive PRJNA342805, 2017". In the Reference list, data citations must be labeled with "[DATASET]". A data reference must provide the database name, accession number/identifiers and a resolvable link to the landing page from which the data can be accessed at the end of the reference. Further instructions are available at: https://link.springer.com/journal/44319/submission-guidelines#cms-Reference-guidelines

8) Regarding data quantification and statistics, please make sure that the number "n" for how many independent experiments were performed, their nature (biological versus technical replicates), the bars and error bars (e.g. SEM, SD) and the test used to calculate p-values is indicated in the respective figure legends (also for EV and Appendix figures). Please also check that all the p-values are explained in the legend, and that these fit to those shown in the figure. Please provide statistical testing where applicable. Please avoid the phrase 'independent experiment' but clearly state if these were biological or technical replicates. Please also indicate (e.g. with n.s.) if testing was performed, but the differences are not significant. In case n=2, please show the data as separate datapoints without error bars and statistics. See also:
https://link.springer.com/journal/44319/submission-guidelines#cms-Figure-and-data-presentation

If n<5, please show single datapoints for diagrams.

9) Please add scale bars of similar style and thickness to all microscopic images, using clearly visible black or white bars (depending on the background). Please place these in the lower right corner of the images themselves. Please do not write on or near the bars in the image but define the size in the respective figure legend.

10) Please also note our reference format:
https://link.springer.com/journal/44319/submission-guidelines#cms-Reference-guidelines

11) We updated our journal's competing interests policy in January 2022 and request authors to consider both actual and perceived competing interests. Please name this section 'Disclosure and Competing Interests Statement' and put it after the Acknowledgements section.
See: https://link.springer.com/partners/embo-press/editorial-policies#Competing%20interest%20disclosures

12) We now use CRediT to specify the contributions of each author in the journal submission system. CRediT replaces the author contribution section. Please use the free text box to provide more detailed descriptions and do NOT provide your final manuscript text file with an author contributions section. See also our guide to authors (section 'Author contributions'):
https://link.springer.com/journal/44319/submission-guidelines#cms-Revised-submissions

13) All Materials and Methods need to be described in the main text using our 'Structured Methods' format, which is required for all research articles. According to this format, the Methods section should include a Reagents and Tools Table (listing key reagents, experimental models, software, and relevant equipment and including their sources and relevant identifiers), uploaded

as separate file, and a Methods section in which we encourage the authors to describe their methods using a step-by-step protocol format with bullet points, to facilitate the adoption of the methodologies across labs. More information on how to adhere to this format as well as downloadable templates (.doc) for the Reagents and Tools Table can be found in our author guidelines (section 'Structured Methods'):

https://link.springer.com/journal/44319/submission-guidelines#cms-Manuscript-organisation-and-formatting

Please add all primer or antibody information directly to the Reagents and Tools table.

14) Please order the manuscript sections like this, using only these names:
Title page - Abstract (max. 175 words) - Keywords (max. 5) - Introduction - Results - Discussion - Methods - Data availability section - Acknowledgements (please include here all the funding information) - Disclosure and Competing Interests Statement - References - Figure legends - Expanded View Figure legends

15) Please make sure that all the funding information is also entered into the online submission system and that it is complete and similar to the one in the acknowledgement section of the manuscript text file.

16) Please also confirm that for all Western blot panels shown the loading control was run on the same gel as the other proteins detected. Please note that we discourage comparisons between samples on different gels/blots, even if the samples derive from one experiment, as confounding factors reduce comparability. If unavoidable, the figure legend must state that the samples derive from the same experiment and that gels/blots were processed in parallel. If a 'representative' loading control is shown for multiple gels/blots, the intra-gel controls should be shown in the source data files, and the figure legends should describe the data displayed accurately. See our author guidelines:

https://link.springer.com/journal/44319/submission-guidelines#cms-Figure-and-data-presentation (section 'Electrophoretic gels and blots').

We would also welcome the submission of cover suggestions, or motifs to be used by our Graphics Illustrator in designing a cover.

As part of the EMBO publication's Transparent Editorial Process, EMBO reports publishes online a Review Process File (RPF) to accompany accepted manuscripts. This File will be published in conjunction with your paper and will include the referee reports, your point-by-point response and all pertinent correspondence relating to the manuscript.

You are able to opt out of this by letting the editorial office know (emboreports@embo.org). If you do opt out, the Review Process File link will point to the following statement: "No Review Process File is available with this article, as the authors have chosen not to make the review process public in this case."

I look forward to seeing a revised form of your manuscript when it is ready.

Yours sincerely,

Achim Breiling
Senior Editor
EMBO Reports

Referee #1:

1 The manuscript proposes a JNK-Aurora A signaling axis in S. aureus-induced inflammasome activation. While the authors convincingly show that JNK deficiency or pharmacological inhibition reduces Aurora A phosphorylation and downstream caspase-1 activation, the mechanistic link between JNK and Aurora A remains insufficiently defined. Specifically, it is unclear whether JNK directly phosphorylates Aurora A or whether Aurora A activation is regulated indirectly through intermediate kinases, phosphatases, or cofactors. The study does not provide biochemical evidence (e.g., in vitro kinase assays, binding assays, or phosphorylation-site analysis) to support a direct JNK-Aurora A interaction. Given that Aurora A activation is classically regulated by autophosphorylation and co-factors, it is important to clarify how JNK signaling mechanistically converges on Aurora A in this context. Without defining this molecular connection, the proposed JNK-Aurora A axis remains correlative rather than mechanistically established.

2 The manuscript proposes that Aurora A-mediated ASC phosphorylation is required for both AIM2 and NLRP3 inflammasome activation during S. aureus infection. At the same time, Figure 5 demonstrates that bacterial DNA is released into the cytosol of infected macrophages, and Figure 6 further shows that AIM2 deficiency alone markedly improves survival, reduces bacterial

burden, and decreases IL-1β and IL-18 production in vivo. These data strongly suggest that DNA-driven AIM2 activation is a dominant pathogenic pathway in this infection model. This raises a conceptual ambiguity in the current model: if cytosolic bacterial DNA-AIM2 signaling is sufficient to drive inflammasome-dependent pathology in vivo, what is the relative contribution of Aurora A-mediated ASC phosphorylation? Is Aurora A acting as a permissive licensing factor for AIM2, or does it represent an independent and equally critical regulatory layer? At present, the manuscript does not clearly distinguish between pathogen sensing (DNA-AIM2) and inflammasome execution (Aurora A-ASC), making it difficult to assess their respective importance. To resolve this issue, additional genetic controls in vivo are needed. In particular, inclusion of $Nlrp3^{-/-}$ mice in the infection model used in Figure 6 would allow a direct comparison between AIM2- and NLRP3-dependent contributions to disease, thereby clarifying whether the observed pathology is truly AIM2-dominant. Furthermore, analysis of $Asc^{-/-}$ mice would be highly informative to determine whether the in vivo phenotype attributed to AIM2 is strictly dependent on ASC-mediated inflammasome assembly, and to establish whether Aurora A-ASC regulation is indeed the central execution mechanism downstream of DNA sensing.

3 In Figure 7, the authors show that pharmacological inhibition of Aurora A reduces IL-1β and IL-18 production and markedly ameliorates S. aureus infection in vivo. While these results support Aurora A as a potential therapeutic target, they do not directly demonstrate that the protective effect is mediated through the ASC-inflammasome pathway proposed in the manuscript. Aurora A is a pleiotropic kinase with well-established roles in cell cycle regulation, cytoskeletal dynamics, and cellular stress responses, all of which could independently influence bacterial clearance and host survival. Therefore, the observed reduction in IL-1β and IL-18 could be secondary to improved bacterial control or altered cellular physiology, rather than a direct consequence of impaired ASC-dependent inflammasome activation. To establish a causal link between Aurora A inhibition and ASC-mediated inflammasome signaling, it would be important to include epistasis-type experiments, such as testing whether Aurora A inhibition provides additional protection in $Asc^{-/-}$ mice or ASC-deficient macrophages. If Aurora A acts primarily through ASC, Aurora inhibition should not further improve outcomes in the absence of ASC. Such experiments would be critical to validate the proposed Aurora A-ASC-inflammasome axis in vivo.

Referee #2:

Yamauchi et al. present interesting work on an unexpected activity of Aurora Kinase A in the control of the innate immune response by direct phosphorylation of the inflammasome adapter protein ASC. They demonstrate that this activity is downstream of JNK signalling and promotes ASC speck formation in both AIM2 and NLRP3 inflammasomes. They show that inhibition of Aurora Kinase A by known pharmacological agents markedly improves survival in mouse S. aureus infection models, suggesting a possible therapeutic strategy for antimicrobial-resistant S. aureus (MRSA), targeting the JNK-Aurora-ASC axis.

The association of Aurora kinase A with inflammation/inflammasomes is not novel. A cursory search yielded papers linking Aurora kinase A with the AIM2 inflammasome and with inflammation in general, for example, Tang, H., et al. (2021). "AURKA facilitates the psoriasis-related inflammation by impeding autophagy-mediated AIM2 inflammasome suppression." Immunol Lett 240: 98-105. The authors should note these links in their introduction. Of interest, though not necessarily worth noting is that one of the cellular locations of Aurora kinase A is at the centrosome, recently identified as the location for ASC speck formation (Wang, J., et al. (2024). "Human NLRP3 inflammasome activation leads to formation of condensate at the microtubule organizing center." bioRxiv: 2024.2009.2012.612739.) though this work has not yet been peer-reviewed.

Though the data are interesting and mostly well presented, the methods used are sometimes only described cursorily, making reproduction the work difficult and the figures are sometimes unclear and require revision.

Detailed comments:
Line

Introduction
85: "Recent studies... ". The cited papers are >10 years old, hardly "recent".

Results
118: Give a reference for the KinasePhos 3.0 platform and see comments on Fig. 1
130: " ...a series of ASC point mutants... ". What effect did these point mutations have on the formation of ASC specks and on their morphology?
148: What other proteins does Aurora kinase A phosphorylate - are any of these affected by the knockout and are any other phenotypic differences observed in these knockout macrophages?
150: "...TNF secretion was comparable..." See comments on Fig. 2.
167: "caspase-1 cleavage". The data presented are not convincing, see comments on Fig. 3.
192: "Figures S5A, B". These data should be presented in Fig. 5.
206: "...we assessed the presence of bacterial DNA using the DNA binding dye..." No method is given for this - see comments on Methods and Fig. 5.
275: I would expect some comment in the discussion on the conservation of the serine residues identified as targets for Aurora

kinase A, their location in the ASC sequence and any structural consequences of their phosphorylation.
287: A comment on the effects on other (non-AIM2/NLRP3) inflammasomes should be given.

Methods
341: Details of the Hoechst 33342 dye staining, especially with respect to quantitation of undigested/digested bacteria should be given.
403: Sufficient detail of the LC-MS/MS settings used to permit reproduction should be given. (e.g. LC column - the Q Exactive Plus itself is only the MS).
410: KinasePhos 3.0. Details of any settings should be given, otherwise 'default settings were used'.
460: BZ-X810 fluorescence microscopy - again, details of settings, filters, objectives, etc. are needed.

Figures
For multiple figures: move one of the set of results for the two strains of S. aureus to supplementary figures (Fig. 1 A, Fig. 4 A-E).
Be consistent with panel order - Alisertib before Tozasertib (or vice versa), Strain NUMR101 before 8325-4 (or vice versa) - and consistent with the text appearance of these.
Data point size is minuscule, even with zooming and would benefit from being increased.

Fig. 1
Panel D. This does not add much to the figure and the kinase notation (AurK) does not match the notations in Suppl. Fig. 1 (I presume Other_Aur?). I would suggest a separate table, giving the data in Fig. S1 B, with Aurora kinase A and it's score highlighted. I would also like to see the kinase data for Y144 - does this prediction match the known kinases for this residue? Also, given S105 is later identified as an Aurora kinase A target, does the prediction for this residue show any interesting associations? Is Aurora kinase A picked up by the prediction, and if not, why not?
Panel E. The layout of the labels on the left-hand side is confusing: from this, I expected five horizontal bands of data, rather than the three intended. Perhaps link the IP and IB lines with a dash, slash or similar and add a line between the label sections?

Fig. 2
Panel B, D. Why do these panels have different y-scales?
Panel H. Text (Line 150) says "TNF secretion was comparable", but this panel shows a difference. Please show the statistical significance of the difference here so that "comparable" can be judged (If not significant, show NS).
Panel J. Give a size for the scale bar.

Fig. 3
Panel C, D. Why is the measured IL-1 level lower for 0 SP in panel D than for untreated (DMSO) in Panel C?
Panel F. The suppression of Caspase-1 processing shown here is marginal at best and could simply be due to a variation in detection for the presented blot. A more convincing blot needs to be presented, or the blots need to be quantitated, and a statement of statistical significance attached to the quantitation to allow the degree of suppression to be judged.
Panel H, I. The previous figure presents Alisertib-treated before Tozasertib-treated; here it is the reverse. Panel order should be treated consistently.

Fig. 4
Panel A, C. WT IL-1b levels vary significantly between these panels. Line 179 says "NUMR101.. induced IL-1b... as well as... 8325-4", but these panels suggest it induces twice as well.
Panel G. Include significance or NS for WT vs Aim2-/-
Panel I. Scale bar size
Fig. S5 Panels A and B should be part of this figure.

Fig. 5
An explanation for the use of different time courses (Panel A - F: 6, 12, 18h, Panel G-H 0,1,3, 12 h) should be given and commented on.
Panel G, H. Include an explanation as to how digested and undigested bacterial were distinguished

Fig. 6
Panel F, G. Include significance or NS.

Fig. S1
See comments above on Panel B.

Fig. S3
For clarity, either colour each inhibitor set differently or include a larger space between each set or both. Give the concentrations of the inhibitors used.
Panel D. Why is the overall level of TNF significantly less than in Panel B?

Referee #3:

In this manuscript, the authors identify Aurora kinase A as a novel regulator of inflammasome activation during Staphylococcus aureus infection. They propose a JNK-Aurora A signaling axis leading to serine phosphorylation of ASC, enhanced ASC speck formation, and increased activation of the AIM2 and NLRP3 inflammasomes. Pharmacological inhibition of Aurora kinases is further shown to improve disease outcome in murine models of MRSA infection.

While the study addresses an interesting and timely question, the manuscript overstates both mechanistic specificity and therapeutic relevance, and several key conclusions are not fully supported by the data presented.

A central claim of the manuscript is that Aurora A specifically phosphorylates ASC to promote inflammasome activation. However, several inhibitors used throughout the study (e.g. Tozasertib) are known to target multiple Aurora kinases. Genetic approaches are limited and do not convincingly exclude contributions from Aurora B or C. Moreover, direct biochemical evidence demonstrating Aurora A-dependent phosphorylation of ASC remains limited. As currently presented, the data support a role for Aurora kinases in general, rather than unequivocally establishing Aurora A specificity.

The authors identify multiple serine residues (S100, S105) as potential Aurora A phosphorylation sites. The functional contribution of individual versus combined phosphorylation events is not systematically addressed, which weakens the mechanistic clarity of the proposed model.

The positioning of Aurora A downstream of JNK is inferred largely from inhibitor-based experiments. In addition, alternative pathways linking bacterial infection to Aurora kinase activation are not excluded. Consequently, the proposed linear JNK-Aurora A-ASC signaling axis remains incompletely substantiated.
The authors further suggest that Aurora kinase inhibition represents a promising host-directed therapeutic strategy for antibiotic-resistant S. aureus infection. However, the inhibitors used have broad cellular effects, notably on cell cycle regulation, and potential immunosuppressive or toxic consequences are not adequately discussed. The improved survival observed in vivo could therefore reflect indirect effects unrelated to inflammasome regulation. For these reasons, the therapeutic implications should be substantially toned down.

Additional Major and Specific Comments:
-The manuscript alternates between AIM2 and NLRP3 without clearly defining their respective contributions to in vivo pathology.
-Does Aurora A interact with ASC following S. aureus infection under endogenous conditions?

Figure-related comments:
• Figure 2E: In addition to cell extracts, cell supernatants should be analyzed by Western blot for detection of IL-1β p17 (and caspase-1 p20, as shown in Fig. 2I) to support the data presented in Fig. 2A. Moreover, caspase-1 p45 should be shown above caspase-1 p20, and IL-1β p31 above IL-1β p17. A non-infected control should be included to demonstrate the absence of caspase-1 p20 and IL-1β p17 in resting cells.
• Figure 2I: Why is caspase-1 p20 not detected in the cell extracts? Similarly, IL-1β p17 should be analyzed in both cell extracts and supernatants.
• Figures 2J and 2K: ASC specks appear difficult to detect as the percentage of speck-positive cells is low. Have the authors attempted to assess ASC oligomerization using DSS cross-linking?
• Figure 3: Does SP600125 treatment affect TNF-α production?
• Figure 3F: Both cell extracts and supernatants should be analyzed by Western blot for detection of pro- and cleaved forms of IL-1β and caspase-1. A non-infected control should be included. Similar comments apply to Figures 4D, 4E, and 4H, if feasible.
• Figure S5: Why were Aurora A-deficient BMDMs not used to directly assess the role of this kinase in AIM2 and NLRP3 inflammasome activation?
• Figure 6: Do Aurora A or JNK knockout mice phenocopy AIM2 knockout mice following S. aureus infection?
Minor Comments
• The Discussion should propose plausible mechanisms for JNK activation following S. aureus infection.
• The number of biological replicates is not always clearly indicated in figure legends.
• The rationale for using different MOIs (10, 25, or 50) should be clarified.

Conclusion:
While the manuscript addresses an interesting regulatory pathway and contains a considerable body of data, key mechanistic and conceptual claims are currently overstated. Substantial revision is required to improve specificity, mechanistic depth, and figure clarity.

We thank the reviewers for their positive and constructive comments. To address the points raised by the referees, we have conducted additional experiments and/or revised the text accordingly. The revised text is shown in red. The results of the new experiments are provided in new panels, specifically Figure 1 (D, H, I), Figure 2 (E, I), Figure 3 (F, J−L), Figure 4 (A−C, F− I), Figure 5 (D), and Figure 7 (G) as well as Figures EV1 (A, C), EV2, EV3, EV4 and Appendix Figure S1 (B), S2, S4, S5, S7, S9, and S10. Our point-to-point reply to the reviewers is provided below:

Point-by-Point Response

Referee #1:

1. The manuscript proposes a JNK-Aurora A signaling axis in S. aureus-induced inflammasome activation. While the authors convincingly show that JNK deficiency or pharmacological inhibition reduces Aurora A phosphorylation and downstream caspase-1 activation, the mechanistic link between JNK and Aurora A remains insufficiently defined. Specifically, it is unclear whether JNK directly phosphorylates Aurora A or whether Aurora A activation is regulated indirectly through intermediate kinases, phosphatases, or cofactors. The study does not provide biochemical evidence (e.g., in vitro kinase assays, binding assays, or phosphorylation-site analysis) to support a direct JNK-Aurora A interaction. Given that Aurora A activation is classically regulated by autophosphorylation and co-factors, it is important to clarify how JNK signaling mechanistically converges on Aurora A in this context. Without defining this molecular connection, the proposed JNK-Aurora A axis remains correlative rather than mechanistically established.

Reply: Thank you for this important comment. In fibroblasts and ovarian granulosa cells, JNK has been reported to promote entry into mitosis through phosphorylation of Aurora B (Oktay K et al. 2008, 7, 533-541). However, the immunological functions of Aurora kinases in macrophages have remained unclear. To examine whether p-JNK1 interacts with Aurora A under endogenous conditions, we performed a proximity ligation assay. The interaction between p-JNK1 and Aurora A was detected in response to *S. aureus* infection (Fig. 3J, K). In addition, to know whether JNK1 directly phosphorylates Aurora

A, we performed an in vitro kinase assay and observed a dose-dependent increase in Aurora A phosphorylation (Fig. 3L). These results indicate that JNK1 interacts with Aurora A and phosphorylates it. We have described this point in the text (Line195).

2. The manuscript proposes that Aurora A-mediated ASC phosphorylation is required for both AIM2 and NLRP3 inflammasome activation during S. aureus infection. At the same time, Figure 5 demonstrates that bacterial DNA is released into the cytosol of infected macrophages, and Figure 6 further shows that AIM2 deficiency alone markedly improves survival, reduces bacterial burden, and decreases IL-1β and IL-18 production in vivo. These data strongly suggest that DNA-driven AIM2 activation is a dominant pathogenic pathway in this infection model. This raises a conceptual ambiguity in the current model: if cytosolic bacterial DNA-AIM2 signaling is sufficient to drive inflammasome-dependent pathology in vivo, what is the relative contribution of Aurora A-mediated ASC phosphorylation? Is Aurora A acting as a permissive licensing factor for AIM2, or does it represent an independent and equally critical regulatory layer? At present, the manuscript does not clearly distinguish between pathogen sensing (DNA-AIM2) and inflammasome execution (Aurora A-ASC), making it difficult to assess their respective importance. To resolve this issue, additional genetic controls in vivo are needed. In particular, inclusion of Nlrp3$^{-/-}$ mice in the infection model used in Figure 6 would allow a direct comparison between AIM2- and NLRP3-dependent contributions to disease, thereby clarifying whether the observed pathology is truly AIM2-dominant. Furthermore, analysis of Asc$^{-/-}$ mice would be highly informative to determine whether the in vivo phenotype attributed to AIM2 is strictly dependent on ASC-mediated inflammasome assembly, and to establish whether Aurora A-ASC regulation is indeed the central execution mechanism downstream of DNA sensing.

Reply: Thank you for this valuable comment. Because the involvement of NLRP3 in *S. aureus* infection has already been reported in previous studies (Cohen et al. 2018, 22, 2431-2441; Kebaier et al. 2012, 205, 807-817), we mainly presented the results obtained from AIM2 KO mice in Fig. 6. To address this comment in the revised manuscript, we added *in vivo* data comparing NLRP3, AIM2, and ASC KO mice in Fig. EV3. Mice deficient for NLRP3 also showed less bacterial burdens in the livers and production of IL-1β and IL-18 after infection with *S. aureus*, with no significant differences between NLRP3 and AIM2 KO mice (Fig. EV3A–C). In ASC KO mice, inflammasome responses were more reduced than in NLRP3 or AIM2 KO mice (Fig. EV3A–C). These findings

suggest that NLRP3- and AIM2-mediated inflammasome responses cooperatively exacerbate the pathogenesis of *S. aureus* infection. In contrast, when mice were infected with a lower bacterial dose, the predominant contribution of AIM2 was observed (Fig. EV3D). Moreover, we transplanted Cas9- or *Aurka*$^{-/-}$-immortalized macrophages into ASC KO mice to assess the contribution of Aurora-mediated inflammasome responses. Compared with mice transplanted with Cas9 control cells, mice transplanted with *Aurka*$^{-/-}$ macrophages showed reduced IL-1β production and bacterial burdens in the organs (Fig. EV4). In addition, Tozasertib administration did not affect bacterial burdens in the livers of ASC KO mice (current Fig. 7G). These results indicate that Aurora signaling increases bacterial burdens in *S. aureus*-infected mice in an ASC-dependent manner. We have described these points in the text (Lines 271 and 294).

3. In Figure 7, the authors show that pharmacological inhibition of Aurora A reduces IL-1β and IL-18 production and markedly ameliorates S. aureus infection in vivo. While these results support Aurora A as a potential therapeutic target, they do not directly demonstrate that the protective effect is mediated through the ASC-inflammasome pathway proposed in the manuscript. Aurora A is a pleiotropic kinase with well-established roles in cell cycle regulation, cytoskeletal dynamics, and cellular stress responses, all of which could independently influence bacterial clearance and host survival. Therefore, the observed reduction in IL-1β and IL-18 could be secondary to improved bacterial control or altered cellular physiology, rather than a direct consequence of impaired ASC-dependent inflammasome activation. To establish a causal link between Aurora A inhibition and ASC-mediated inflammasome signaling, it would be important to include epistasis-type experiments, such as testing whether Aurora A inhibition provides additional protection in Asc$^{-/-}$ *mice or ASC-deficient macrophages. If Aurora A acts primarily through ASC, Aurora inhibition should not further improve outcomes in the absence of ASC. Such experiments would be critical to validate the proposed Aurora A-ASC-inflammasome axis in vivo.*

Reply: Thank you for this critical comment. To address this point, we confirmed that administration of Tozasertib to ASC-deficient mice did not reduce the bacterial burden during *S. aureus* infection (current Fig. 7G), indicating that Aurora signaling increases bacterial burdens in *S. aureus*-infected mice in an ASC-dependent manner.

Referee #2:

Yamauchi et al. present interesting work on an unexpected activity of Aurora Kinase A in the control of the innate immune response by direct phosphorylation of the inflammasome adapter protein ASC. They demonstrate that this activity is downstream of JNK signalling and promotes ASC speck formation in both AIM2 and NLRP3 inflammasomes. They show that inhibition of Aurora Kinase A by known pharmacological agents markedly improves survival in mouse S. aureus infection models, suggesting a possible therapeutic strategy for antimicrobial-resistant S. aureus (MRSA), targeting the JNK-Aurora-ASC axis.

The association of Aurora kinase A with inflammation/inflammasomes is not novel. A cursory search yielded papers linking Aurora kinase A with the AIM2 inflammasome and with inflammation in general, for example, Tang, H., et al. (2021). " facilitates the psoriasis-related inflammation by impeding autophagy-mediated AIM2 inflammasome suppression." Immunol Lett 240: 98-105. The authors should note these links in their introduction. Of interest, though not necessarily worth noting is that one of the cellular locations of Aurora kinase A is at the centrosome, recently identified as the location for ASC speck formation (Wang, J., et al. (2024). "Human NLRP3 inflammasome activation leads to formation of condensate at the microtubule organizing center." bioRxiv: 2024.2009.2012.612739.) though this work has not yet been peer-reviewed.

Though the data are interesting and mostly well presented, the methods used are sometimes only described cursorily, making reproduction the work difficult and the figures are sometimes unclear and require revision.

<u>Reply:</u> Thank you for introducing the two previously published papers. In the Introduction, we cited these studies and described the relationship among AIM2, Aurora A, and the centrosome (Line 92). We have revised the Methods and Figures, and our responses to each of the points you raised are described below.

Detailed comments:

Line

Introduction

85: "Recent studies... ". The cited papers are >10 years old, hardly "recent".

Reply: Thank you for pointing that out. I have corrected “recent” to “previous” (Line 86).

Results

118: Give a reference for the KinasePhos 3.0 platform and see comments on Fig. 1

Reply: In accordance with the referee’s comment, we have cited the reference for the KinasePhos 3.0 platform (Ma et al. 2023, 21, 228-241) (Line 121).

130: " ...a series of ASC point mutants... ". What effect did these point mutations have on the formation of ASC specks and on their morphology?

Reply: In response to this comment, we performed additional experiments and found that ASC speck formation was reduced in macrophages expressing the ASC S100A or S105A mutants (Fig. 1H, I and Line 135).

148: What other proteins does Aurora kinase A phosphorylate - are any of these affected by the knockout and are any other phenotypic differences observed in these knockout macrophages?

Reply: As it has been reported that Aurora A phosphorylates CDC25B in osteosarcoma cell line (Cazales et al, 2005, 4, 1233-1238; Dutertre et al, 2004, 117, 2523-2531), we examined the phosphorylation of CDC25B in our Aurora A-deficient macrophages. We found that CDC25B is expressed in macrophages before infection, and its expression level was comparable between control macrophages and Aurora A knockout cells (Fig. EV2D). In addition, although *S. aureus* infection induced CDC25B phosphorylation, a similar level of phosphorylation was also observed in Aurora A knockout macrophages (Fig. EV2D). These findings suggest that CDC25B phosphorylation induced by *S. aureus* infection occurs independently of Aurora A in macrophages. Thus, Aurora A appears to regulate inflammasome activation through a mechanism distinct from CDC25B phosphorylation in this context. We have described this point in the text (Line 164).

150: "...TNFα secretion was comparable..." See comments on Fig. 2.

Reply: Thank you for your comment. We have added a statistical analysis result for TNFα and revised the text accordingly (Fig. 2H and Line 158).

167: "caspase-1 cleavage". The data presented are not convincing, see comments on Fig. 3.

Reply: We apologize that the reduction in caspase-1 processing in Mapk8-deficient macrophages was unclear. To address this issue, we performed additional experiments, and the results are shown in Fig. 3F.

192: "Figures S5A, B". These data should be presented in Fig. 5.

Reply: In accordance with this comment and the comment in the Figure 4 section below, we have moved these data to current Fig. 4F, G.

206: "...we assessed the presence of bacterial DNA using the DNA binding dye..." No method is given for this - see comments on Methods and Fig. 5.

Reply: Thank you for your constructive comment. We have revised the Methods section to provide a more detailed description, cited the reference paper we followed (Lines 241 and 560), and added representative images to Fig. 5D.

275: I would expect some comment in the discussion on the conservation of the serine residues identified as targets for Aurora kinase A, their location in the ASC sequence and any structural consequences of their phosphorylation.

Reply: As shown in Appendix Fig. S9, we examined the conservation of the Aurora A target residues ASC S100 and S105 across nine animal species and found that each serine residue was conserved in approximately 60% of the species. Moreover, this alignment shows that serine and threonine residues that can serve as phosphorylation targets are clustered within this region of ASC, raising the possibility that Aurora A may also phosphorylate other amino acid residues in a species-dependent manner. We discussed this point in Line 332.

287: A comment on the effects on other (non-AIM2/NLRP3) inflammasomes should be given.

Reply: Thank you for your helpful comment. We have described that inflammasomes other than NLRP3 and AIM2 also contribute to the exacerbation of *S. aureus* infection (Line 357).

Methods

341: Details of the Hoechst 33342 dye staining, especially with respect to quantitation of undigested/digested bacteria should be given.

Reply: Thank you for your constructive comment. We have revised the Methods section to provide a more detailed description, cited the reference paper we followed (Lines 241 and 560), and added representative images to Fig. 5D.

403: Sufficient detail of the LC-MS/MS settings used to permit reproduction should be given. (e.g. LC column - the Q Exactive Plus itself is only the MS).

Reply: In accordance with the comment, we have provided detailed LC-MS/MS settings (Line 464).

410: KinasePhos 3.0. Details of any settings should be given, otherwise 'default settings were used'.

Reply: We have added a description of the KinasePhos 3.0 settings (Line 471).

460: BZ-X810 fluorescence microscopy - again, details of settings, filters, objectives, etc. are needed.

Reply: As suggested, we have added the microscope settings to the Methods section (Line 527).

Figures

For multiple figures: move one of the set of results for the two strains of S. aureus to supplementary figures (Fig. 1 A, Fig. 4 A-E).

Reply: In accordance with the comment, we have moved previous Fig. 1A to current Fig. EV1A, previous Fig. 4C, E to current Appendix Fig. S5, and previous Fig. 5D–F to current Appendix Fig. S7.

Be consistent with panel order - Alisertib before Tozasertib (or vice versa), Strain NUMR101 before 8325-4 (or vice versa) - and consistent with the text appearance of these.

Reply: We standardized the panel order as 8325-4 then NUMR101, and Alisertib then Tozasertib.

Data point size is minuscule, even with zooming and would benefit from being increased.

Reply: In accordance with the comment, we have increased the data point size.

Fig. 1

Panel D. This does not add much to the figure and the kinase notation (AurK) does not match the notations in Suppl. Fig. 1 (I presume Other_Aur?). I would suggest a separate table, giving the data in Fig. S1 B, with Aurora kinase A and it's score highlighted. I would also like to see the kinase data for Y144 - does this prediction match the known kinases for this residue? Also, given S105 is later identified as an Aurora kinase A target, does the prediction for this residue show any interesting associations? Is Aurora kinase A picked up by the prediction, and if not, why not?

Reply: Thank you for your constructive comment. In accordance with the comment, we modified current Fig. EV1C and added the information of ASC S105 and Y144. Syk, which has previously been reported to phosphorylate ASC Y144, was identified as a candidate kinase. In contrast, Aurora kinase was not predicted as a candidate for ASC S105. Based on previous reports (Ferrari et al, 2005, 390, 293-302), it is possible that KinasePhos 3.0 was programmed to preferentially recognize Aurora A substrates in which a hydrophobic amino acid is located immediately C-terminal to the serine residue, which may explain why S100 was preferentially identified. Using these predictions as a reference, we performed functional analyses in macrophages and were thereby able to identify ASC S105 as an additional functionally relevant site. We described this point in the text (Line 326).

Panel E. The layout of the labels on the left-hand side is confusing: from this, I expected five horizontal bands of data, rather than the three intended. Perhaps link the IP and IB lines with a dash, slash or similar and add a line between the label sections?

Reply: We apologize for the confusion. We have corrected the layout to clearly show three columns in current Fig. 1D.

Fig. 2

Panel B, D. Why do these panels have different y-scales?

Reply: As suggested, we unified the y-scales in Fig. 2B, D.

Panel H. Text (Line 150) says "TNFα secretion was comparable", but this panel shows a difference. Please show the statistical significance of the difference here so that "comparable" can be judged (If not significant, show NS).

Reply: Thank you for your comment. We have added a statistical analysis result for TNFα and revised the text accordingly (Fig. 2H and Line 158).

Panel J. Give a size for the scale bar.

Reply: Thank you for pointing out this oversight. We have added the scale bar size to the figure legend.

Fig. 3

Panel C, D. Why is the measured IL-1β level lower for 0 SP in panel D than for untreated (DMSO) in Panel C?

Reply: The difference may be attributable to the fact that panels C and D were performed at different facilities. Reperforming the experiment shown in panel D yielded values that were closer to those observed in panel C.

Panel F. The suppression of Caspase-1 processing shown here is marginal at best and could simply be due to a variation in detection for the presented blot. A more convincing

blot needs to be presented, or the blots need to be quantitated, and a statement of statistical significance attached to the quantitation to allow the degree of suppression to be judged.

Reply: We apologize that the reduction in caspase-1 processing in Mapk8-deficient macrophages was unclear. To address this issue, we performed additional experiments, and the results are shown in Fig. 3F.

Panel H, I. The previous figure presents Alisertib-treated before Tozasertib-treated; here it is the reverse. Panel order should be treated consistently.

Reply: Thank you for your helpful comment. We revised the panel order in Fig. 3H, I to make it consistent.

Fig. 4

Panel A, C. WT IL-1b levels vary significantly between these panels. Line 179 says "NUMR101.. induced IL-1b... as well as... 8325-4", but these panels suggest it induces twice as well.

Reply: We revised the text (Line 209).

Panel G. Include significance or NS for WT vs Aim2-/-

Reply: Thank you for your comment. Because we were able to obtain primary NLRP3-deficient BMDMs, we additionally infected primary WT, NLRP3-deficient, and AIM2-deficient BMDMs with *S. aureus* and replaced the previous data shown in Fig. 4A–H. These results, together with statistical analyses, are now included in the current Fig. 4A–C and Appendix Fig. S5.

Panel I. Scale bar size

Reply: Thank you for pointing out this oversight. We have added the scale bar size to the figure legend.

Fig. S5 Panels A and B should be part of this figure.

Reply: In accordance with the comment, we have moved the data to current Fig. 4F, G.

Fig. 5
An explanation for the use of different time courses (Panel A - F: 6, 12, 18h, Panel G-H 0,1,3, 12 h) should be given and commented on.

Reply: Thank you for your helpful comment. As suggested, we have added an explanation for why the experiments were performed at different time points (Line 242).

Panel G, H. Include an explanation as to how digested and undigested bacterial were distinguished.
Reply: Thank you for your constructive comment. We have revised the Methods section to provide a more detailed description, cited the reference paper we followed (Lines 241 and 560), and added representative images to current Fig. 5D.

Fig. 6
Panel F, G. Include significance or NS.

Reply: In accordance with the comment, we have included statistical analysis results in Fig. 6F, G.

Fig. S1
See comments above on Panel B.

Reply: We have described our response in the Fig. 1 section above.

Fig. S3
For clarity, either colour each inhibitor set differently or include a larger space between each set or both. Give the concentrations of the inhibitors used.

Reply: Thank you for your comment. To improve visual clarity, we color-coded each inhibitor throughout the figure.

Panel D. Why is the overall level of TNFα significantly less than in Panel B?
Reply: The difference may be attributable to the fact that panels B and D were performed at different facilities. We reanalyzed the panel B samples, the resulting values were closer

to those observed in panel D.

Referee #3:

In this manuscript, the authors identify Aurora kinase A as a novel regulator of inflammasome activation during Staphylococcus aureus infection. They propose a JNK-Aurora A signaling axis leading to serine phosphorylation of ASC, enhanced ASC speck formation, and increased activation of the AIM2 and NLRP3 inflammasomes. Pharmacological inhibition of Aurora kinases is further shown to improve disease outcome in murine models of MRSA infection.

While the study addresses an interesting and timely question, the manuscript overstates both mechanistic specificity and therapeutic relevance, and several key conclusions are not fully supported by the data presented.

A central claim of the manuscript is that Aurora A specifically phosphorylates ASC to promote inflammasome activation. However, several inhibitors used throughout the study (e.g. Tozasertib) are known to target multiple Aurora kinases. Genetic approaches are limited and do not convincingly exclude contributions from Aurora B or C. Moreover, direct biochemical evidence demonstrating Aurora A-dependent phosphorylation of ASC remains limited. As currently presented, the data support a role for Aurora kinases in general, rather than unequivocally establishing Aurora A specificity.

Reply: To reflect the involvement of Aurora kinases in general, we revised the Title from "Aurora Kinase A" to "Aurora Kinases". We also revised the descriptions in the main text to avoid restricting them specifically to Aurora A (Lines 50, 321, and 388). We have also added a statement discussing the possible involvement of Aurora B and C (Line 341).

The authors identify multiple serine residues (S100, S105) as potential Aurora A phosphorylation sites. The functional contribution of individual versus combined phosphorylation events is not systematically addressed, which weakens the mechanistic clarity of the proposed model.

Reply: Thank you for this important comment. To examine potential redundancy between these serine phosphorylation sites, we generated macrophages expressing an ASC S100/105A double mutant. Compared with ASC S100A single mutant, the ASC double mutant showed a further reduction in IL-1β production, suggesting that phosphorylation of S100 and S105 in ASC additively contributes to inflammasome activation (Appendix Fig. S1B). These results support the idea that both residues participate in Aurora A-mediated regulation of inflammasome activation.

The positioning of Aurora A downstream of JNK is inferred largely from inhibitor-based experiments. In addition, alternative pathways linking bacterial infection to Aurora kinase activation are not excluded. Consequently, the proposed linear JNK-Aurora A-ASC signaling axis remains incompletely substantiated.

Reply: In Fig. 3E, we have shown that phosphorylation of Aurora A is reduced in *Mapk8* knockout macrophages, suggesting that JNK is upstream of Aurora A. We now additionally observed the direct interaction between JNK1 and Aurora A in Fig. 3J–L. We also have detected phosphorylation of ASC by Aurora A using mass spectrometry in Fig. 1E, and additionally observed the interaction of Aurora A and ASC by in situ PLA (Fig. EV2B,C). These results indicate that JNK-Aurora-ASC axis controls inflammasome activation (Appendix Fig. S10). We also discussed the possibility that Aurora A may be regulated by kinases other than JNK (Line 353).

The authors further suggest that Aurora kinase inhibition represents a promising host-directed therapeutic strategy for antibiotic-resistant S. aureus infection. However, the inhibitors used have broad cellular effects, notably on cell cycle regulation, and potential immunosuppressive or toxic consequences are not adequately discussed. The improved survival observed in vivo could therefore reflect indirect effects unrelated to inflammasome regulation. For these reasons, the therapeutic implications should be substantially toned down.

Reply: We have added a discussion addressing this point (Line 388) and toned down the statements regarding the therapeutic implications throughout the manuscript.

Additional Major and Specific Comments:

-The manuscript alternates between AIM2 and NLRP3 without clearly defining their

respective contributions to in vivo pathology.

Reply: Thank you for your helpful comment. We evaluated the impact of NLRP3 and AIM2 on the pathogenesis of *S. aureus* infection using each deficient mice and revised the text (Fig. EV3 and Line 271).

-Does Aurora A interact with ASC following S. aureus infection under endogenous conditions?

Reply: To examine this point, we performed a proximity ligation assay for Aurora A and ASC. The PLA signals were increased after infection with *S. aureus*, indicating that Aurora A interacts with ASC in macrophages infected with *S. aureus*. (Fig. EV2B, C).

Figure-related comments:

• Figure 2E: In addition to cell extracts, cell supernatants should be analyzed by Western blot for detection of IL-1β p17 (and caspase-1 p20, as shown in Fig. 2I) to support the data presented in Fig. 2A. Moreover, caspase-1 p45 should be shown above caspase-1 p20, and IL-1β p31 above IL-1β p17. A non-infected control should be included to demonstrate the absence of caspase-1 p20 and IL-1β p17 in resting cells.

Reply: In response to the reviewer's comment, we performed additional experiments, and the results are shown in Fig. 2E.

• Figure 2I: Why is caspase-1 p20 not detected in the cell extracts? Similarly, IL-1β p17 should be analyzed in both cell extracts and supernatants.

Reply: In response to the reviewer's comment, we performed additional experiments to detect caspase-1 p20 in the cell extracts and IL-1β p17, and the results are shown in Fig. 2I.

• Figures 2J and 2K: ASC specks appear difficult to detect as the percentage of speck-positive cells is low. Have the authors attempted to assess ASC oligomerization using DSS cross-linking?

Reply: Thank you for your constructive comment. We attempted to detect ASC oligomers by DSS crosslinking. In Cas9 macrophages infected with *S. aureus*, ASC was detected as

a high-molecular-weight band (Fig. EV2A), consistent with the immunostaining results.

• *Figure 3: Does SP600125 treatment affect TNF-α production?*

Reply: To address this question, we measured TNFα and found that its levels were partially reduced following inhibitor treatment (Appendix Fig. S4). As *S. aureus* has been reported to activate JNK via Toll-like receptor 2, leading to NF-κB activation (Fang et al, 2014, 26, 806-814; Watanabe et al, 2007, 178, 4917-4925), we mentioned this point in the text (Lines 183 and 345).

• *Figure 3F: Both cell extracts and supernatants should be analyzed by Western blot for detection of pro- and cleaved forms of IL-1β and caspase-1. A non-infected control should be included. Similar comments apply to Figures 4D, 4E, and 4H, if feasible.*

Reply: In response to the reviewer's comment, we performed additional experiments, and the results are shown in current Fig. 3F, 4C, and Appendix Fig. S5C. Because we were able to obtain primary NLRP3-deficient BMDMs, we additionally infected primary WT, NLRP3-deficient, and AIM2-deficient BMDMs with *S. aureus* and replaced the previous data shown in Figure 4A–H. These results are now included in the current Fig. 4A–C and Appendix Fig. S5.

• *Figure S5: Why were Aurora A-deficient BMDMs not used to directly assess the role of this kinase in AIM2 and NLRP3 inflammasome activation?*

Reply: Thank you for this important comment. We performed additional experiments using Aurora A-deficient macrophages, and the results are presented in current Fig. 4H, I.

• *Figure 6: Do Aurora A or JNK knockout mice phenocopy AIM2 knockout mice following S. aureus infection?*

Reply: Because *Aurka*$^{-/-}$ mice are embryonically lethal (Lu et al. 2008, 283, 31785-31790) and JNK1-deficient mice exhibit immunodeficiency and developmental defects (Dong et al. 1998, 282, 2092-2095; Chang et al. 2003, 4, 521-533), we did not use these mice for the *in vivo* infection experiments. Instead, to address this question, we transplanted Cas9-

or *Aurka*$^{-/-}$-immortalized macrophages into *Pycard*$^{-/-}$ mice to assess the impact of Aurora-mediated inflammasome responses. Compared with mice transplanted with Cas9 control cells, mice transplanted with *Aurka*$^{-/-}$ macrophages showed reduced bacterial burdens in the organs (Fig. EV4).

Minor Comments

• The Discussion should propose plausible mechanisms for JNK activation following S. aureus infection.

Reply: As *S. aureus* has been reported to activate JNK via Toll-like receptor 2, leading to NF-κB activation (Fang et al, 2014, 26, 806-814; Watanabe et al, 2007, 178, 4917-4925), we mentioned this point in the text (Lines 183 and 345).

• The number of biological replicates is not always clearly indicated in figure legends.

Reply: Thank you for your comment. We have included the number of biological replicates in the figure legends.

• The rationale for using different MOIs (10, 25, or 50) should be clarified.

Reply: Thank you for your comment. We have described the rationale for the use of different MOIs in the Methods section (Line 485).

Dear Prof. Hara,

Thank you for the submission of your revised manuscript to our editorial offices. I have now received the reports from two of the three referees that were asked to re-evaluate the study, you will find below. Original referee #1 declined to look into the revision. Nevertheless, going through your p-b-p-response, I consider his/her points as adequately addressed. As you will see, the other two referees now fully support publication of your study in EMBO reports.

Before I can proceed with formal acceptance, I have the editorial requests below I ask you to address in a final revised manuscript. Please also provide a final p-b-p-response to the editorial requests.

Editorial requests:

- I would suggest this modified title:
Aurora A exacerbates antibiotic-resistant staphylococcus aureus infection through ASC phosphorylation

- Please order the manuscript sections like this, using only these names:
Title page - Abstract - Keywords (max. 5) - Introduction - Results - Discussion - Methods - Data availability section - Acknowledgements - Disclosure and Competing Interests Statement - References - Figure legends - Expanded View Figure legends

- We now use CRediT to specify the contributions of each author in the journal submission system. CRediT replaces the author contribution section. Please use the free text box to provide more detailed descriptions and do NOT provide your final manuscript text file with an author contributions section. See also our guide to authors (section 'Author contributions'): https://link.springer.com/journal/44319/submission-guidelines#cms-Revised-submissions

- Please note that all corresponding authors are required to supply an ORCID ID for their name upon submission of a revised manuscript. We will not proceed with publication if this is not done. The ORCID of co-cor.-authors Kei Sakamoto is still missing. The ORCID ID needs to be linked to the author account in our manuscript tracking system. This can only be done by the author.

- The data availability section (DAS) is restricted to information about large datasets generated in the study that have been deposited externally. Please remove any other information from this section. I no datasets have been deposited, just state this here ('No primary datasets have been generated and deposited for this study').

- Please check again that the number "n" for how many independent experiments were performed, their nature (biological versus technical replicates), the bars and error bars (e.g. SEM, SD) and the test used to calculate p-values is indicated in the respective figure legends (main, EV Figures and Appendix Figures). Please also check that all the p-values are explained in the legend, and that these fit to those shown in the figure. Please provide statistical testing where applicable. Please avoid the phrase 'independent experiment' but clearly state if these were biological or technical replicates. Please also indicate (e.g. with n.s.) if testing was performed, but the differences are not significant. In case n=2, please show the data as separate datapoints without error bars and statistics. See also:

https://link.springer.com/journal/44319/submission-guidelines#cms-Figure-and-data-presentation

If n<5, please show single datapoints for diagrams. A few panels seem to lack statistics (e.g. 5B/C, S7B/C and S2). Please make also sure that 'n.s.' is always indicated. Moreover:
- For many panels you are not clear if n=2 (it is soften stated 'three or two biological replicates' - e.g. for 3L; 4H,I; 6D-F; 7E,F; EV-2C; EV-3B,C; EV-4A-C; EV-5B-D). Please clearly indicate which panels show n=2 and in these cases please remove the statistics and show two separate datapoints (see above). Or add a third replicate.
- Please note that the measure of center for the error bars needs to be defined in the legends of figures 1A,G,I; 2A-D,G,H,K; 3C,D,G,K; 4A,B,F,G; 5A-C; EV-1A.
- Please note that the legends for figures 3, 6, EV-3 are not provided in a sequential manner (legend for figure 3H is provided before legend of figure 3C; legend for figure 6H is provided before legend of figure 6D; legend for figure EV-3D is provided before legend of figure EV-3B). This needs to be rectified.
- Please note that the exact p values are not provided in the legends of figures 1A,I; 2A,C,G,K; 3C,D,G; 4A,E,F,G; 5A; EV-1A.

- Please make sure that all the funding information is also entered into the online submission system and that it is complete and similar to the one in the acknowledgement section of the manuscript text file. Presently, these grants are missing in the system: TERUMO Life Science Foundation, The Nakajima Foundation, SHIONOGI Infectious Disease Research Promotion Foundation. Please check.

- Please provide a title page for the Appendix file (Appendix for: "title of the paper" - do not list again the authors and their affiliations) containing a table of contents, listing all the items and the respective page numbers. Each figure legend should follow

its figure and the text 'SUPPLEMENTALY FIGURE LEGENDS' needs to be removed. The nomenclature of the figure titles needs to be the same as the callouts in the manuscript (Appendix Fig. Sx). Finally, Appendix PDF needs to be clean (without track changes, highlighting, coloured text etc.).

- Please confirm that for all Western blot panels shown the loading control was run on the same gel as the other proteins detected. Please note that we discourage comparisons between samples on different gels/blots, even if the samples derive from one experiment, as confounding factors reduce comparability. If unavoidable, the figure legend must state that the samples derive from the same experiment and that gels/blots were processed in parallel. If a 'representative' loading control is shown for multiple gels/blots, the intra-gel controls should be shown in the source data files, and the figure legends should describe the data displayed accurately. See our author guidelines:

https://link.springer.com/journal/44319/submission-guidelines#cms-Figure-and-data-presentation (section 'Electrophoretic gels and blots').

- Thanks for providing the source data. Please upload the source data as one ZIPed folder per figure, containing all the source data for this figure. The excel files in each figure folder (numerical data) should be named with the panels it contains (e.g. Fig 1ABGI).

- Please remove the instructions text from the Reagents & Tools table.

In addition, I would need from you uploaded separately:
- a short, two-sentence summary of the manuscript (not more than 35 words).
- two to four short (!) bullet points highlighting the key findings of your study (two lines each).
- a schematic summary figure as separate file that provides a sketch of the major findings (not a data image) in jpeg or tiff format (with the exact width of 550 pixels and the exact height of 300 pixels) that can be used as a visual synopsis on our website. Please upload this separately and remove the graphical abstract from the manuscript text file.

I look forward to seeing the further revised version of your manuscript when it is ready. Please let me know if you have questions regarding the revision.

Best,

Achim Breiling
Senior Editor
EMBO Reports

Referee #2:

The authors have made the appropriate changes to the manuscript and have answered all the questions from the reviewers correctly. I recommend publication at this point

----
Referee #3:

The authors have carefully revised the manuscript and have satisfactorily addressed my previous comments. The revised version is clearly strengthened by the additional experiments, which provide substantially stronger mechanistic support for the proposed model. In particular, the new data clarifying the relationship between JNK, Aurora A and ASC, as well as the additional in vivo experiments addressing the respective contributions of AIM2, NLRP3 and ASC, significantly reinforce the study. The discussion has also been improved and the therapeutic implications are now presented more cautiously. Overall, I believe that the revised manuscript now supports its main conclusions and is suitable for publication.

Point-by-Point Response

Referee #2:

The authors have made the appropriate changes to the manuscript and have answered all the questions from the reviewers correctly. I recommend publication at this point

Reply: Thank you very much for your positive evaluation of our revised manuscript and for recommending it for publication.

Referee #3:

The authors have carefully revised the manuscript and have satisfactorily addressed my previous comments. The revised version is clearly strengthened by the additional experiments, which provide substantially stronger mechanistic support for the proposed model. In particular, the new data clarifying the relationship between JNK, Aurora A and ASC, as well as the additional in vivo experiments addressing the respective contributions of AIM2, NLRP3 and ASC, significantly reinforce the study. The discussion has also been improved and the therapeutic implications are now presented more cautiously. Overall, I believe that the revised manuscript now supports its main conclusions and is suitable for publication.

Reply: Thank you very much for your careful evaluation of our revised manuscript and for your positive comments. We are grateful that you consider the revised manuscript suitable for publication.

Prof. Hideki Hara
Asahikawa Medical University
Japan

Dear Prof. Hara,

I am very pleased to accept your manuscript for publication in the next available issue of EMBO reports. Thank you for your contribution to our journal.

Your manuscript will be processed for publication by EMBO Press. It will be copy edited and you will receive page proofs prior to publication. Please note that you will be contacted by Springer Nature Author Services to complete licensing and payment information.

You may qualify for financial assistance for your publication charges - either via a Springer Nature fully open access agreement or an EMBO initiative. Check your eligibility: https://link.springer.com/journal/44319/how-to-publish-with-us

Should you be planning a Press Release on your article, please notify the production team via this form in order to coordinate publication and release dates: https://forms.cloud.microsoft/e/eH4A8JVP6g

If you have any questions, please do not hesitate to contact the Editorial Office. Thank you for your contribution to EMBO Reports.

Yours sincerely,

Achim Breiling
Senior Editor
EMBO Reports

>>> Please note that it is EMBO Reports policy for the transcript of the editorial process (containing referee reports and your response letter) to be published as an online supplement to each paper. If you do NOT want this, you will need to inform the Editorial Office via email immediately. More information is available here: https://link.springer.com/partners/embo-press/editorial-policies#Peer%20review

EMBO Press Author Checklist

Corresponding Author Name: Hideki Hara	
Journal Submitted to: EMBO reports	
Manuscript Number: EMBOR-2025-63319V2	

USEFUL LINKS FOR COMPLETING THIS FORM
The EMBO Journal - Author Guidelines
EMBO Reports - Author Guidelines
Molecular Systems Biology - Author Guidelines
EMBO Molecular Medicine - Author Guidelines

Reporting Checklist for Life Science Articles (updated January
This checklist is adapted from Materials Design Analysis Reporting (MDAR) Checklist for Authors. MDAR establishes a minimum set of requirements in transparent reporting in the life sciences (see Statement of Task: 10.31222/osf.io/9sm4x). Please follow the journal's guidelines in preparing your
Please note that a copy of this checklist will be published alongside your article.

Abridged guidelines for figures
1. Data
The data shown in figures should satisfy the following conditions:
- the data were obtained and processed according to the field's best practice and are presented to reflect the results of the experiments in an accurate and unbiased manner.
- ideally, figure panels should include only measurements that are directly comparable to each other and obtained with the same assay.
- plots include clearly labeled error bars for independent experiments and sample sizes. Unless justified, error bars should not be shown for technical
- if n<5, the individual data points from each experiment should be plotted. Any statistical test employed should be justified.
- Source Data should be included to report the data underlying figures according to the guidelines set out in the authorship guidelines on Data

2. Captions
Each figure caption should contain the following information, for each panel where they are relevant:
- a specification of the experimental system investigated (eg cell line, species name).
- the assay(s) and method(s) used to carry out the reported observations and measurements.
- an explicit mention of the biological and chemical entity(ies) that are being measured.
- an explicit mention of the biological and chemical entity(ies) that are altered/varied/perturbed in a controlled manner.
- the exact sample size (n) for each experimental group/condition, given as a number, not a range;
- a description of the sample collection allowing the reader to understand whether the samples represent technical or biological replicates (including how many animals, litters, cultures, etc.).
- a statement of how many times the experiment shown was independently replicated in the laboratory.
- definitions of statistical methods and measures:
  - common tests, such as t-test (please specify whether paired vs. unpaired), simple χ2 tests, Wilcoxon and Mann-Whitney tests, can be unambiguously identified by name only, but more complex techniques should be described in the methods section;
  - are tests one-sided or two-sided?
  - are there adjustments for multiple comparisons?
  - exact statistical test results, e.g., P values = x but not P values < x;
  - definition of 'center values' as median or average;
  - definition of error bars as s.d. or s.e.m.

Please complete ALL of the questions below.
Select "Not Applicable" only when the requested information is not relevant for your study.

Materials

Newly Created Materials	Information included in the manuscript?	In which section is the information available? (Reagents and Tools Table, Materials and Methods, Figures, Data Availability Section)
New materials and reagents need to be available; do any restrictions apply?	Yes	Reagents and Tools Table

Antibodies	Information included in the manuscript?	In which section is the information available? (Reagents and Tools Table, Materials and Methods, Figures, Data Availability Section)
For **antibodies** provide the following information:<br>- Commercial antibodies: RRID (if possible) or supplier name, catalogue number and or/clone number<br>- Non-commercial: RRID or citation	Yes	Reagents and Tools Table

DNA and RNA sequences	Information included in the manuscript?	In which section is the information available? (Reagents and Tools Table, Materials and Methods, Figures, Data Availability Section)
**Short novel DNA or RNA including primers, probes**: provide the sequences.	Yes	Reagents and Tools Table

Cell materials	Information included in the manuscript?	In which section is the information available? (Reagents and Tools Table, Materials and Methods, Figures, Data Availability Section)
**Cell lines:** Provide species information, strain. Provide accession number in repository **OR** supplier name, catalog number, clone number, and/**OR** RRID.	Yes	Reagents and Tools Table
**Primary cultures:** Provide species, strain, sex of origin, genetic modification status.	Yes	Methods
Report if the cell lines were recently **authenticated** (e.g., by STR profiling) and tested for mycoplasma contamination.	Yes	Methods

Experimental animals	Information included in the manuscript?	In which section is the information available? (Reagents and Tools Table, Materials and Methods, Figures, Data Availability Section)
**Laboratory animals or Model organisms:** Provide species, strain, sex, age, genetic modification status. Provide accession number in repository **OR** supplier name, catalog number, clone number, **OR** RRID.	Yes	Reagents and Tools Table
**Animal observed in or captured from the field:** Provide species, sex, and age where possible.	Not Applicable	
Please detail **housing and husbandry conditions**.	Yes	Methods

Plants and microbes	Information included in the manuscript?	In which section is the information available? (Reagents and Tools Table, Materials and Methods, Figures, Data Availability Section)
**Plants:** provide species and strain, ecotype and cultivar where relevant, unique accession number if available, and source (including location for collected wild specimens).	Not Applicable	
**Microbes:** provide species and strain, unique accession number if available, and source.	Yes	Reagents and Tools Table

Human research participants	Information included in the manuscript?	In which section is the information available? (Reagents and Tools Table, Materials and Methods, Figures, Data Availability Section)
If collected and within the bounds of privacy constraints report on age, sex and gender or ethnicity for all study participants.	Not Applicable	

Core facilities	Information included in the manuscript?	In which section is the information available? (Reagents and Tools Table, Materials and Methods, Figures, Data Availability Section)
If your work benefited from core facilities, was their service mentioned in the acknowledgments section?	Yes	Acknowledgements

Design

Study protocol	Information included in the manuscript?	In which section is the information available? (Reagents and Tools Table, Materials and Methods, Figures, Data Availability Section)
If study protocol has been **pre-registered, provide DOI in the manuscript**. For clinical trials, provide the trial registration number **OR** cite DOI.	Not Applicable	
Report the **clinical trial registration number** (at ClinicalTrials.gov or equivalent), where applicable.	Not Applicable	

Laboratory protocol	Information included in the manuscript?	In which section is the information available? (Reagents and Tools Table, Materials and Methods, Figures, Data Availability Section)
Provide DOI OR other citation details if **external detailed step-by-step protocols** are available.	Not Applicable	

Experimental study design and statistics	Information included in the manuscript?	In which section is the information available? (Reagents and Tools Table, Materials and Methods, Figures, Data Availability Section)
Include a statement about **sample size** estimate even if no statistical methods were used.	Yes	Methods
Were any steps taken to minimize the effects of subjective bias when allocating animals/samples to treatment (e.g. **randomization procedure**)? If yes, have they been described?	Yes	Methods
Include a statement about **blinding** even if no blinding was done.	Yes	Methods
Describe **inclusion/exclusion criteria** if samples or animals were excluded from the analysis. Were the criteria pre-established?<br><br>If sample or data points were omitted from analysis, report if this was due to attrition or intentional exclusion and provide justification.	Not Applicable	
For every figure, are **statistical tests** justified as appropriate? Do the data meet the assumptions of the tests (e.g., normal distribution)? Describe any methods used to assess it. Is there an estimate of variation within each group of data? **I**s the variance similar between the groups that are being statistically compared?	Yes	Methods

Sample definition and in-laboratory replication	Information included in the manuscript?	In which section is the information available? (Reagents and Tools Table, Materials and Methods, Figures, Data Availability Section)
In the figure legends: state number of times the experiment was **replicated** in laboratory.	Yes	Figure legends
In the figure legends: define whether data describe **technical or biological replicates**.	Yes	Figure legends

Ethics

Ethics	Information included in the manuscript?	In which section is the information available? (Reagents and Tools Table, Materials and Methods, Figures, Data Availability Section)
Studies involving **human participants**: State details of **authority granting ethics approval** (IRB or equivalent committee(s), provide reference number for approval.	Not Applicable	
Studies involving **human participants**: Include a statement confirming that **informed consent** was obtained from all subjects and that the experiments conformed to the principles set out in the WMA Declaration of Helsinki and the Department of Health and Human Services Belmont Report.	Not Applicable	
Studies involving **human participants:** For publication of **patient photos**, include a statement confirming that consent to publish was obtained.	Not Applicable	
Studies involving experimental **animals**: State details of **authority granting ethics approval** (IRB or equivalent committee(s), provide reference number for approval. Include a statement of compliance with ethical regulations.	Yes	Methods
Studies involving **specimen and field samples:** State if relevant **permits** obtained, provide details of authority approving study; if none were required, explain why.	Not Applicable	

Dual Use Research of Concern (DURC)	Information included in the manuscript?	In which section is the information available? (Reagents and Tools Table, Materials and Methods, Figures, Data Availability Section)
Could your study fall under dual use research restrictions? Please check biosecurity documents and list of **select agents and toxins** (CDC): https://www.selectagents.gov/sat/list.htm	Not Applicable	
If you used a select agent, is the security level of the lab appropriate and reported in the manuscript?	Not Applicable	
If a study is subject to dual use research of concern regulations, is the name of the **authority granting approval and reference number** for the regulatory approval provided in the manuscript?	Not Applicable	

Reporting

The MDAR framework recommends adoption of discipline-specific guidelines, established and endorsed through community initiatives. Journals have their own policy about requiring specific guidelines and recommendations to complement MDAR.

Adherence to community standards	Information included in the manuscript?	In which section is the information available? (Reagents and Tools Table, Materials and Methods, Figures, Data Availability Section)
State if relevant guidelines or checklists (e.g., **ICMJE, MIBBI, ARRIVE, PRISMA**) have been followed or provided.	Not Applicable	
For **tumor marker prognostic studies,** we recommend that you follow the **REMARK** reporting guidelines (see link list at top right). See author guidelines, under 'Reporting Guidelines'. Please confirm you have followed these guidelines.	Not Applicable	
For **phase II and III randomized controlled trials**, please refer to the **CONSORT** flow diagram (see link list at top right) and submit the CONSORT checklist (see link list at top right) with your submission. See author guidelines, under 'Reporting Guidelines'. Please confirm you have submitted this list.	Not Applicable	

Data Availability

Data availability	Information included in the manuscript?	In which section is the information available? (Reagents and Tools Table, Materials and Methods, Figures, Data Availability Section)
Have **primary datasets** been deposited according to the journal's guidelines (see 'Data Deposition' section) and the respective accession numbers provided in the Data Availability Section?	Not Applicable	
Were **human clinical and genomic datasets** deposited in a public access-controlled repository in accordance to ethical obligations to the patients and to the applicable consent agreement?	Not Applicable	
Are **computational models** that are central and integral to a study available without restrictions in a machine-readable form? Were the relevant accession numbers or links provided?	Not Applicable	
If publicly available data were reused, provide the respective **data citations in the reference list**.	Not Applicable	
